# Making sense of the extraordinary – An investigation of the content and prevalence of appraisals of nonordinary experiences

**DOI:** 10.1371/journal.pone.0353126

**Published:** 2026-08-05

**Authors:** Pedro Fortes, Luiza Tavares, Gustavo Granjeiro, Giovanna Bortolini, Everton Maraldi, Tiago Bortolini, Larissa Hartle, Maria Lima, Maria Laport, Jorge Moll, Ronald Fischer

**Affiliations:** Cognitive Neuroscience and Neuroinformatics Unit, Institute D’Or for Research and Education, São Paulo, São Paulo, Brazil; Northumbria University, UNITED KINGDOM OF GREAT BRITAIN AND NORTHERN IRELAND

## Abstract

Individuals around the world report nonordinary experiences that stand out to them compared to their day-to-day or ordinary experiences. Yet, they can be appraised in very different ways, resulting in wellbeing and personal growth, or in marginalization and mental health problems. One challenge is that there are relatively few systematic efforts to explore possible appraisal processes and the overall contextual features of these experiences. In study 1, we report a validation and extension of a previously designed set of appraisal and contextual questions to the Inventory of Nonordinary Experiences, with a sample of Brazilian participants (N = 160). The study successfully validated all original appraisal categories with minor modifications and added nine new context and appraisal categories. In study 2, we conducted a prevalence study by administering these questions in a general population sample (N = 5,117) in which individuals reported on one of 38 nonordinary experiences. Most contextual features and appraisals followed a unimodal distribution approximating a normal distribution. This suggests that nonordinary experiences, however heterogeneous they may be, appear to be interpreted within the same set of beliefs and appraisals, in addition to sharing similar temporal characteristics. Furthermore, although 20% of participants reported an immediate negative impact and 25% reported some degree of suffering or discomfort from experiences, 51% evaluated experiences as promoting overall positive long-term life effects. These findings highlight the potential of human resilience and the complex role of nonordinary experiences vis-a-vis protective factors in promoting health. Our results provide new insights into consciousness states and their mental health and wellbeing implications.

Individuals the world over report experiences that fall outside their usual or day-to-day lived reality, such as suddenly hearing voices when nobody is around, feeling a connection to something greater, strong emotional experiences such as love, compassion or despair [1]. While these nonordinary experiences (NOEs) are often regarded as unusual, research indicates that these experiences are surprisingly common and intrinsic to the human condition [2]. Taves and Barlev [1] define such as “experiences that stand out to people or are marked by them as special… relative to what they consider ordinary or everyday, without assuming that the ordinary-nonordinary distinction is cross-culturally stable” (p. 52). As already implied by the authors, the interpretation of NOEs varies widely: some individuals may perceive them with euphoria and a sense of blessing, while others experience uncertainty and anxiety. Some individuals believe that the experience happens for a specific reason and may be caused by some supernatural entity whereas others think such an experience just happened by chance. Distinct from everyday experiences, NOEs can be highly valued in certain spiritual and religious traditions, often associated with spiritual growth and wellbeing. On the other hand, phenomenologically comparable experiences are being used in psychiatric manuals as symptoms of dissociative disorders or psychosis [3]. The interpretations that follow experiences are known as appraisals, which can be defined as “the outcome of a multi-level process that humans and other animals rely on when determining what is happening, that is, to interpret situations and events” [4]. Appraisals constitute a crucial factor for understanding the life impact of NOEs. Cultural and personal factors are likely to significantly shape these interpretations and lead to the framing of NOEs as religious, spiritual, paranormal, or pathological [5–8], which in turn may result in human flourishing or in mental health problems. Evaluation processes together with the context in which these experiences occur appear to play a critical role in determining outcomes associated with these experiences [9–11].

Instruments designed to measure these kinds of experiences frequently display *a priori* interpretations and theoretical frameworks embedded within the description of the experiences themselves, which often leads to a conflation of researchers’ own appraisals with the purely phenomenological account of the experience [4]. An example of an inherently spiritual/religious framework can be seen in the Daily Spiritual Experience Scale [12], which frames an experience of a nonordinary presence positively (“I feel God’s presence”). In contrast, a pathological framing of NOEs is observable in clinical scales such as The Oxford-Liverpool Inventory of Feelings and Experiences (O-LIFE) [13] which interprets similar experiences as traits of schizotypal personality and includes explicitly negative appraisals within the query about the experience (e.g., “evil presence”).

The Inventory of Nonordinary Experiences (INOE) has been developed by Taves and colleagues as a way to overcome this methodological hurdle by addressing subjectively recognizable features of experiences [1], with the explicit aim to broaden the scope for studying nonordinary phenomena. The inventory is based upon a subject-dependent definition of “nonordinary”, referring to nonordinary experiences as salient and special from the experiencer’s point of view, and relative to what they consider ordinary or not. The experience items were either adapted from previous scales or inspired by religious and spiritual literature ([4], p. 5–7). Along with these experience items, the INOE has included a small number of appraisals. Currently, the INOE features seven follow-up questions around the meaning-making processes and contextual features, such as the mental state of the individual when the experience occurred.

In this study, we aim to advance research on the measurement and prevalence of appraisals as well as situational factors surrounding a broad set of experiences [4,14] (as listed in S1 Table). Our first goal is to extend and validate INOE’s original list of appraisals and questions related to the context and timing of these experiences in Portuguese. Our extensions of the list of appraisals and the context focus particularly on a) the wellbeing implications of the experience, b) reasoning options around physical vs supernatural explanations of those experiences and c) further probing questions on the location and timing of these experiences.

The second major goal is to then query the prevalence and distribution of both the appraisals and contexts of these experiences, to see if there are patterns that can shed light on the cognitive processes involved in making sense of “experiences that stand out to people or are marked by them as special relative to what they consider ordinary or everyday” ([4], p. 4–5, emphasis in original text). In other words, when and where are subjectively salient experiences likely to happen? How do people make sense of these experiences? What is the perceived effect in the moment and over one’s life course? Most importantly, do appraisals show a uniform distribution across experiences – suggesting a common set of appraisals that is relied on when evaluating events – or do the distributions suggest more experience-specific processes? In addressing these two goals of adapting and expanding the list as well as exploring the prevalence and distribution of these experiences, our study aims to advance on a number of challenges in this broader area, including the notoriously difficult conceptual overlap between NOEs, wellbeing, and psychopathological constructs [1,14–16].

The INOE is useful for these purposes because it allows to distinguish between the phenomenological aspects of experiences and their appraisals. Consequently, the inventory offers opportunities to examine both potentially universal (etic) and culture-specific (emic) dimensions of lived experiences. The core focus of the inventory is on subjectively recognizable features of experiences instead of broader latent constructs, as is common in psychology. The experience items are formulated using generic language to enhance cross-cultural comprehension, while culturally loaded concepts are primarily addressed in the follow-up appraisal questions. In doing so, the inventory aims to separate the phenomenological features of experiences from culturally infused appraisals of experiences. The original version of the inventory contained 38 items that have been found to be understandable in English and Hindi [4]. These items were selected because the experience had been subject to conflicting interpretations in the literature that often involved strong claims about supernatural agency, extraordinary abilities or psychopathology and the relevant literature had suggested that these experiences often have profound positive or negative impact on people’s lives [4]. To date, these items have been grouped into 7 major conceptual groups, including Emotion (covering experiences such as joy, fear and awe), Sensory/Body (including perceiving lights, sounds or feeling touched), Sense of Self (including out of body experiences, absorbed or a diminished self), Presence (including feeling guided, visiting places or being in front of objects with apparent presence of some higher forces), Abilities (including déjà vu, extrasensory perception or having had a past life), Sickness/Health (near death experiences, unexplained rapid healing) and Meaning (including coincidences, receiving or perceiving messages or being able to have sudden deep insights). Of these, 31 items were recently validated in Brazil [14]. Maraldi and colleagues [16] extended the inventory to include a variety of dissociation and mediumship-like experiences for a Brazilian population. Similarly, five additional self-transcendent emotion experiences have been developed and validated [17]. Therefore, significant effort has been made to expand and validate the experience items resulting in an extended inventory covering a broad set of experiences that may be interpreted in highly conflicting ways (see S1 Table for a full list), but less effort has been made to explore and extend the probing questions around culturally salient appraisals or the specific contexts in which these experiences take place.

The published original version of the inventory [4] included a small number of appraisals which were deemed relevant for understanding the mental health impact or cultural significance. However, this list does not cover some of the important features that might be culturally salient or important for discriminating healthy from nonadaptive reactions. Indeed, a few studies have attempted to explore appraisals more broadly either at a qualitative or quantitative level [10,18–20] or have called for the extension of appraisals [15].

Our first major contribution is therefore an extension of the currently available appraisal options. Drawing on insight from two recent review studies that examined the role of nonordinary experiences on mental health [15] and a systematic review of dissociative experiences in Brazil [16] and taking inspiration from appraisal theory [9–11,21], we suggest and test additional appraisals that may be of importance, with a particular focus on possible mental health associations. We chose appraisal theory because this approach posits that emotions arise from the evaluations individuals make of events and situations, explaining variations in emotional responses across individuals and over time [10,22–24]. While different events may elicit similar emotional mechanisms at a neural level [25] the specific emotion experienced is influenced by the way those events are appraised within the specific context in which they occurred. Appraisals typically guide context-appropriate emotional responses; however, appraisals may also lead to nonadaptive responses [9,21,24]. The same phenomenological experience may result in personal thriving if appraised in a positive and beneficial manner or it may result in extreme cases in hospitalization and marginalization, if appraised as negative, intrusive, debilitating or not under the control of the individual [15,26]. Consequently, the nature and dynamics underlying appraisal processes in human experiences needs greater empirical scrutiny, especially with an emphasis on wellbeing and optimal functioning of humans within their day-to-day environment. The diverse nature of the experiences captured in the expanded INOE [4,14,17] provides a unique opportunity to capture highly distinct and possible conflicting appraisal processes.

To contextualize the appraisal processes, we focus on Brazil because it is the fifth-largest country, and one of the most diverse cultures in Latin America in terms of ethnic and religious expressions [27,28], but it remains under-researched considering the size of the population [29,30]. Brazil is also a good case study because numerous religious traditions explicitly involve different sets of nonordinary experiences in their spiritual repertoire and practice. These experiences have been studied in the literature from either a spiritual-religious or a psychopathological psychiatric or clinical perspective [4,15]. To better understand how individuals make sense of and cope with any unusual experiences, it is important to probe spiritual and religious but also mental health related appraisals further. The extent to which experiences lead to personal growth vs mental health problems to some extent is dependent on the availability of meaning making systems within one’s environment. Given the diversity of meaning-making systems available in Brazil, it offers a unique research context that can help in understanding collective vs individual meaning making strategies. Here, we draw inspiration from a proposed distinction between an experiential source (or common core) hypothesis versus a cultural source hypothesis [31] of nonordinary experiences. The distribution of the contextual features and appraisals across a wide range of nonordinary experiences can provide some first important insights into the role of person-idiosyncratic vs culturally shared meaning making systems.

A central challenge in such an endeavor is to validate meaning-making processes within specific cultural contexts, that is, finding appropriate phrasings of context information and appraisals to capture the locally salient understandings of human experiences. Therefore, an important feature of our work is the use of a mixed-methods approach involving both qualitative and quantitative analyses for examining the comprehensibility of individual appraisal options [14,32–34].

Once we have preliminary validity evidence that individuals understand the appraisals as intended, an exploration of the relative prevalence of different appraisal processes can help to contextualize the circumstances and impact of nonordinary experiences on individuals and communities. For example, if a particular appraisal is found to be widespread across a range of distinct experiences, it may suggest shared interpretive frameworks that could help people make sense of experiences in general, suggesting a collective meaning making process. Together, these complementary mixed-method analyses provide new insights into the general contextual features and associated appraisal processes around nonordinary experiences.

Furthermore, validating new appraisal items for culture-specific contexts and measuring the prevalence and distribution of meaning-making motifs has implications that extend beyond academic research. Gaining insight into how individuals and communities experience, appraise, and are affected by nonordinary states can inform clinical practice and contribute to improved wellbeing outcomes [33]. Such outcomes may be facilitated by a critical re-examination of present clinical paradigms, which often pathologize nonordinary states *a priori* [15].

## Study 1 – Validation and extension

The original version of the INOE contained seven follow-up questions with a number of appraisals. Two appraisals (impact, life effect) questioned the overall impact and the direction of the impact on people’s lives. These two questions are important for evaluating the possible mental health consequences of specific experiences. Another question focused on the mental state of the individual when the experience happened. It included questions related to the use of drugs or alcohol, mental or physical illness, sleep related states, dreams or other states. The question about drugs and alcohol are often taken as exclusionary criteria when asking about possible pathological implications of these states [15,34] therefore it is important to include these. An interesting observation is that most individuals in the two validation samples in the original study listed ‘none of the above,’ implying that psychoactive substances, health problems, sleeping or dreaming were less important for most of the participants reporting their experiences. This question therefore is important for evaluating contextual features of the experience.

The other four questions focused on the possible causes and reasoning about the ontological nature of the experiences. Given the research interest of the developers of the inventory [1], the focus was on differentiating supernatural from scientific reasoning about the experiences. First, there was a question about whether the experience itself is considered spiritual or religious (or not). Second, there was a query whether science might be able to explain these experiences, followed by questions on why the individual may have experienced this particular event (e.g., destiny, to be rewarded or punished, as a message) as well who may have caused it (e.g., god, spiritual beings). A substantial proportion of participants in the original validation study indicated that pure chance could constitute a valid explanatory reason. For the causal agent, most participants rejected the idea that gods or spirits may be responsible. However, these data were not systematically evaluated across all experiences in a larger sample, so we are not in a position to examine (the perceived lack of) implied causal agents more broadly.

As should be clear, the current version of the inventory has a relatively restricted set of appraisals considering the possible appraisal dimensions that might be relevant for understanding the mental health impact or cultural and spiritual significance. There are relatively few questions on the situational features of where and when the experience occurred. Assessing the context in which NOEs unfold is fundamental for understanding how environmental factors might shape the experience on both phenomenological and interpretive levels, as well as its emotional outcomes [11,15,31,34,35]. There are also relatively limited questions on the immediate and long-term emotional reactions that we need in order to understand the medium and long-term mental health implications. Without a clearer understanding of both relevant appraisals as well as contextual features of where and how such experiences took place, it becomes challenging to advance our understanding of protective and risk factors of mental health [35]. Therefore, our first attempt is to broaden this list and add additional categories that help characterize experiences and appraisal processes in our study context to advance future mental health relevant research.

### Extension of the nonordinary experiences appraisals

To broaden the set of follow-up questions about the context, nature and appraisal processes, we relied on multiple sources. First, we took advantage of a recent systematic review of the empirical literature on nonordinary experiences and health [15]. This review identified 725 articles, of which 157 reported empirical data on nonordinary experiences and health correlations and were therefore reviewed in more detail. This provided a source of possible appraisals that might be relevant for understanding and contextualizing health effects. Second, we conducted a systematic review of empirical research on ethnographic, sociological, medical and psychological research on mediumship/possession and dissociation in Brazil [16], resulting in a list of 961 articles and books about dissociation-like experiences. We then reviewed a subset of 52 documents which reported ethnographic descriptions as well as social and clinical case studies of these experiences. Sourcing relevant literature via these two systematic reviews was crucial, as it allowed us to identify key themes in the description of these experiences but also gaps and inconsistencies in the current research on appraisals of such experiences. By synthesizing previous findings, we were able to better understand how these phenomena have been conceptualized, the methods used to study them, and the cultural influences that shape their interpretation.

Drawing on this material, we developed nine new appraisal questions to capture aspects of nonordinary experience that are both culturally relevant to the Brazilian context and theoretically meaningful within the broader literature on nonordinary experiences, appraisal theory, and cognitive psychology. A total of 28 previous measures of nonordinary experiences and their associated features and appraisals, identified in our systematic review on nonordinary experiences and health, also informed the construction of these extended items [15].

Focusing on the specific items added, available evidence suggests that the physical environment (e.g., nature, hospital, religious ritual) may influence the way nonordinary experiences are both perceived and appraised, thereby impacting how they may relate to wellbeing [11,15,36–38]. Accordingly, one of the nine new appraisal items was dedicated to the context in which the experience took place.

Three new items focused on temporal features of experiences: frequency, duration, and onset. These additions were motivated by two main factors. First, there is a notable lack of studies examining the age of onset and developmental aspects of nonordinary experiences [15,39,40] and how age of the first experience relate to possible beneficial or detrimental effects on wellbeing and health. The developmental factor may be especially relevant when considering the clinical assessment of nonordinary experiences in association with trauma exposure [41]. Second, a number of studies [42–45] emphasized the differential impact of frequency and duration on the perception, memorization, appraisal, and emotional response to lived events. Therefore, we considered it important to separate these temporal dimensions for better understanding how nonordinary experiences might elicit different responses and health outcomes.

Furthermore, we added three new appraisal items that directly assess the momentary effect and emotional response to the experience (ranging from joy to distress) in the moment it unfolded, as well as the perceived evaluation of the experience by significant others (family, friends, community). These questions extended the original subgroup of appraisals focused on life effect and impact (4). Relevant work has demonstrated [26,46,47] that the emotional landscape of the experience in the moment, and whether it integrates or distracts from daily tasks makes these questions important for a better understanding of the longer-term effects of the experiences. Similarly, the social framing and cultural expectations surrounding nonordinary experiences seem to be associated with how the experiencer appraises and responds to them, modulating the quality of the experience itself and its subsequent integration into ordinary life [48,49]. There is also ethnographic and qualitative evidence that, even when the experience is disturbing and distressing, experiencers may develop more adaptive coping strategies within the context of community-embedded spiritual practices and rituals [50].

Finally, we added two new appraisal items regarding experiencer agency and control of the experience. This decision was informed by previous evidence showing that the controllability of the experience is a relevant predictor of distress and pathological outcomes [26]. This is further corroborated by ethnographic case studies [3,50], which highlight the role of social rituals and religious gatherings as means of learning to control experiences and develop intentionality over them, consequently reducing distress and enhancing wellbeing [15]. A study by Moreira-Almeida and Cardeña (2011) suggested that one important criterion for differentiating between pathological and non-pathological nonordinary experiences is that healthy experiences can generally be controlled by the individual [15,26,51]. For this reason, it may be useful to understand the level of attempted control during the experience and its associations with levels of distress in the moment, long-term effects, and other appraisals.

We decided to divide all appraisal items into five major groups of questions, asking: 1) about the reasoning or belief about the experience (largely following the original set of questions), 2) a set of inquiries about the effect or impact of the experience both in the moment and over the long term as well as 3) the extent of experiencer agency within those experiences which allows us to query the mental health implications in greater detail, 4) the context within which the experience took place and 5) the temporal nature of the experiences.

Table 1 presents an overview of this extended list. More detailed information on the scientific and empirical literature that grounds this new and extended set of questions is available in the supplement (S1 Text) together with the complete list of appraisal items and their response options (S3 Table).

**Table 1:** Description of extensions and additions to contextual and appraisal items.

Group	Item	Definition	Extensions / additions
**Situational**	Mental state †	Perceived mental state at the time of the experience	Adapted to better separate dream states, awake and alert state [52,53], and now includes substances (to avoid the negative stereotypes of drugs)
	Context *	Exploration of activities that have been discussed in the literature as inducing nonordinary experience	New item: Lists a number of activities, contexts and locations that have been discussed in the literature for their potential to induce nonordinary states – e.g., religious ritual, music, being in nature, sensory deprivation [54–57]
**Temporal**	Duration *	Perceived duration of the experience	New item: Longer lasting experiences may interfere with associated emotional processes [45]
	Frequency *	Remembered frequency of the experience	New item: More frequent experiences may be more interruptive and interfere with daily functioning [15,58,59]
	Onset *	When did the experience first emerge	New item: Earlier onset may imply more maladaptive long-term effects [40,60–63]
**Reasoning/belief**	Agent †	Extent to which various supernatural agents may be causing the experience	We extended this list including broader supernatural and social causes [64–70]
	Reason †	Possible supernatural reasons for the experience (e.g., sign, reward/ punishment, destiny)	The literature indicated the importance of abilities, social roles or responsibilities [52,71]; also added life purpose to clarify destiny [72]
	Spiritual/religious †	Extent to which an individual appraises the experience as being of a spiritual or religious nature	Added an ‘I don’t know’ option [73–75]
	Science	Extent to which science can explain the experience	Unchanged.
**Effect/impact**	Effect moment *	Extent to which the experience facilitated or interfered with other activities while happening	New item: Assesses the possible disruptive nature of the experience [26,46,47]
	Distress *	Extent to which the experience caused joy and wellbeing vs discomfort or suffering	New item: similar to life effect but focusing more clearly on the wellbeing aspect [15]
	Life effect †	Extent to which the experience had positive or negative lasting effects (valence) on the person’s life and beliefs	More explicit statement of the lasting nature of the effects [46,76]
	Impact	Extent to which an individual was affected by the experience	Unchanged.
	Community *	The perceived evaluation by significant others (family, friends, community)	New item: Stresses the social evaluation aspect through the perceived evaluation by others [31,48,49]
**Agency/control**	Control *	Extent to which an individual was able to control the experience	New item: Controllability has been discussed as central for distinguishing healthy vs maladaptive experiences [26]
	Induction *	Extent to which individuals tried to induce or avoid the experience	New item: Focuses on the agency and desirability of the experience [20]

† Original items with extended response options.

* New item added in this study.

The first challenge for any survey is that it needs to be validated. These appraisals were designed by academic researchers holding postgraduate degrees, and using insights from ethnographic, medical, and psychological research reported in the literature. Individuals from a general population sample that are immersed in specific communities that do not share the specific secular-scientific perspectives and are unfamiliar with the broader literature may not understand the items or questions in the same way that was intended by the researcher. Therefore, a first challenge for a more accurate understanding of relevant appraisal processes in the general population is to carefully examine the content validity of the questions.

Given the specific nature of the appraisal follow-up questions, they focus on specific events in the person’s life and are not written to capture underlying latent dimensions. As a consequence, the standard methodological tool kit for examining the validity and reliability of the measurement instruments using traditional psychometric criteria is not applicable. Each appraisal needs to be examined in its own right, because there are no alternative questions that allow an investigation of the dimensionality, internal consistency and estimation of the true score component.

We therefore decided to use a qualitative method from a larger tool kit of cognitive interviewing and item probing [77,78] called Response Process Evaluation method (RPE). The RPE is a structured qualitative approach used to validate survey items through web-based probes [4,32]. It focuses on understanding the cognitive processes individuals use when engaging with survey items, ensuring that the questions are understood as intended and that the responses accurately reflect their perspectives. This is achieved by transforming traditional cognitive interviews into a meta-survey format, where participants answer open-ended probes that require them to give examples that fit the description, paraphrase items, defining key terms, or explaining their response choices instead of completing the full survey [32].

Responses are evaluated and coded based on whether the item was understood, that is, participants’ answers imply that they interpreted the question in line with its intended meaning. The process is iterative: items are tested in small batches, revised if needed, and retested until they achieve consistent interpretability. An item is considered provisionally validated when at least 80% of evaluations are coded as “understood” or “likely understood” [79]. This method combines the efficiency of crowdsourcing with the depth of cognitive interviewing, ensuring that the resultant items are clear, valid, and aligned with their intended meaning, thereby achieving conceptually meaningful instruments.

### Methods

#### Participants.

Participants were 160 Brazilian adults (aged 18 years and above) who (a) were registered with an online survey panel company (Netquest.com), (b) consented to participate and (c) were fluent Brazilian Portuguese speakers. In total, 288 participants successfully completed the Informed Consent Form, but 75 discontinued participation before completing the questionnaire. Of the remaining 213 participants who completed the full interview, 28 were excluded for providing random answers unrelated to what was being asked - e.g., random letter and number sequences. An additional 25 participants were excluded for providing uninterpretable or excessively vague responses – e.g., single word answers such as “that” or “yes”. Thus, the final sample consisted of 160 participants. No demographic data was collected due to time restrictions in combination with a coding error. The study was approved by the Research Ethics Committee of the D’Or Institute for Research and Education – IDOR (CAAE: 65573322.6.0000.5249). Sample size was determined by the stopping criteria described in the section entitled ‘Decision criteria for item validation.’ We followed the original Response Process Evaluation Protocol [4,32], which specified that an item can be considered provisionally validated if 80% of at least 20 participants have understood the appraisal question. Using the number of appraisal items to evaluate and assuming an 80% Validity Score threshold, we would have needed a minimum of 40 participants.

### Instruments

#### The inventory of nonordinary experiences.

We used the follow-up questions of the Inventory of Nonordinary Experiences (INOE, [4]), which distinguishes between the phenomenological aspects of experiences and their appraisals. Our starting point was the original set of appraisal items from INOE. As shown in Table 1, we kept the impact, life effect and science questions unchanged from the original INOE. Some minor wording adjustments were made during the translation.

For the other original appraisal categories, new response options were added (see Table 1). In the mental state question, we added the option of being “awake and alert” during the reported experience. For the spiritual/religious question, an “I don’t know” response option was included. For the reason question, another 2 response options (“life purpose and mission,” “abilities, responsibilities or roles”) were added. For the agents question, we added 9 new response items: “holy spirit”; “angel(s) or saint(s)”; “demon(s)”; “unknown beings, spirits or forces”; “extraterrestrials”; “energy/energies”; “one or more known or unknown individuals”; “someone who prayed or sent you good intentions”; and “I don’t believe anyone made me have this experience.” We created new appraisal categories that were derived from the literature and adapted for the Brazilian context, as recommended by Maraldi et al. [15] (see Table 1). As mentioned above, our aim was to capture more information on the context and possibly important features in order to facilitate future research focused on the impact of nonordinary experiences on wellbeing and mental health of individuals and communities. Further elaborations on the development and further literature are presented in the supporting information (S1 Text). S2 Table shows all the modifications made to the original appraisal list from [4] and S3 Table shows the full list of appraisal and context items.

#### Translation.

The original questions were translated from English into Brazilian Portuguese using a parallel committee translation approach following the TRAPD guidelines [80]. This method has been shown to be superior [81,82] compared to more commonly used translation and back translation approaches.

#### Response process evaluation.

**Fig 1:**
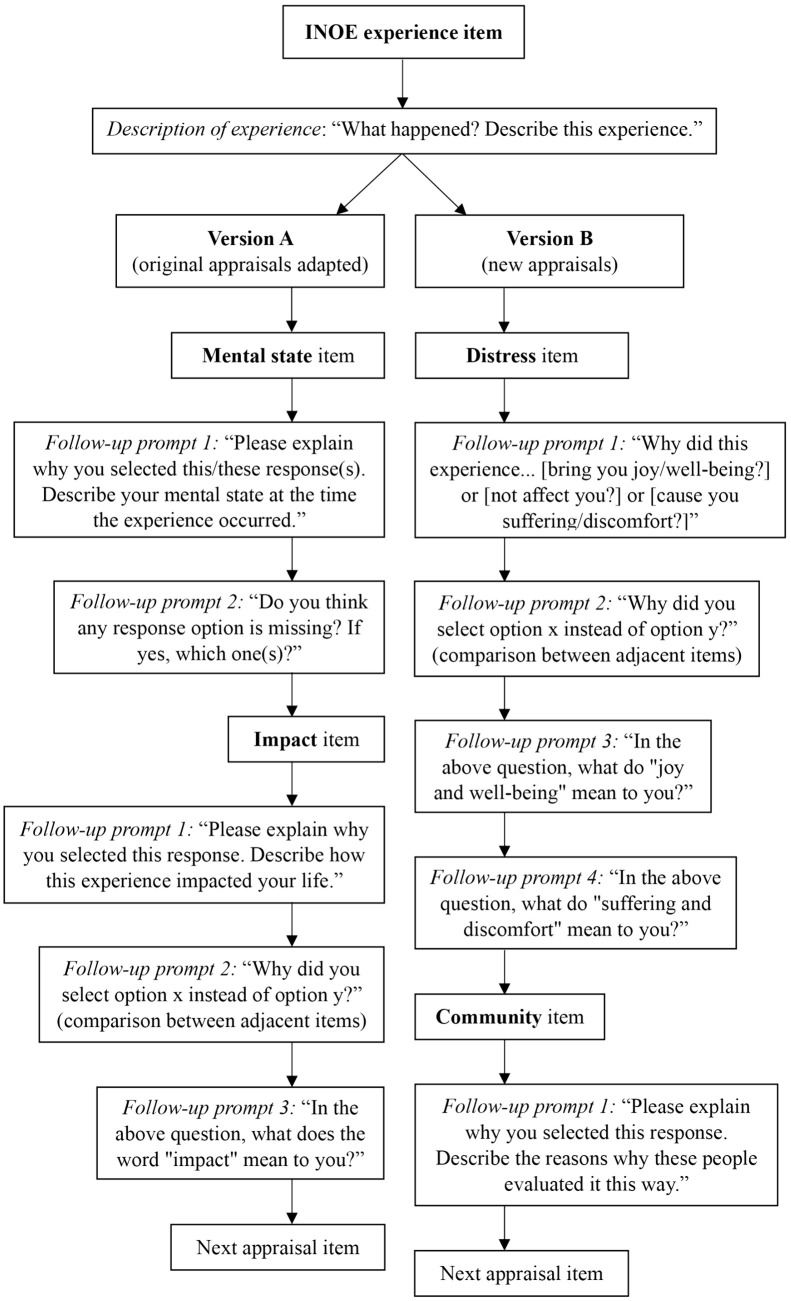
Flowchart of the RPE process with prompt examples for appraisal items.

We used the Response Process Evaluation protocol [32], which is a form of iterative web-probing [78]. After agreeing to participate and completing an online consent form, individuals from an online panel were shown one experience item and were asked if they have had the experience. If yes, the participants were shown appraisal probes. We randomly presented either the adapted original appraisals (7 items) or the newly developed appraisal questions (9 items). We decided to split the validation process for the original (version A) and new items (version B), because of the time and effort involved in answering all the probing questions – see Fig 1 for a flowchart of our RPE process with two examples of appraisal items and their corresponding probes for each version. If individuals reported having had the experience more than once, we asked them to answer any of the following questions considering the most significant or outstanding experience. If a participant reported not having had any of the experiences presented, the person was thanked and the study ended.

Participants were first instructed to provide a brief description of their specific nonordinary experience and then evaluate the subsequent appraisal items based on that experience. They were asked to provide explanations for their chosen response to each appraisal question. For appraisals with ordinal response options, participants were also required to justify their selection of one option over an adjacent one. Furthermore, participants were given an open-ended probe at the end to inquire whether they perceived any response options as missing or incomplete. The validation protocol used in our study was preregistered (see https://osf.io/ze6cb/overview, [14]).

Regarding missing data, no case level imputation was applied since incomplete interviews were not retained in the final sample. At the item level, missingness in the dataset reflects the randomization either to the original (version A) or new appraisal items (version B), as illustrated in Fig 1. Items from the non-assigned version were not administered and are therefore structurally missing. Within each version, participants were required to respond to the complete set of appraisal items as part of the RPE protocol, so there was effectively no item non-response and no item-level imputation.

#### Decision criteria for item validation.

To evaluate whether an appraisal was understood as intended, we used the adapted RPE protocol and calculated Validation Scores for each response. A team of four native Brazilian Portuguese speakers coders independently evaluated whether the experience item was understood as intended using a five-point scale, where 1 = Understood, 2 = Probably Understood, 3 = Not Enough Information, 4 = Probably Not Understood, and 5 = Not Understood. To facilitate the coding process, we transformed the five-point responses by each rater into a simplified three-point Understood/Not Understood/Unclear response category. Subsequently, we applied rules to make a decision on each response [14]: if there is a 75% majority decision (3 out of 4 evaluators agree on the understanding of a response), the majority choice was selected. If there was an even split between the evaluator team and there was no majority for either understood or not understood, the response was classified as Unclear/Requiring further information. If there was no clear pattern (e.g., two raters voted for understood, one rater voted for not understood, one rater voted for unclear response), an adjudicator made a final decision on the evaluation of the response. The adjudicator took final responsibility for classifying the response as “understood,” “not understood” or “not enough information,” taking into consideration the comments by the panel members.

The validation of the appraisal item required a Validation Score (U/(U + NU)) of at least 80% in a sample of 20 participants or more. Following the original protocol, for these calculations, unclear responses were ignored. The process proceeded in batches of 10 responses at a time per item. Once an appraisal reached this threshold, the process was stopped and the appraisal item was considered conditionally validated. If the appraisal item did not reach this 80% threshold, responses were examined and the item was modified to improve clarity and precision. This process repeated until an item passed the threshold or until five modifications of an item were attempted. Therefore, our sample size was determined by the relative understandability of the appraisal items within the batches of 10 responses. See Taves et al. [4] for more details on the process.

### Results

Table 2 shows the overall Validation Score together with the information from the original validation in the US sample study for comparison purposes. All the original follow-up appraisal questions were validated with minor phrasing adjustments. The additional response item within the Mental state category was validated, as well as in the new Spiritual/religious, Reason and Agent categories.

**Table 2:** Full item wording and understanding rates for original and extended INOE appraisal and context items.

Group	Item	Query & Response Options	Proportion Understood (BR)	Proportion Understood (US, Taves et al. 2023)	Validation Score (BR)	Unclear Responses (BR)	Unclear Responses (US)
**Situational**	**Mental State** †	When you had this experience, you were... (select all options that apply)	**64/81 (79%)**	**139/139 (100%)**	**98.5**	**4**	**0**
	1A †	Awake and alert	45/55 (81.8%)	NA		0	NA
	1B	Under the influence of alcohol or substance(s)	1/2 (50%)	13/13		0	0
	1C	Suffering from a mental or physical illness	1/1 (100%)	9/9		0	0
	1D	Falling asleep, waking up, or exhausted	6/8 (75%)	6/6		0	0
	1E	Sleeping (dreaming)	5/8 (62.5%)	9/9		0	0
	1F	None of the above	6/10 (60%)	83/83		4	0
	**Context** *	When you had this experience, were you... (select all options that apply)	**71/79 (89.9%)**	NA	**97.3**	6	NA
	9A	Listening to music	4/7 (57.1%)	NA		3	NA
	9B	In a religious or spiritual context	10/11 (90.9%)	NA		0	NA
	9C	In a context that made it difficult for you to perceive what was happening (e.g., with a lot of noise, smoke, darkness)	3/3 (100%)	NA		0	NA
	9D	In a hospital or healthcare clinic	16/18 (88.9%)	NA		0	NA
	9E	In an unknown or new place	2/4 (50%)	NA		1	NA
	9F	In nature or a forest	4/8 (50%)	NA		0	NA
	9G	Alone	13/23 (56.5%)	NA		2	NA
	9H	None of the above	19/21 (90.5%)	NA		0	NA
**Temporal**	**Duration** *	Approximately how long did the experience last?	**71/79 (89.9%)**	NA	**97.3**	6	NA
	15A	Very quick, almost instantaneous	3/3 (100%)	NA		0	NA
	15B	A few minutes	12/14 (85.7%)	NA		2	NA
	15C	Around an hour	6/7 (85.7%)	NA		0	NA
	15D	More than an hour	15/17 (88.2%)	NA		2	NA
	15E	Days	20/22 (90.9%)	NA		1	NA
	15F	I don’t know	15/16 (93.8%)	NA		1	NA
	**Frequency** *	How many times have you had this experience in your life?	**66/79 (83.5%)**	NA	**98.5**	12	NA
	8A	1 time	16/21 (76.2%)	NA		5	NA
	8B	2 - 3 times	27/34 (79.4%)	NA		6	NA
	8C	4 - 5 times	5/5 (100%)	NA		0	NA
	8D	6 - 10 times	5/5 (100%)	NA		0	NA
	8E	More than 10 times	13/14 (92.9%)	NA		1	NA
	**Onset** *	When did this experience happen in your life? (If you have had it more than once, please indicate the period when it started happening to you.)	**75/79 (94.9%)**	NA	**98.7**	3	NA
	16A	Childhood	3/4 (75%)	NA		0	NA
	16B	Adolescence/Youth	15/16 (93.8%)	NA		1	NA
	16C	Adulthood	55/57 (96.5%)	NA		2	NA
	16D	I don’t remember	2/2 (100%)	NA		0	NA
**Reasoning/belief**	**Agent** †	Who, if anyone, made you experience this? (Select the most important option)	**63/81 (77.8%)**	**83/84 (99%)**	**100.0**	**6**	**0**
	7A	God or gods	15/19 (78.9%)	13/13		0	0
	7B †	Holy Spirit	2/2 (100%)	NA		0	NA
	7C †	Angel(s) or saint(s)	1/2 (50%)	NA		1	NA
	7D †	Demon(s)	NA	NA			NA
	7E †	Ancestors or other known beings, spirits, or forces (e.g., entity, orixá, etc.)	2/2 (100%)	14/14		0	0
	7F †	Unknown beings, spirits, or forces	NA	14/14			0
	7G †	Extraterrestrials	0/1 (0%)	NA		0	NA
	7H †	Energy/energies	3/5 (60%)	NA		2	NA
	7I †	One or more known or unknown individuals (someone who cast a spell, hex, evil eye, or had ill intentions towards you)	1/1 (100%)	NA		0	NA
	7J †	Someone who prayed for you or sent you good intentions	NA	NA			NA
	7K †	Other(s)	4/4 (100%)	56/57		0	0
	7L †	I don’t believe someone made me have this experience	35/45 (77.8%)	NA		3	NA
	**Reason** †	Why do you think this happened to you? (Choose the closest response)	**64/81 (79%)**	**121/121 (100%)**	**100.0**	**6**	**1**
	6A	To give me a sign or a message	12/15 (80%)	27/27		0	0
	6B	To reward or punish me for my actions	1/4 (25%)	10/10		1	0
	6C	Because of my destiny or life purpose/mission	17/19 (89.5%)	16/16		1	0
	6D †	Because of my abilities, responsibilities, or roles	7/8 (87.5%)	NA		0	NA
	6E	None of the above (it may have been by chance)	27/35 (77.1%)	68/68		4	1
	**Spiritual (S/R)** †	Do you consider this experience to be spiritual or religious?	**62/81 (76.54%)**	**NA**	**98.4**	**6**	**NA**
	4A	Yes	26/32 (81.2%)	NA		1	NA
	4B	No	25/35 (71.4%)	NA		2	NA
	4C †	I don’t know	11/14 (78.6%)	NA		3	NA
	**Science**	Do you think science can explain how this experience occurred?	**63/81 (77.77%)**	**85/87 (98%)**	**98.4**	**6**	**4**
	5A	Yes, science can or will be able to explain it	36/48 (75%)	51/51		4	0
	5B	No, something else is involved	27/33 (81.8%)	34/36		2	4
**Effect/impact**	**Effect Moment** *	What was the effect of this experience when you had it?	**69/79 (87.3%)**	NA	**97.2**	6	NA
	10A	It facilitated, helped, or complemented what I was doing	23/27 (85.2%)	NA		2	NA
	10B	It disturbed, interfered with, or interrupted what I was doing	26/26 (100%)	NA		0	NA
	10C	It had no effect or interference	20/26 (76.9%)	NA		4	NA
	**Distress** *	How much would you say this experience caused you something positive (joy/wellbeing) or negative (suffering/discomfort)?	**73/79 (92.4%)**	NA	**100.0**	6	NA
	11A	A lot of joy or complete wellbeing	27/28 (96.4%)	NA		1	NA
	11B	Some joy or wellbeing	11/12 (91.7%)	NA		1	NA
	11C	Neutral, it didn’t cause me anything	3/5 (60%)	NA		2	NA
	11D	Some suffering or discomfort	13/13 (100%)	NA		0	NA
	11E	A lot of suffering or discomfort	19/21 (90.5%)	NA		2	NA
	**Life Effect** †	In general, the long-term effect of this experience on your life and beliefs was:	**66/81 (81.5%)**	**144/147 (98%)**	**100.0**	**4**	**1**
	3A	Very positive effect	29/36 (80.6%)	48/50	100.0	1	1
	3B	Slightly positive effect	9/12 (75%)	30/30		1	0
	3C	Neutral or no effect	18/22 (81.8%)	51/51		2	0
	3D	Slightly negative effect	9/10 (90%)	6/7		0	0
	3E	Very negative effect	1/1 (100%)	9/9		0	0
	**Impact**	In general, what was the impact of this experience on your life?	**68/81 (84%)**	**42/43 (98%)**	**100.0**	**2**	**0**
	2A	Little or no impact	15/18 (83.3%)	16/16		0	0
	2B	Some impact	12/17 (70.6%)	10/10		1	0
	2C	Significant impact	41/46 (89.1%)	16/17		1	0
	**Community** *	In general, how would your friends, people in your community, or your family evaluate the fact that you have had this experience?	**74/79 (93.7%)**	NA	**98.7**	4	NA
	12A	Positively	27/28 (96.4%)	NA		1	NA
	12B	Neutral	32/35 (91.4%)	NA		2	NA
	12C	Negatively	15/16 (93.8%)	NA		1	NA
**Agency/control**	**Control** *	To what extent were you able to control this experience?	**68/79 (86.1%)**	NA	**98.6**	10	NA
	13A	I had complete control over it	23/27 (85.2%)	NA		3	NA
	13B	I had little control over it	18/23 (78.3%)	NA		5	NA
	13C	I had no control over it	18/19 (94.7%)	NA		1	NA
	13D	I did not attempt to control it	9/10 (90%)	NA		1	NA
	**Induction** *	After experiencing this for the first time, did you try to have or induce this experience again?	**68/79 (86.1%)**	NA	**95.8**	8	NA
	14A	Yes, I tried	20/22 (90.9%)	NA		0	NA
	14B	No, I tried to avoid the experience	18/19 (94.7%)	NA		1	NA
	14C	No, I neither tried to avoid nor induce it	30/38 (78.9%)	NA		7	NA

† Original items from [4] with extended response options developed for this study.

* New items added in this study.

For the nine new appraisal categories, all items were successfully validated with an understanding rate of at least 83.5%.

### Discussion

We successfully validated all original appraisal categories with minor wording modifications and additional response options for some of the questions. We also validated nine new context and appraisal categories. Taken together, this expanded set is grounded in the wider literature on nonordinary experiences and was designed to help with a more careful differentiation of the situational context and appraisal processes to understand the possible mental health impact of these experiences. For example, the extended agent and reason question together with the induction question may help in understanding appraisal processes in relation to health. Work from a folk psychology perspective has suggested that people tend to give reasons to events perceived as intentional, as these are understood as actions of agents with goals. For example, an individual may perceive that they had an experience because a supernatural power wanted to send them a message. In contrast, events considered unintended tend to have perceived causes, but not reasons, being explained by a chain of antecedents rather than by the attribution or intentionality [20,83]. An individual may attribute an experience to a chance event or to some medical condition. As a result, the extended set of questions applied in future studies can help in explicitly testing these observations across a wider set of experiences and provide new insights for health-related appraisal processes.

Our work was based on academic research but focused on Brazilian linguistic and cultural idiosyncrasies concerning how nonordinary experiences might be commonly experienced, understood and interpreted. Future studies in other contexts may evaluate whether additional response categories may be necessary or whether some of the response categories need to be modified or adapted to different local contexts.

One issue is that some of the categories were selected by a relatively small number of participants. This means that we have less confidence about some of the rarer appraisals. However, at the level of the overall context and appraisal question, our data suggests that individuals understood the questions. Low frequency responses need to be interpreted with greater care. One important limitation of our study was that no information on demographic was available. This implies that we cannot make strong claims about the external validity and generalizability of our validation study. Comparative work using this panel service and samples invited through similar methods used in this study suggested that samples tend to be representative of the larger population in terms of age, gender and geographic location, but are typically more well educated than expected based on census data [14].

One important question that arises from our data and the observations of the data in Taves et al. [4] is the overall distribution of the appraisal prevalences. How common are different appraisals across a wider set of experience items? Are there common cognitive patterns that individuals use to make sense of experiences that may be intense or unusual within an everyday environment? This is the purpose of the next study.

## Study 2 – Population level prevalence of appraisals across Brazilian adults

The previous study reported on the expansion and validation of a set of appraisals of relevance for nonordinary experiences. To date, no study has examined how frequent or prevalent different appraisals of such experiences are in the general population. Extant research has shown that these experiences themselves are rather common in the general population, much more than clinical studies that examine the pathological side of some of these experiences would imply [14,15]. To the best of our knowledge, there is little systematic and quantitative research at the population level that examines which of the possible appraisal and meaning-making processes are more or less likely across a wider set of nonordinary experiences. This is the aim of our second study. We make use of the validated list of experience items of INOE [4,14] and an extended set of items that query altered states of consciousness common in mediumistic and spirit-possession contexts [16] as well as transcendental emotion experiences [17]. The overall list contains positive and negative emotional experiences, changes in cognitive, perceptual and body systems, changes in the experience of meaning in life, changes in the experience of the self as well as questions about experiences of sickness and health. These experiences therefore draw upon a wide set of cognitive, perceptual, emotional and bodily processes and systems, which offers a unique opportunity to probe meaning making efforts in general.

Drawing upon work on the nature of nonordinary experiences, there are two major hypotheses that have been put forward about nonordinary experiences more broadly and which may help in predicting possible patterns in the prevalence of context features and appraisals: an experiential source (or common core) hypothesis versus a cultural source hypothesis [31]. The former hypothesis is based on observations that nonordinary experiences across history and across different cultural contexts often share certain regularities and features that suggest the presence of a common core of specific experiences. There are very diverse theoretical positions associated with this hypothesis, with biological and evolutionary approaches representing the most universalistic position [84–90]. The basic argument is that common neurobiological features of the brain and the broad perceptual system of humans have evolved through evolutionary pressures to adapt to situational contexts. Evolutionary processes always create variability, which then allows selective pressures to operate. By definition, this implies that there are misperceptions or miscategorizations triggered by features of the neurocognitive system in response to situational input that then give rise to nonordinary experiences in some individuals. Because the experiences themselves are likely linked to very diverse neurocognitive processes ranging from emotions to alterations in the perceptual system to self-related processes, we may expect a highly diverse set of appraisals that are invoked by participants to make sense of these experiences. As discussed by Taves et al. [4], the experience of these events is subject-dependent, based on the specific neurobiological characteristics of the individual [91] and therefore, different individuals may or may not share the same deep personal insight that is at the core of the item. As a result, we may not expect a clear pattern in both the situational features and the appraisals that individuals attribute to these diverse experiences. As noted above, experiences are likely driven by specific neurobiological mechanisms that are thought to vary widely across experiences and are then interpreted by individuals based on their idiosyncratic life histories. As a result, we may not expect strong or coherent patterns in the distribution of situational features and appraisals across this diverse and broad set of experiences included in our study.

A second major hypothesis is the cultural source hypothesis [92]. Within this perspective, experiences are conditioned via sociocultural learning processes during a person’s socialization within a specific sociocultural context. During this learning process, cognitive schema are developed and then applied across different contexts to make sense of the world within an overall cognitive framework. This cultural source hypothesis is not so much a well-established theoretical position but rather a basic assumption that often remains implicit within the sociological, anthropological and ethnographic literature on nonordinary experiences [31]. It presumes that culturally shared meaning-systems give rise to cognitive schema that are then applied across different contexts and experiences. Given the efficiency of a smaller number of schemas that are reasonably fuzzy but can be applied across a larger number of occasions, we may expect unimodal distributions around appraisals across a wider set of experiences.

Of particular importance for this argument is the fact that Brazil is a highly religious country and individuals are repeatedly exposed to religious and spiritual explanations which may influence how individuals experience and interpret a variety of experiences. The issue here is not so much the religious orientation of the individual per se, but rather the importance that religious and spiritual interpretations play in society in general. Therefore, the emergence of normally distributed reports of both situational features and appraisals across this wide range of experiences suggests a possible presence of shared cognitive features that help individuals make sense of events independent of the specific experience. Examining the distribution of the contextual features (context and temporal characteristics across experiences) vs the appraisal processes (reasoning and beliefs, mental health impact) may offer possible insights into contributing processes.

In summary, our second study explores the distribution of appraisals across a broad set of diverse experiences. Based on the experiential source hypothesis and the wide range of experiences studied, we may not expect a common pattern across response categories within the different appraisals. From a cultural source hypothesis, we may expect a unimodal or even a normal distribution of response categories, which implies that widely shared cultural scripts are applied across different experiences to make sense of the world. To explore possible patterns, we presented an extended list of nonordinary experiences that vary widely in the cognitive and sensory modalities and plausible underlying neurocognitive mechanisms to participants from a general population sample. Individuals were asked to appraise one experience using the full list of appraisals described in study 1. Pooling these data across all participants and experiences will allow us to get a general overview of the relative prevalence of these appraisals in the general population.

### Methods

#### Participants.

We employed a cross-sectional design, aiming for an approximately equal gender distribution (50% male, 50% female). Data collection was conducted through an online panel company (Netquest.com). The sample consisted of 5,117 participants and the demographic distribution in terms of age, gender and geographic region broadly aligned with the 2010 census data. However, highly educated individuals were overrepresented in the data compared to the population structure (Table 3).

**Table 3:** Demographic distribution of the study sample.

Variable	Category	N = 5,117	Percent
Age	18-25	266	5.20%
	26-35	1,225	23.94%
	36-45	1,198	23.41%
	46-55	968	18.92%
	56-65	975	19.05%
	65+	485	9.48%
Sex	Male	2,554	49.91%
	Female	2,563	50.09%
Education	Illiterate / Incomplete Primary Education	25	0.49%
	Complete Primary Education / Incomplete Middle School	63	1.23%
	Complete Middle School / Incomplete High School	136	2.66%
	Complete High School / Incomplete College	1,900	37.13%
	Complete College	1,749	34.18%
	Postgraduate	960	18.76%
	Master’s Degree	208	4.06%
	Doctorate	76	1.49%
Region	North	123	2.40%
	Northeast	831	16.24%
	Southeast	3,097	60.52%
	South	794	15.52%
	Central-West	272	5.32%

All participants provided informed consent, as required by the Informed Consent Form (ICF), which was approved by the Research Ethics Committee of the D’Or Institute for Research and Education – IDOR (CAAE: 65573322.6.0000.5249). Individuals who did not provide consent were excluded from the study. During the collection, a total of 10,255 participants opened the link to answer the questionnaire. Of these, 87 refused to participate as indicated by their refusal to sign the consent form. Among the remainder, 1,237 were excluded due to failing attention questions, 3,809 abandoned the questionnaire before full completion, and 5 were excluded due to duplications that indicated data corruption or user input error, compromising response validity. This resulted in a final sample of 5,117 completed questionnaires. We required complete responses in order for participants to advance in the survey. For this reason, we did not have any missing information at item level and no imputations or other missing data treatment was necessary.

### Instruments

The online questionnaire began with the Inventory of Nonordinary Experiences (INOE), comprising 38 experience items: absorption, another self in body, auras, awe (negative), awe (positive), compassion, déjà vu, devotion (objects), devotion (people), diminished self, extrasensory perception (minds), faces, fear, guidance, hopelessness, joy, light, loss, love, lucid dreams, meaning in life, misfortune, near death experiences, objects (animated), out of body experiences, pain, paralysis, past life, places (animated), places (special), pleasure, presence of extraordinary forces, presence of the deceased, psychic vampirism, smells, sounds, touch, visions [4,16,17]. For the full list of items, please see S1 Table in the supporting information.

Items were presented in order of lowest to highest prevalence, based on prior research in the Brazilian population [14]. The presentation began with the lowest frequency item available, and items were removed from the pool once 100 complete responses were collected. Two attention-check questions were implemented between the appraisal items to ensure response quality. If the participant failed to answer the attention-check question correctly, their appraisal response was not considered valid. Although this approach was designed to equalize the number of responses across experiences, some items remained longer in the pool due to a programming error and continued to accumulate responses beyond the intended threshold. As a result, the number of responses per experience varied, with a minimum of 100, a mean of 134 responses, and a maximum of 733 participants who reported having had a given experience.

Participants responded to having each experience with either “Yes” or “No.” If a participant selected “No,” the next experience (the next most frequent item within the item pool) was presented. If “Yes” was selected, the participant was directed to a detailed appraisal questionnaire, which included the validated set of appraisal categories described in study 1 (for the full list, see S3 Table).

The frequency question was presented first. If the participant reported having had the experience only once, subsequent appraisals focused on that single occurrence. If the participant had experienced it more than once, the appraisals referred to the most salient experience.

#### Statistical analysis.

We conducted descriptive analyses to summarize the demographic characteristics of the sample, and the overall prevalence of appraisals associated with nonordinary experiences. For each appraisal variable, we computed the absolute frequency and the average percentage of each response category across the 38 distinct nonordinary experiences. To describe the distribution of appraisal responses, we calculated summary statistics including the mean, standard deviation, median, minimum and maximum values of the percentage frequencies.

To further assess distributional patterns, we applied three statistical tests. Silverman’s test for multimodality, implemented using the *multimode* package [93], was used to test whether a distribution contains more than one mode. Hartigan’s dip test for unimodality, from the *diptest* package [94], was used to assess whether a distribution deviates from unimodality. The Shapiro-Wilk test, available in the base *stats* package, was employed to evaluate the normality of each distribution [95]. When multiple modes were detected, the two most prominent peaks were extracted based on kernel density estimates. All descriptive and inferential results were compiled into a summary table to facilitate the interpretation of appraisal patterns at the population level.

All analyses were conducted in R version 4.3.0 [96]. All data and code is available on the OSF (https://osf.io/3fbx4/).

### Results

Table 4 presents the basic statistical results concerning the prevalence of nonordinary experience (NOE) appraisals among the 5,117 respondents. The table includes absolute and mean frequencies of appraisals across all NOEs, the standard deviation of these means, as well as the maximum and minimum percentages for each response. We report the results by broad appraisal groups (see Table 4). The results presented illustrate the distribution of choices across each appraisal category.

**Table 4:** Prevalence statistics of nonordinary experience appraisals.

Group	Appraisal	Category	Percent mean frequency	σ of mean frequency	Max. percent frequency	Min. percent frequency	Absolute frequency
**Situational**	Mental state	Awake and alert	60.13%	18.33%	85.05%	1.96%	3,458
		Under the influence of substances	3.06%	3.04%	15.38%	0.00%	184
		Suffering from a mental or physical illness	2.92%	2.86%	13.08%	0.00%	167
		Falling asleep, waking up, or exhausted	9.44%	5.84%	26.45%	1.74%	528
		Sleeping (dreaming)	17.80%	15.21%	81.05%	3.31%	1,019
		None of the above	6.65%	3.59%	16.94%	1.55%	356
	Context	None of the above	21.24%	9.45%	48.28%	6.77%	1,166
		Listening to music	6.73%	3.90%	15.79%	1.49%	433
		In a religious or spiritual context	10.42%	9.22%	32.33%	1.69%	653
		In a context with a lot of noise, smoke, darkness	2.70%	1.69%	8.06%	0.00%	167
		In a hospital or healthcare setting	5.90%	5.30%	17.21%	0.00%	360
		In an unfamiliar or new place	6.31%	4.98%	25.52%	0.92%	393
		In nature or forest	6.76%	6.59%	32.48%	0.00%	414
		Alone	39.93%	12.95%	70.09%	20.75%	2,267
**Temporal**	Duration	Very quick, almost instantaneous	27.30%	16.86%	74.60%	6.14%	1,372
		A few minutes	36.10%	10.19%	50.93%	14.68%	1,821
		Around an hour	5.40%	3.68%	13.39%	0.00%	299
		More than an hour	5.49%	5.00%	21.74%	0.00%	309
		Days	6.92%	8.61%	31.30%	0.00%	369
		I can’t say	18.80%	9.03%	45.71%	4.55%	947
	Frequency	Once	30.34%	14.25%	62.28%	9.24%	1,500
		2 - 3 times	37.96%	5.74%	47.17%	26.32%	1,993
		4 - 5 times	11.65%	4.39%	22.61%	0.87%	610
		6 - 10 times	3.73%	2.60%	11.40%	0.00%	198
		More than 10 times	16.32%	10.37%	41.85%	0.00%	816
	Onset	Childhood	7.32%	4.32%	24.51%	0.91%	357
		Adolescence/youth	20.76%	5.56%	35.45%	12.62%	1,072
		Adulthood	66.75%	7.38%	81.25%	46.08%	3,429
		I don’t remember	5.17%	4.04%	19.42%	0.00%	259
**Reasoning/belief**	Agent	God or gods	16.35%	7.84%	32.88%	4.55%	923
		Holy spirit	10,87	6.91%	35.58%	1.75%	599
		Angel(s) or saint(s)	3,79	2.98%	12.39%	0.00%	190
		Demon(s)	1,06	1.68%	7.41%	0.00%	52
		E.g., entity, *orixá*, etc.	5,06	4.40%	19.42%	0.00%	251
		Unknown beings, spirits, or forces	7,13	5.45%	20.91%	0.00%	361
		Ets	0,6	2.28%	14.00%	0.00%	28
		Energy(ies)	9,44	6.14%	30.83%	0.87%	466
		Spell, witchcraft, evil eye, or ill intention	1,21	1.67%	6.96%	0.00%	62
		Pray or good intentions sent	3,14	1.91%	8.74%	0.00%	159
		I don’t believe that ‘someone’ caused it	36,45	17.03%	72.82%	8.57%	1.776
		Others	4,89	3.05%	11.40%	0.00%	250
	Reason	To give me a sign or message	28.05%	12.13%	59.22%	8.15%	1,471
		To reward or punish me for my actions	3.57%	2.43%	9.17%	0.00%	203
		Because of my fate or life purpose/mission	16.13%	8.83%	37.27%	1.63%	859
		Because of my abilities, responsibilities, or roles	11.13%	6.28%	29.89%	2.63%	579
		None of the above (it might have been by chance)	41.12%	15.98%	77.67%	17.12%	2,005
	S/R (religion)	Yes	45.61%	18.45%	76.99%	12.62%	2,368
		No	32.55%	17.18%	66.30%	4.50%	1,655
		I don’t know	21.85%	6.23%	35.32%	12.39%	1,094
	Science	Science can or will be able to explain it	48.49%	14.98%	82.61%	22.52%	2,509
		No, something else is involved	51.51%	14.98%	77.48%	17.39%	2,608
**Effect/impact**	Effect moment	Facilitated what i was doing	31.69%	19.31%	73.87%	8.33%	1,8
		Hindered what i was doing	18.60%	16.86%	60.87%	1.82%	878
		Had no effect or interference	49.70%	17.54%	83.57%	20.72%	2,439
	Distress	Great joy or complete well-being	26.12%	19.77%	68.38%	2.63%	1,524
		Some joy or well-being	21.70%	11.29%	44.66%	2.78%	1,095
		Neutral	27.66%	18.89%	66.96%	4.41%	1,369
		Some suffering or discomfort	17.10%	14.34%	48.70%	1.94%	795
		Great suffering or discomfort	7.43%	12.63%	54.39%	0.00%	334
	Life effect	Very positive effect	33.32%	20.63%	71.15%	7.89%	1,879
		Somewhat positive effect	17.61%	6.19%	32.69%	6.48%	904
		Neutral or no effect	34.20%	19.52%	75.73%	8.82%	1,655
		Somewhat negative effect	10.24%	10.37%	38.26%	0.00%	470
		Very negative effect	4.64%	6.44%	31.58%	0.00%	209
	Impact	Little or no impact	30.06%	20.01%	75.54%	4.39%	1,478
		Some impact	39.99%	9.53%	60.87%	21.20%	2,009
		Significant impact	29.95%	17.76%	69.30%	3.26%	1,63
	Community	Positively	32.18%	17.18%	63.24%	9.57%	1,797
		Neutrally	57.76%	14.56%	84.29%	33.09%	2,854
		Negatively	10.06%	9.97%	43.86%	0.91%	466
**Agency/control**	Control	Had total control	29.95%	10.84%	54.81%	11.11%	1,597
		Had little control	25.18%	7.56%	36.52%	9.71%	1,306
		Had no control	24.25%	11.46%	50.43%	6.73%	1,195
		Did not try to control it	20.63%	9.39%	40.38%	6.09%	1,019
	Induction	Tried to induce it	22.65%	12.46%	51.43%	6.14%	1,274
		Tried to avoid the experience	18.09%	12.84%	53.04%	1.94%	876
		Neither tried to avoid nor induce	59.26%	13.01%	86.41%	31.67%	2,967

The percentages for ‘Mental state’ and ‘Context’ do add up to more than 100% because participants could select more than one option.

#### Appraisals regarding situational variables.

Regarding the context, the most prevalent scenario reported was being “Alone,” with an absolute frequency of 2,281, being the average choice for 38.56% of participants across all NOEs. This response was normally distributed across all experience items. The next most common context was a “Religious or spiritual” setting, which was reported by an average of 10% of reports, while the “None of the above” option had an average frequency of 18.35%. All other responses were less frequent (below 10%) and showed non-normal distributions. The responses that showed non-normal distributions and for which mean and median deviated by more than 1% from each other were the “none of the above” response (2.35%), “religious or spiritual context” (4.37%), “hospital or health care setting” (2.48%), and “in nature or a forest” (1.70%).

For the appraisal of mental state during the experience, the most prevalent state reported was being “Awake and alert,” with an absolute frequency of 3,414 responses and an average of 56.6% of participants. When considering altered states of consciousness, the two main items reported were “Sleeping (dreaming)” (18.24%) and “Falling asleep, waking up or exhausted” (10.69%). Altered states related to being “Under the influence of substances” (3.06%) or “Suffering from a mental or physical condition” (2.92%) only accounted for a small fraction of responses (total number of 351 responses combined). All appraisal items displayed non-normality with a substantial positive skewness – except for the “Awake and alert” response, which exhibited negative skewness. The overall discrepancy between mean and median was minor, with the largest difference being observed for “awake and alert” (−6.55%).

#### Temporal appraisals.

Our data on the perceived duration suggested that on average 63.10% of participants’ experiences lasted only up to a few minutes, with 27.30% being “Very quick, almost instantaneous.” Another sizable portion of participants reported not being able to recall the duration of the experience (18.80%). Half of the response categories- i.e., “Very quick,” “More than an hour” and “Days” presented non-normal distributions, while the remaining three were not significantly different from normality. Both the response categories of “very quick” and “days” showed discrepancies between mean and median above 4%.

Regarding frequency of experiences, the majority of individuals reported having had the respective experience “2–3 times” with 37.96% of participants and an absolute number of 2,205 responses. The second most common frequency was “once only,” with a total of 30.34% participants reporting having had an experience once only. The “More than 10 times” option was also reported relatively frequently with 16.32% of participants on average across experiences. The least frequent item was the intermediate frequency of “6–10 times,” with an average of only 3.73% of responses, which was also the only frequency with a non-normal distribution. The discrepancy between mean and median was nevertheless minor (0.50%).

Most participants reported having had the experience during adulthood, with an average of 66.75% across all NOEs and an absolute response of 3,697. On average, 20.76% of participants reported having the experience during their adolescence or youth and only 7.32% during childhood. Importantly, 5.17% of participants reported not remembering when the experience took place in their lives. Both the “childhood” and “no memory” response options showed significant non-normality. The discrepancy between mean and median was below 1%.

#### Appraisals regarding reasoning and beliefs.

For appraisals of the causal agent, the most prevalent response was “I don’t believe that ‘someone’ caused it,” with an absolute number of 1,936 and an average of 32.73% of participants reporting this across all NOEs. The second most common responses were: “God or gods” and “Holy Spirit” responses with 15.95% and 11.66%, respectively. The least frequent average prevalence rates were observed for “Angels/Saints,” “Demons,” “Extraterrestrials” and “Spells, Witchcraft, Evil eye, ....” Overall, appraisals for the agent category showed a unimodal distribution (both unimodality tests p > 0.05), with the only exception being “Demons” (Dip’s p = 0.02). It is worth noting that the “Demons’ category was the only one to show a significant result of the Shapiro-Wilk test indicating possible multimodality. However, visual inspection of the Q-Q plot revealed only minor peaks, and no clear multimodal peak pattern was discernible within the low baseline of about 1% of the responses. For all but three appraisals (’known entities, orixás, etc’ 5.06% vs 3.92%; ‘unknown beings’ 7.13% vs 5.26%, ‘energies’ 9.44% vs 6.14%), the mean and median were within 1% range of each other.

The “reason” appraisal showed a similar overall pattern: the most frequent response on average was “None of the above (it might have been by chance),” with an absolute number of 2,238 responses and an average of 37.82% responses across all NOEs. This was nevertheless closely followed by responses indicating “To give me a sign or message.” The least frequent option was “To reward or punish me for my actions.” Three of the items were normally distributed and the other two appraisals (’to reward or punish,’ ‘abilities, responsibilities or roles’) showed only slight deviations from normality even though the Shapiro-Wilks test was statistically significant.

The results regarding the categories related to spiritual/religious and scientific interpretations revealed a notable division in participants’ interpretations of their nonordinary experiences. In the spiritual/religious appraisal category, an average 48.08% of respondents identified their experiences as having a religious or spiritual character, while 30.77% did not associate their experiences with religion or spirituality, and 21.15% expressed uncertainty. For all these response categories across experiences, the results suggested a normal distribution.

For the science appraisal, a similar division was observed: 49.36% of participants (absolute frequency of 2,918) believed that science is or will be able to explain these experiences, while 50.64% disagree (absolute number of 2,997 responses), indicating a belief in an origin of the experience that transcends scientific explanation. A normal distribution was observed for both response categories of the science appraisals.

#### Agency and control appraisals.

Overall, the responses indicated that most participants felt some degree of control, with the “Had total control” and “Had little control” items together representing an average of 55.77% of the responses. On the other hand, 23.72% of participants on average reported having no control over the experiences and 20.51% did not try to control them at all, representing an average of 44.23% combined. All items presented normal distributions except for the “Did not try to control” response, which displayed a non-normal and platykurtic distribution. The difference between mean and median for this response category was 3.01%.

As for the induction category capturing agency, most participants on average reported neither trying to induce nor avoiding the experience (59.26%), and among the remainder of the participants more people tried to induce it (26.28%) than to avoid it (18.59%). The most common response of neither trying to induce nor avoid showed a normal distribution. This distribution was normal, while the other two questions presented non-normal distributions with a pronounced negative skewness. The largest discrepancy between the mean and median was observed for ‘tried to avoid the experience’ (4.33%).

#### Effect and impact appraisals.

For most individuals across all experiences, the effect in the moment of the experience seemed to cause “no effect or interference” (45.51% on average), followed by the perception that it “Facilitated what I was doing” (34.62%), whereas an average of 19.87% of participants stated that the experience “Hindered what I was doing.” Shapiro-Wilk’s results suggested only the “No effect” item was normally distributed across all NOEs. The largest discrepancy between mean and median was observed for the perceived interference or hindrance (6.47%)

The results for the appraisal of joy/distress showed that an average of 49.68% of participants reported feeling some degree of joy or wellbeing during the experiences, while 24.53% perceived them as emotionally neutral and 25.79% felt some degree of suffering or discomfort. The highest absolute frequency for joy/distress appraisals was “Great joy or complete wellbeing,” with 1.643 participants across all NOEs. All items in this category displayed non-normality, except for the “Some joy or wellbeing” response. Compared to other response categories, the average discrepancy between mean and median was 4.44%, indicating that extreme responses shifted the means somewhat higher than the medians (except for the ‘some joy and wellbeing’ response category).

When looking at the long-lasting life effect of the experience, a combined 50.93% of participants on average perceived them as mildly or strongly positive, while 31.01% reported feeling neutral or no effects, and 14.88% felt mild to strong negative effects. The highest absolute frequency for life effect appraisals was “very positive effect,” with 1.987 participants reporting this very positive effect across all NOEs. Similarly to the previous category, all items showed non-normal distributions, except for the “Somewhat positive effect” response. Across all response categories, the discrepancy between mean and median was on average 3.55%. This again suggests that some extreme responses for some experiences shifted the mean response.

Concerning the overall impact of the experience on individuals’ lives, the most common response was that the experience had “Some impact” on average across all items (39.99%), with an absolute frequency of 2,400 responses. The remaining 60% of average prevalence was equally distributed between the two other options “Significant impact” and “Little to no impact.” Both the ‘some impact’ and a ‘positive impact’ response were normally distributed across all experiences, but not the ‘little or no impact’ response. The discrepancy between mean and median for this response category was 6.65%.

Finally, for the community appraisal, most participants indicated that significant others in their family or community evaluated the experience neutrally with an absolute frequency of 3,296 responses and an average of 57.76% across all NOEs. Only a small group of 10.06% perceived the experience as being negatively evaluated. Additionally, an average of 32.18% participants reported a perceived positive evaluation by their communities. The neutral option was normally distributed across all experiences, but not the positive and negative community appraisals. Both responses showed percentage shifts around 4%, indicating that extreme responses towards the positive or negative end, respectively shifted the means towards more extreme values.

### Discussion

Our analysis extends a range of appraisals and contextual features that are of importance to understand nonordinary experiences, that is experiences that stand out to individuals compared to other day-to-day experiences. Across a variety of experiences, ranging from strong positive and negative emotional experiences to perceptual alterations and changes in the perception of one’s self that are common in mediumistic and spirit-possession contexts, we have shown that most context features and appraisals follow a unimodal distribution that approximates a normal distribution. Given the diversity of the experiences and the appraisals, this distribution has some interesting implications. For the situational context information, it suggests that experiences that stand out to individuals occur when they are awake and alert, suggesting that such experiences are not dependent on altered states of consciousness. For the distribution of appraisals, we observed patterns that often did not deviate from a normal distribution peak, which could be seen as most closely aligned with a cultural source hypothesis. There is certainly variability in the way people make sense of specific experiences, but there appears to be modal forms of both the context in which they occur and their associated interpretation processes, suggesting that individuals rely on the same set of appraisals when making sense of a wide range of salient experiences that deviate from the ordinary flow of consciousness. We now elaborate more on some of these overall patterns and draw out some implications for the study of human consciousness, nonordinary experiences and mental health.

#### Situational and temporal features.

The previously published set of follow-up questions had one set of questions focusing on the mental state of individuals at the time of the experience. Most individuals in the original validation study [4] suggested that the mental states included were insufficient. We added an alert and awake category, which turned out to be the most frequently selected option in our sample. This is of theoretical interest for researchers of altered states of consciousness that often focus on nonordinary experiences in the context of psychoactive substances. The prevalence for the reported use of psychoactive substances was below 5%. Even though the distribution was non-normal, the differences from mean to median were minor and the maximal percentage for any experience was about 15%. On the other hand, awake and alert was the most frequent response on average, but with some variability across experiences. This could be expected as we had specific dream experiences in our study, which explains the non-normality. A further interesting observation is the low response numbers for suffering from mental or physical illness overall. Even though we had a number of experiences that are often associated with clinical conditions and health problems (e.g., misfortune, hopelessness, near death), the maximum frequency of having suffered a clinical condition or health problem for any experience was 13%. Therefore, nonordinary experiences were *not* very likely to occur in the context of either psychoactive substances or illness. This suggests that nonordinary experiences more broadly are part of the waking cycle of humans, which has implications for the understanding of both conscious experience and mental health.

Our study expanded the evaluation of situational and temporal features of experiences overall. We added new probing questions around the context, which may be important for future testing of theories of consciousness that make specific predictions on situational features such as predictive coding models [56,88,97]. Considering the predictive coding conceptualization of perceptual phenomena as “controlled hallucinations,” where top-down probabilistic predictions are regulated by bottom-up sensory input [98–100], these contextual questions may be specially relevant for understanding nonordinary experiences of perceptual nature (e.g., faces, smells, sounds, touch, presence, visions). Contrary to what could have been expected by the same theoretical framework and some experimental findings [101], only a small fraction of participants (3.8% on average) reported having nonordinary experiences in contexts of perceptual uncertainty, such as sceneries with dim light, smoke or noise. On the other hand, somewhat in accordance with predictive coding and source-monitoring theory [102,103], the context of being alone was the most prevalent and displayed a unimodal normal distribution across all NOEs. This can be explained by a distinctive cognitive configuration generated by the context of solitude, in which: a) attention to internal stimuli may be heightened, thereby rendering aberrant signals more salient, and b) the absence of other people restricts the possibility of reality testing through interpersonal validation. Such scenarios allow for top-down processes – e.g., memories, beliefs, expectations – to exert greater influence over perceptual inference than external sensory information, while the lack of consensual verification reduces the perceiver’s ability to discriminate between internally and externally generated experiences, increasing the likelihood of misattributing stimuli to external sources [100–103].

We added new questions on the temporal characteristics of experiences that help understand their onset, duration and frequency. These aspects could be crucial for unveiling how psychological factors such as personality traits and cognitive functioning [58,59] might predict nonordinary experiences (e.g., in terms of frequency). Regarding onset, it could be expected that a significant amount of nonordinary reports remounted to childhood years, considering the higher tendency for magical explanations [40] and underdeveloped domain-specific knowledge [104] during this stage of life. This hypothesis was, however, contradicted by our findings. The emergent pattern of temporal dynamics showed that nonordinary experiences occur primarily when individuals are adults, are relatively rare in the lifetime of an individual (less than three times), are momentarily fleeting and of short duration. Many of these temporal responses are normally distributed across all experiences, suggesting a relative communality of this pattern for events that are cognized as “standing out.”

However, dominant patterns of both contextual and temporal features (i.e., being alone and very low frequency during lifetime) are accompanied by less frequent patterns that point to a heterogeneity of processes that may elicit nonordinary states and experiences. For example, about 10% of respondents on average reported having the experience in religious or spiritual contexts, while 16% of participants reported having had the experience more than 10 times. This highlights that nonordinary experiences can be recurring for some individuals [2] and may be induced within collective contexts. Furthermore, it is worth highlighting that the “I can’t say” option for the duration appraisal displayed an average frequency of 18.8% throughout all NOEs, which means that close to one in five participants did not remember the duration of the experience they were reporting. This aspect of temporal information retrieval is of special relevance to understanding how some nonordinary experiences might be associated with altered states of time perception, which could be sometimes attributed to strong emotional responses and physiological arousal [105,106] but also dissociative-like states of consciousness [107].

#### Reasoning and belief appraisals.

Our work extended some of the response categories in the original appraisal section, in particular differentiating a wider set of possible agents that may be seen as causing the experiences and additional reasons for the experiences. A first observation is that a significant proportion of individuals believe that no specific agent causes the experience and that it may have been due to chance. Yet, this pattern betrays some more complex attributions in a highly religious context. The extensions of the agent responses into a total of 11 distinct options helped to describe a more nuanced picture of appraisals in this category. Combining various religious and spiritual agents within a Brazilian context (God(s), holy spirit, angels, orixás, unknown spirits, energies), around 50% of the experiences on average were attributed to a supernatural cause, which dovetails with the appraisal that the experiences involve more than what science can explain and the experience itself may be of spiritual or religious character. These responses in combination are around the 50% mark for the different responses. This clearly suggests that the collective religious context shapes the appraisal processes across a wide range of experiences. In other words, the religious context, independent of the individual belief systems, offers a powerful schema to explain a variety of experiences within an overarching supernatural belief system.

The nearly even split across the religious vs non-religious appraisals also reflects the diversity of opinions in a modern society. One of the interesting questions for further research is a more careful examination of appraisal patterns across individuals with different religious and spiritual affiliations for specific experiences. We did not have information on the religious affiliation of the participants but hope to explore these patterns in future studies. What might be particularly interesting to study is whether some types of experiences are more prone to religious or spiritual explanations independent of the professed religious affiliation of an individual. Such a project may shed new light on the importance of implicitly shared belief systems without explicitly believing.

Previous works regarding cultural cognitive frameworks such as the distributed cognition approach [108,109] and the cultural-cognitive model [110] have already emphasized that cognitive processes extend beyond the individual into socio-cultural systems, where culture actively shapes information processing and meaning-making. A vast literature of cross-cultural studies, as highlighted by Kastanakis and Voyer [111], point to differences in perception of the self and others, emotions, aesthetics and sensorial perception (visual, auditory and olfactory) in different cultural settings, while our study strongly suggests that appraisal processes of nonordinary experiences would also be implicated.

#### Mental health implications and perceived agency.

One major question in the wider literature on nonordinary experiences is the impact on mental health and possible clinical implications [15]. As noted in the introduction, current scales often conflate the experience with an evaluation of the clinical or spiritual nature of the experience, which makes it difficult to clearly separate out the phenomenological characteristics of the experience from the health implications. Examining the mental health relevant appraisals across this wide range of experiences, the first impression is that nonordinary experiences have at least some impact on individuals, but that these effects are not detrimental at the moment when they occur, do have little effect or even moderate to strong positive longer term effects and are generally perceived as neutral or positive by others in the community. This observation stands in stark relief to a larger literature in psychiatry and clinical contexts that has focused on the negative aspects of experiences that imply altered states of consciousness or alterations in the cognitive or perceptual system. However, this is not to say that experiences are always positive. A substantive number of responses suggested that the experience had a negative impact on the individual at the moment (with an average of 18%, reaching up to a maximum of 60%) and resulted in at least some suffering or discomfort (over 25%, with a maximum reaching over 50%). Given the range of experiences that are included, including hopelessness, misfortune and near death, suffering is to be expected in these reports. An important avenue for future studies is to probe more carefully not only what types of experiences are appraised negatively, but also what maintains this distress over time. In this regard, cognitive theories suggest that one key mechanism is the interaction between a negative appraisal and the adoption of safety-seeking behaviours, which can create a maintaining cycle of distress even from an initially benign experience [112]. Understanding what helps individuals avoid or break this cycle is crucial for explaining how most nonordinary experiences, even negative ones, are ultimately integrated in a way that leads to positive long-term effects [46].

#### Limitations.

Several limitations should be noted. First, only a single experience was reported per participant, which precludes further probing of appraisals and contextual features across different experiences within and across individuals. This limits insights into the variability and stability of appraisal processes within individuals. Second, our focus was restricted to closed-ended responses, which does not capture the qualitative nature of the subjective experiences. Future research exploring the qualitative, narrative features of the reported experiences could provide richer context and deeper understanding of the subjective nature. Third, while the sample was nearly representative in several respects in relation to the last published national census information, it was more educated than the general population. This may constrain the generalizability of findings, particularly to individuals with lower literacy levels who are often overlooked in behavioral research yet may exhibit distinct cognitive and appraisal patterns and thereby offer new insights into the human cognitive system. Finally, although the study was conducted within a single national context, our setting encompasses substantial religious, ethnic, and cultural diversity, while all participants were embedded within a shared linguistic, legal, sociocultural and historical national context. Our sampling constitutes a meaningful advance over research relying primarily on student samples in atypical university environments which do not represent the lived reality of the majority of humankind, and future studies should further extend this work to diverse cultural and socioeconomic settings to enhance the ecological validity of our understanding of nonordinary experiences.

## Summary

Our study aimed to explore the prevalence of contextual features and appraisals of a wide range of nonordinary experiences (NOEs) in the Brazilian population. Our work builds on and extends previous research that has tried to separate phenomenological features of experience from their appraisals [4]. Focusing specifically on the contextual features and appraisals, we first validated an extended list and then studied the prevalence of these features and appraisals in a nearly representative sample of 5,117 participants. Although primarily descriptive, a careful examination of phenomena that have been largely overlooked in the academic literature, yet are globally relevant, plays a crucial role in generating new hypotheses and research opportunities. Our mixed-methods approach with large samples from the general population offer new insights into how, when and where individuals have experience that stand out compared to day-to-day experience and how they then interpret and make meaning of these experiences. These patterns offer intriguing new insights into ongoing discussions of the nature and characteristics of human consciousness.

## Supporting information

S1 Table Inventory of nonordinary experiences (English). Displays the 38 validated experience items used in study 2.
(DOCX)

S2 Table Comparative analysis of the original and extended appraisal questions. Compares phrasing and response options between the original and extended versions of the original appraisal items from Taves et al [4].
(DOCX)

S3 Table Complete list of appraisals and contextual features. Complete list of all appraisal and contextual feature questions, including full response options for each item.
(DOCX)

S1 Text Theoretical foundations of item extensions and additions. This supporting text provides further elaboration on the scientific and empirical literature that contextualized the development and elaboration of the appraisal and contextual feature items.
(DOCX)

S1 File CEP-IRB approval documents. (ZIP)
