# Supplementary material for: Making sense of the extraordinary – An investigation of the content and prevalence of appraisals of nonordinary experiences
Source: PLoS One. 2026 Aug 5;21(8):e0353126. doi: 10.1371/journal.pone.0353126 (PMC13440842; doi:10.1371/journal.pone.0353126)
Supplement: Supplementary file 1 [file pone.0353126.s001.docx]

| **S1 Table.** Inventory of Nonordinary Experiences: Items used in study 2 | |  |
| --- | --- | --- |
| **Nonordinary experience** | **Item phrasing (English)** | **Item phrasing (Portuguese)** |
| Absorption | I have had an experience in which I was completely absorbed in what I was doing and unaware of the passage of time. | Eu tive uma experiência em que estava completamente imerso no que estava fazendo e não percebi a passagem do tempo. |
| Another Self in Body **†** | I have had an experience in which it seemed like there was another self or being in my body. | Eu tive uma experiência em que meu corpo já não era só meu, como se outro ser dentro de mim comandasse minhas ações e sentimentos. |
| Auras **†** | I have seen an aura or halo of light surrounding a person, for which there seemed to be no ordinary source. | Eu vi uma aura ou halo de luz envolvendo uma pessoa sem que houvesse uma fonte externa, ou causa aparente para isso. |
| Awe (Threatening) **†** | I had an experience that, despite being frightening, filled me with profound wonder and fascination. | Eu tive uma experiência que, apesar de assustadora, me encheu de um profundo deslumbramento e fascínio. |
| Awe (Positive) | I have had an experience of awe, wonder, or amazement that stood out from all other such experiences. | Eu tive uma experiência em que fiquei fascinado e maravilhado ao ver, sentir ou perceber algo grandioso de uma forma nova que desafiou como eu pensava até então. |
| Compassion | I can recall a specific experience in which I felt compassion for the suffering of others (human or nonhuman) that stood out from all other such experiences. | Eu tive uma experiência específica em que senti compaixão pelo sofrimento de outros (humanos ou não) que se destacou de todas as outras experiências do tipo que eu já tive. |
| Déjà Vu | I can recall one specific experience in which a new situation felt so familiar to me that it seemed as if I had been there before. | Eu me lembro de uma experiência específica em que tive a sensação de já ter vivido ou visto aquilo antes, como um “déjà vu”. |
| Devotion (Objects) | I have felt devotion or intense attachment toward an irreplaceable object or image. | Eu já tive um sentimento de ligação forte e profundo com uma imagem ou objeto que era único e insubstituível para mim. |
| Devotion (People) | I have felt intense devotion toward a leader or influential person. | Eu já senti uma devoção intensa por um líder ou uma pessoa influente. |
| Diminished Self | I have felt small or insignificant relative to something vast or powerful. | Eu já me senti pequeno ou insignificante em relação a algo vasto ou poderoso. |
| Extrasensory Perception (Minds) | I have had the experience of thinking of a person I haven’t heard from in ages, and later in the day they contacted me. | Eu já tive a experiência de pensar em uma pessoa de quem não ouço falar há anos, e mais tarde no mesmo dia a pessoa entrou em contato comigo. |
| Faces | I have seen what seemed like a face in a natural or human-made object. | Eu vi o que parecia ser um rosto em um objeto da natureza ou feito pelo ser humano. |
| Fear | I have had an experience of fear or horror that stood out from all other such experiences. | Eu tive uma experiência de medo ou horror que se destacou de todas as outras experiências do tipo que eu já tive. |
| Guidance | I have had an experience of being guided or influenced by what seemed to be a nonordinary power or being. | Eu tive uma experiência em que parecia que havia alguma força, energia ou ser extraordinário guiando ou influenciando minhas decisões. |
| Hopelessness | I have had a feeling of hopelessness that stood out from all other such feelings. | Eu tive uma experiência em que senti uma falta de esperança que se destacou de todos as outras experiências do tipo que eu já tive. |
| Joy | I have had an experience of joy, ecstasy, or bliss that stood out from all other such experiences | Eu tive uma experiência de alegria, êxtase ou satisfação plena de uma forma que se destacou de todas as outras experiências do tipo que eu já tive. |
| Light | I have perceived light or lights for which there seemed to be no ordinary physical source. | Eu percebi uma luz ou luzes para as quais parecia não haver uma explicação usual. |
| Loss | I have had an experience of loss (of any kind) that stood out from all other such experiences. | Eu tive uma experiência de perder algo ou alguém que se destacou de todas as outras experiências do tipo que eu já tive. |
| Love | I have had an experience of love that stood out from all other such experiences. | Eu já senti amor de uma forma que se destacou de todas as outras experiências do tipo que eu já tive. |
| Lucid Dream | I have had the experience of being aware that I was dreaming while asleep. | Durante um sonho, eu já tive uma experiência em que sabia que eu estava sonhando, apesar de ainda não ter acordado. |
| Meaning in Life | I have had an experience in which the meaning and purpose of my life suddenly seemed clear. | Eu tive uma experiência na qual o sentido e propósito da minha vida de repente pareceram claros. |
| Misfortune | I have had an experience of misfortune that stood out from all other such experiences. | Eu tive uma experiência de desgraça ou tragédia que se destacou de todas as outras experiências do tipo que eu já tive. |
| Near Death Experience | I have had an experience in which I felt as if I was about to die. | Eu já tive uma experiência em que estive prestes a morrer. |
| Objects (Animated) | I have seen and/or interacted with images, statues, or other physical objects that seemed to contain a nonordinary power or being. | Eu já tive uma experiência em que uma imagem, estátua ou objeto parecia ter algum poder especial, como se uma força ou ser superior estivesse contido ali. |
| Out of Body Experiences | I have had an experience in which it seemed as if I left my physical body. | Passei por uma situação em que eu estava fora do meu corpo físico e pude percebê-lo separado de mim. |
| Pain | I have had an experience of physical pain that stood out from all other such experiences. | Eu tive uma experiência de dor que se destacou de todas as outras experiências do tipo que eu já tive. |
| Paralysis | I have been unable to move or communicate because my body, my limbs, or my voice was paralyzed. | Não consegui me mover ou me comunicar porque meu corpo, meus membros ou minha voz estavam paralisados. |
| Past Life | I have distinct memories that made it seem as if I had lived a past life in a different body. | Eu tenho memórias específicas que me fazem crer que eu posso já ter vivido uma vida anterior, em outro corpo diferente do meu. |
| Places (Animated) | I have experienced a particular place that seemed to contain a nonordinary presence or power. | Eu estive em um lugar específico que parecia conter uma presença, força ou poder extraordinário. |
| Places (Special) | I have felt a sense of deep attachment or connection to a specific place that stood out from all other such places. | Eu tive um sentimento profundo de apego e forte conexão com um determinado lugar que eu jamais senti em qualquer outro local. |
| Pleasure | I have had an experience of pleasure that stood out from all other such experiences. | Eu tive uma experiência prazerosa que se destacou de todas as outras experiências do tipo que já eu tive. |
| Presence (Extraordinary forces) | I have sensed the presence of what seemed to be nonordinary forces or beings. | Eu já senti que seres ou forças extraordinárias estavam ao meu redor (por exemplo, em lugares onde estive, em objetos ou imagens ou a partir da minha própria intuição ou sensação). |
| Presence (Deceased) **†** | I have sensed the presence of, or interacted with, a person who was dead, either directly or with the assistance of another person. | Eu senti a presença ou me comuniquei com uma pessoa falecida, seja diretamente ou com a ajuda de outra pessoa. |
| Psychic Vampirism **†** | I have had the feeling of gaining or losing energy in my body when interacting with certain people, objects, or entities. | Eu já tive a sensação de ganhar ou perder energia no meu corpo ao interagir com certas pessoas, objetos ou entidades. |
| Smells **†** | I have smelled a scent, fragrance, or odor even though nothing around me could have produced it. | Eu tive uma experiência em que senti um cheiro bem específico de alguma coisa, mas não parecia haver nada no ambiente que justificasse essa sensação. |
| Sounds | I have heard a voice when it did not seem like anyone was really there. | Eu já ouvi uma voz quando ninguém parecia estar por perto. |
| Touch | I have felt a physical touch when it did not seem like anyone was really there. | Eu já senti um toque físico quando ninguém parecia estar por perto. |
| Visions **†** | I have had an experience in which I saw objects, people or animals that other people could not see. | Eu tive uma experiência em que vi objetos, pessoas ou animais que outras pessoas não conseguiam ver. |
| *Note.* This is the Brazilian version of the INOE. Some items from the original INOE (US) were not successfully validated, while other new items were added. | | |
| † New validated items in the Brazilian version of INOE (2, 3). | |  |

**References**

1. Taves A, Ihm E, Wolf M, Barlev M, Kinsella M, Vyas M. The Inventory of Nonordinary Experiences (INOE): Evidence of validity in the United States and India. PLOS ONE [Internet]. 2023 July 26[cited 2024 June 18]; 18(7):e0287780. Available from: https://journals.plos.org/plosone/article?id=10.1371/journal.pone.0287780

2. Maraldi E, Fischer R, de Lima Varejão M, Bortolini G, Laport M, Hartle L, et al. Investigating mediumship/possession-relevant experiences: contributions from a feature-based, subject-dependent approach to definition and measurement [Internet]. OSF; 2025 [cited 2025 June 16]. Available from: https://osf.io/br9gy_v1

3. Bortolini, T., Laport, M.C., Bortolini, G.N., Sá, C.A., Fischer, R. & Moll, J. Mapping the Content Landscape of Self-Transcendent Emotional Experiences Using a Mixed-Method Approach. Institute D’Or for Research and Education.; 2025.
