# Supplementary material for: Making sense of the extraordinary – An investigation of the content and prevalence of appraisals of nonordinary experiences
Source: PLoS One. 2026 Aug 5;21(8):e0353126. doi: 10.1371/journal.pone.0353126 (PMC13440842; doi:10.1371/journal.pone.0353126)
Supplement: Supplementary file 2 [file pone.0353126.s002.docx]

**S2 Table.** Comparative analysis of the original and extended appraisal questions.

| **Original follow up questions (Taves et al., 2023)** | **Follow up questions modified to Brazilian context (backtranslated to English)** |
| --- | --- |
| Mental state:  When you had the experience, were you … (Select the most important):   - Using drugs or alcohol - Affected by mental or physical illness - Falling asleep, waking up, or exhausted - Asleep (dreaming) - None of the above | When you had the experience, were you … (Select all options that apply)   - Awake and alert - Under the influence of alcohol or substance(s) - Suffering from a mental or physical illness - Falling asleep, waking up, or exhausted - Sleeping (dreaming) - None of the above |
| Impact:  Overall, how much of an impact has this experience had on your life?   - Little or no impact - Some impact - Major impact | In general, what was the impact of this experience on your life?   - Little or no impact - Some impact - Major impact |
| Life effect:  Overall, has the lasting effect of this experience, on your life or beliefs, been more positive or negative?   - Very positive effect - Somewhat positive effect - Neutral or no effect - Somewhat negative effect - Very negative effect | In general, the long-term effect of this experience on your life and beliefs was:   - Very positive effect - Slightly positive effect - Neutral or no effect - Slightly negative effect - Very negative effect |
| Spiritual/religious:  Do you consider this experience spiritual or religious?   - Yes - No | Do you consider this experience to be spiritual or religious?   - Yes - No - I don’t know |
| Science:  Do you think science can explain how this experience happened?   - Yes, science can or will be able to explain it. - No, something more is involved. | Do you think science can explain how this experience occurred?   - Yes, science can or will be able to explain it. - No, something else is involved. |
| Reason:  Why do you think it happened to you? (Select the closest answer)   - To offer me a sign or message - To reward or punish me for my actions - Due to destiny/fate - None of the above (may include chance/probability) | Why do you think this happened to you? (Choose the closest response)   - To give me a sign or a message - To reward or punish me for my actions - Because of my destiny or life purpose/mission - Because of my abilities, responsibilities, or roles - None of the above (it may have been by chance) |
| Agent:  Who, if anyone, caused you to experience this? (Select the most important.)   - God or gods - Other spiritual beings or forces (including the dead) - None of the above | Who, if anyone, made you experience this? (Select the most important option)   - God or gods - Holy Spirit - Angel(s) or saint(s) - Demon(s) - Ancestors or other known beings, spirits, or forces (e.g., entity, orixá, etc.) - Unknown beings, spirits, or forces - Extraterrestrials - Energy/energies  One or more known or unknown individuals (someone who cast a spell, hex, evil eye, or had ill intentions towards you) - Someone who prayed for you or sent you good intentions - Other(s) - I don’t believe “someone” made me have this experience |

*Note.* Significant modifications are highlighted in red.
