# Supplementary material for: Making sense of the extraordinary – An investigation of the content and prevalence of appraisals of nonordinary experiences
Source: PLoS One. 2026 Aug 5;21(8):e0353126. doi: 10.1371/journal.pone.0353126 (PMC13440842; doi:10.1371/journal.pone.0353126)
Supplement: Supplementary file 3 [file pone.0353126.s003.docx]

**S3 Table.** Complete list of appraisals and contextual features

| **Item** | **Item phrasing** | **Response options** |
| --- | --- | --- |
| **Mental state** | When you had this experience, you were... (select all options that apply) | - Awake and alert - Under the influence of alcohol or substance(s) - Suffering from a mental or physical illness - Falling asleep, waking up, or exhausted - Sleeping (dreaming) - None of the above |
| **Context** | When you had this experience, were you... (select all options that apply) | - Listening to music - In a religious or spiritual context - In a context that made it difficult for you to perceive what was happening (e.g., with a lot of noise, smoke, darkness) - In a hospital or healthcare clinic - In an unknown or new place - In nature or a forest - Alone - None of the above |
| **Frequency** | How many times have you had this experience in your life? | - 1 time - 2 - 3 times - 4 - 5 times - 6 - 10 times - More than 10 times |
| **Duration** | Approximately how long did the experience last? | - Very quick, almost instantaneous - A few minutes - Around an hour - More than an hour - Days - I don't know |
| **Onset** | When did this experience happen in your life? (If you have had it more than once, please indicate the period when it started happening to you.) | - Childhood - Adolescence/Youth - Adulthood - I don't remember |
| **Impact** | In general, what was the impact of this experience on your life? | - Little or no impact - Some impact - Significant impact |
| **Life effect** | In general, the long-term effect of this experience on your life and beliefs was: | - Very positive effect - Slightly positive effect - Neutral or no effect - Slightly negative effect - Very negative effect |
| **Effect moment** | What was the effect of this experience when you had it? | - It facilitated, helped, or complemented what I was doing - It disturbed, interfered with, or interrupted what I was doing - It had no effect or interference |
| **Joy/Distress** | How much would you say this experience caused you something positive (joy/wellbeing) or negative (suffering/discomfort)? | - A lot of joy or complete wellbeing - Some joy or wellbeing - Neutral, it didn't cause me anything - Some suffering or discomfort - A lot of suffering or discomfort |
| **Control** | To what extent were you able to control this experience? | - I had complete control over it - I had little control over it - I had no control over it - I did not attempt to control it |
| **Community** | In general, how would your friends, people in your community, or your family evaluate the fact that you have had this experience? | - Positively - Neutral - Negatively |
| **Induction** | After experiencing this for the first time, did you try to have or induce this experience again? | - Yes, I tried - No, I tried to avoid the experience - No, I neither tried to avoid nor induce it |
| **Spiritual/Religious** | Do you consider this experience to be spiritual or religious? | - Yes - No - I don't know |
| **Science** | Do you think science can explain how this experience occurred? | - Yes, science can or will be able to explain it - No, something else is involved |
| **Reason** | Why do you think this happened to you? (Choose the closest response) | - To give me a sign or a message - To reward or punish me for my actions - Because of my destiny or life purpose/mission - Because of my abilities, responsibilities, or roles - None of the above (it may have been by chance) |
| **Agent** | Who, if anyone, made you experience this? (Select the most important option) | - God or gods - Holy Spirit - Angel(s) or saint(s) - Demon(s) - Ancestors or other known beings, spirits, or forces (e.g., entity, orixá, etc.) - Unknown beings, spirits, or forces - Extraterrestrials - Energy/energies - One or more known or unknown individuals (someone who cast a spell, hex, evil eye, or had ill intentions towards you) - Someone who prayed for you or sent you good intentions - Other(s) - I don't believe "someone" made me have this experience - If "other(s)": Who or what? |
|  |  |  |
