# Supplementary material for: Making sense of the extraordinary – An investigation of the content and prevalence of appraisals of nonordinary experiences
Source: PLoS One. 2026 Aug 5;21(8):e0353126. doi: 10.1371/journal.pone.0353126 (PMC13440842; doi:10.1371/journal.pone.0353126)
Supplement: Supplementary file 4 [file pone.0353126.s004.docx]

**S1 Text.** Theoretical foundations of item extensions and additions.

**Situational context of the Experiences**

The mental state questions considered a number of possible psychological states that may influence the perception of these experiences. We added the additional option "awake and alert" for mental states, in order to differentiate between normal waking conditions and other unmentioned forms of altered mental states (*e.g. hypnosis, trance),* previously condensed under the single option "none of the above''. A specific "awake and alert" option is relevant when considering mind-body practices, such as yoga and meditation, as a popular way of eliciting nonordinary experiences whilst at the same time associated with a state of full alertness and awareness (1). Is it also worth noting that some popular traditions in Brazil, such as Kardecism, support the idea of mediunic experiences that take place during conscious states and minor day-to-day activities (2).

Furthermore, we added a number of questions that focused more on the context and the motivations of individuals. Some of these questions might be relevant for some of the other sections listed above. First, we asked more specific questions on the activities that the individual was involved in prior to and during the experience. This goes beyond the mental state questions from the original instrument and explores whether features like locations, people and objects influence the way we appraise, remember and report events (3). It is known that the brain recurs to schemas and expectations to access incoming information, a mental process mentioned in literature as "the working model" (4). In this model, the relation between the context, body sensations and previous memories serves not just so we can identify what is happening, but also to understand why it is happening and how we are affected by the event itself (5).

Our choice of appraisals was guided by previous work that has emphasized the role of music (6–8) and religious, spiritual or ritualistic contexts for the experience of nonordinary experiences, including strong emotional reactions (9). We added a question on nature considering that many ritualistic practices historically have taken place in natural environments, which have been associated with heightened sensory awareness (10). Natural environments also seem to be commonly related to states of inner peace, creativity, self-discovery, relaxation and a sense of ineffability, which corroborate a possible predisposition of the natural setting to spiritual experiences (11–13). Based on predictive coding models of human cognition, we added options on contexts that make perception more challenging (noise, darkness, smoke) as well as novel or unknown contexts, which may result in stronger top-down signals that lead to altered perceptions of reality, including illusions and possibly hallucinations (14–17). The predictive and generative functions of the brain are constantly recruited in order to, based on previous experience, optimize sensory processing (18), but conditions where sensory input is low or ambiguous may increase the discrepancies between sensory evidence and the brain's expectations. Finally, we added a question on being alone. Being alone limits a person's ability to validate or check any emerging sensory experiences, which may lead to a lack of consensual verification and an inability to cross-validate experiences. Indeed, many mystical experiences - such as communicating with significant others, nature or God - have been reported in the context of solitude (11,19,20).

**Temporal dynamics of the experience**

A first important question on temporal dynamics is the singular or recurrent nature of the experience. We added a question on the number of times that the individual has had the experience. The extensive literature suggests that many nonordinary experiences may have varying frequency in the general population, but for specific individuals these events can be more common or frequent (21). In a Brazilian nationwide study conducted by Monteiro de Barros *et al.* (22), nearly half of the participants reported having spiritual or religious experiences frequently - *i.e.* mystical, mediumistic, psi-related or past life/near-death. It has been observed that members of groups with a parapsychological orientation in which anomalous experiences are more addressed and encouraged tend to report more NOEs than others (23–25).

As human lived experiences have a rich internal relationship with each other due to the cognitive mechanism of predicted processing of events (4,26,27), it is possible to assume that the frequency with which NOEs are experienced by interviewees can impact the perceptual and narrative structure of the experiences themselves. When it comes to the memorization and recall of repeated events, according to the fuzzy trace theory and script theory of memory (28), it is suggested that the general aspects of recurrent events tend to be more strongly remembered than the specific traits of each of its instances. Cognitive schemas, or scripts, might work as a framework for retrieving information of repeated events in a more probabilistic rather than precise manner (*i.e.* what usually happens). In this sense, it is expected that individuals attribute some specific elements of an event to a wrong source when trying to recall details of recurrent experiences - known as accuracy errors - and also change reports of a specific event across retrieval attempts - known as consistency errors (29). Similarly, it has been shown that single, unique emotional experiences can be retrieved and reported with higher accuracy than those that happen repeatedly (30).

Furthermore, another important aspect of the temporal dynamics of an experience is how the frequency of an event might be correlated with certain psychological predictors - such as specific personality and cognitive traits (31,32) - and possibly outcomes - such as mental health and wellbeing (33). For this reason, measuring the frequency of nonordinary experiences is crucial to the understanding of the phenomenon as a whole and its multiple implications.

Second, we included a question on the duration, as this may have an important influence on the retrospective appraisal of the experience. While studies support the idea that the key moments - snapshots - of an event, mainly its peak and end, are more relevant than its duration when evaluating the experience, it is suggested that the time span of the event and the way its key moments are temporally distributed also play a significant - although indirect - role (34), in such a way that longer durations elicit stronger emotional responses to an event's peak and end. Additionally, literature points to a positive correlation between the duration of an experience and the duration of the subsequent emotional response (35).

Third, we included a question about when the experience happened in the life of the individual. From both a developmental and clinical perspective, this is a rather important question to explore as it is clear that the development of event memory is conditioned by the development of knowledge of classes of events. Therefore, we can assume that people are able to appraise events that happened in childhood, but with some particularities (4). On one side, some experiments have shown that children's memories for events are considerably more reliable than what is commonly believed (36). Conversely, adults' autobiographical memories of childhood events are more susceptible to illusion, suggestion, inflation and implantation (37,38), which is of critical importance when designing studies and instruments that aim to measure life events retrospectively.

Beyond this, it is well established within developmental psychology and neuroscience that the impact of strong emotional experiences may differ according to the age of exposure, meaning that events occurring during childhood and adolescence will interact with a brain that's particularly receptive to external influence and also more susceptible to long term neurodevelopmental effects (39) mainly associated with emotional responding (40). When it comes to highly adverse experiences (*i.e.* trauma), literature points to different outcomes in adult mental health and wellbeing depending on the developmental window during which they occur, with some studies highlighting middle childhood as a developmental period that's distinctly sensitive to adverse experiences (41,42). Regarding cognition for new events, it is often suggested that magical explanations are more commonly observed in children, a feature that seems to gradually fade - although not disappear - in adolescent and adult life (43). Finally, major shifts in executive functions (44), emotional processing (45) and domain-specific knowledge (46) that take place throughout the course of development can be responsible for significant age-related differences in the way events are experienced, appraised and remembered.

**Mental health implications of the experience**

We created new effect or impact questions as our project is specifically focused on mental health. Previous studies about the subject reported that participants who mentioned the feelings of preoccupation or doubt about their nonordinary experience evidenced more psychological distress, but the experiences overall showed some beneficial effects for individuals in general. Brett et al. (47) have shown that anomalous states associated with changes in awareness and cognitive function, a greater effort of control and causal attribution to external agents - *i.e.* other people - are predictors of higher psychological distress. On the other hand, the same study has also found that experiences associated with a greater sense of controllability, greater perceived social support and spiritual appraisals are predictors of better psychological outcomes. Nevertheless, more research is still necessary to access this data (33). Furthermore, some earlier works by Osis (48) and Blackmore (49) with individuals who reported Out of Body Experiences (OBEs) suggest that this type of event could have positive impact on well-being, daily functioning and social relationships, although such outcomes seem to vary according to the emotional landscape of the experience and how it is appraised (50). Those were the main premises for including a specific question on the impact of the experience when it occurred, more specific questions on the positive, neutral or negative effects on the person's wellbeing and a specific question on the larger social impact of having had the experience. We consider the last point particularly important because (a) this may be an important appraisal for the functioning and integration of the individual within the larger community, which affects the quality of the experience itself and its outcomes on wellbeing (51,52); b) the social context is of significant importance in more group-oriented cultures, such as Brazil (24).

We also included a question on the perceived control over the experience, as this is an issue that is raised both in the literature when discussing the differentiation of clinical vs spiritual experiences as well as common concern by individuals in different spiritual and religious groups, based on our ethnographic experience. As previously mentioned, some studies have already pointed to an association between the sense of controllability during the experience and its outcomes (47). It is thus relevant to assess the level of attempted control endured in the moment in order to better understand the overall feeling of wellbeing in participants who reported nonordinary experiences (33).

Finally, we included a question about the attempts to possibly induce the experience again or to avoid the experience. This aspect could be related and complement the analysis of the agent question (Q12) as it is known that in folk psychology (53) people tend to give reasons to events or experiences as long as they are intentional, while unintended events have causes - not reasons (54). The induction appraisal may also be a further indication of the possible impact, but focusing more on the motivational states because the motivation may differ, even for positive or negative experiences (e.g., people may want to relive traumatic experiences because of feelings of guilt, may avoid feeling overly positive and happy as this may invoke jealousy or envy by others or it may be deemed immoral).

**Religious, spiritual or scientific explanations and reasoning about the experiences**

We adapted and extended some of the original cause items, extending in particular the choices offered to participants to choose from. For the Spiritual question, we added the "I don't know" option. This was done in order to avoid forcing a directional response by participants who might be unsure about the nature of their experience, which could lead to biased results. When considering the diverse and syncretic characteristics of Brazilian culture, where people may idiosyncratically blend or separate concepts regarding spirituality and religion (55), it is especially relevant to include a response option that is not as clear-cut as a "yes" or "no" in order to capture responses more precisely. Among the participants that accurately understood the question during validation, approximately one in six answered "I don't know".

The categories for the Reason question (Q11) were modified and expanded. The Destiny/Fate category was changed to Destiny or Life purpose and mission, in order to account for the mission-oriented religious attitude and cosmovision (56) and the notion of fulfilling a certain purpose, commonly described in Kardecist, Christian and Afro-Brazilian traditions (57,58) and sometimes associated with the idea of spiritual evolution. Furthermore, the Abilities, responsibilities or roles category was created to address the important aspect of one's individual characteristics and duties within a particular group or community and its influence over the meaning of the experience. It is known that while some cultural contexts might not value extraordinary abilities and discourage them, others will assign meaning and social function to it (59), cultivating such characteristics. There is a multitude of Brazilian traditions in which certain roles are strongly associated with extraordinary phenomena and abilities, such as the mediunic activity in Kardecist spiritism (2) and Afro-Brazilian religions Umbanda and Candomblé (60). Shamanism is also a prominent role in Brazilian culture, specifically within indigenous traditions and neo-shamanic practices (61). It is worth noting that prior expectations - possibly associated with such roles and responsibilities - might also play a part in the way experiences are lived and valued, and it has been suggested that they may shape emotional processing and evoke more intense brain and behavioral responses (17,62,63).

The agent question (Q12) was also significantly expanded to include a wider range of locally relevant agents that may have caused the experience, namely: Holy Spirit; Angels or saints; Demons; Ancestors or other known beings; Unknown beings, spirits or forces; Extraterrestrials; Energy/energies; One or more known or unknown individuals; Someone who prayed for you or sent you good intentions; Others; Nobody.

The addition of the Holy spirit agent was rooted in the fact that this concept is very prominent in various religious traditions across Brazil, and is generally associated with an idea of divine guidance, healing and empowerment, sometimes being responsible for influencing thoughts, emotions and actions (64). The significance of the Holy spirit can be attested through the many religious festivals and celebrations, vastly disseminated through almost all of Brazil's territory, that hold it as a protagonist or key feature (65).

When it comes to religious festivals, especially those formed around Christian iconography, the cultural significance of saints also becomes very apparent. In Brazil, each saint might be popularly associated with specific regions, natural phenomena or professional activities, acting as a patron for one or more of these categories (66). A particular situation regarding the cult of saints in Brazilian culture is the fusion of Christian and Afro-Brazilian iconography, a kind of double-allegiance where saints are associated with their Yorubá counterpart (67). These factors are important to consider when assessing the appraisal of nonordinary events, in the sense that each individual may nurture a unique affect for certain saints, according to their personal qualities, what they represent and how one identifies with them. For this reason, we decided to include saints as a possible agent of nonordinary experiences.

Similarly, the addition of angels in the same category was guided by the cultural significance of these entities, which can be explained by the hegemonic presence of Judeo-Christian symbology in Brazilian culture (68,69). Within the context of Judaism and Christianism, angels are often regarded as messengers from the divine, a kind of bridge between the two worlds, whilst also fulfilling either protective or destructive duties. In this sense, it isn't surprising that some people may narrate encounters with angelic beings, both in normal waking conditions and during dreams, generally associated with a sense of protection and positive outcomes (70).

The Demons category was included in order to capture the prevalent concept and image of evil spirits throughout Brazilian culture (71), which most commonly, although not always, can be understood as equivalent to a "demon". The notion of a human body that's vulnerable to the infiltration of entities in general, including dangerous and malicious ones, is widespread across multiple regions and creeds, but especially relevant in the context of Christian Neo-pentecostal, Catholic and Afro-Brazilian groups. For Brazilian Neo-pentecostals in particular, when a demon invades and takes control of the body, one must go through powerful and cathartic liberation rituals, a kind of exorcism, in order to expel the demonic forces through the power of Christ (72).

As for the Ancestors or other known beings category, we relied on the fact that ancestrality is a central aspect of Afro-Brazilian culture and religion, which conceives a profound interaction between the ancestral realm and the living realm (73). Because of the major cultural influence of the African diaspora in Brazil, this notion is nowadays widespread and not restricted to Afro-Brazilian groups (74). Posteriorly merged with other religious institutions, such as Umbanda, Candomblé and Kardecism, ritualistic practices of Central African origin regarding the possession by ancestor spirits (such as the *Calundú*) have been part of Brazilian culture since colonial times (75). Within Candomblé and Umbanda, one might incorporate well-known entities and divine forces through mediumship (76). In contrast, when it comes to Kardecist spiritism, an important part of the ritual is often attempting to contact errant spirits of deceased family members, loved ones and previously known individuals. It is also possible to identify similar beliefs within other folk traditions, where the spirits of deceased people who may still have pending matters in the material realm, known as *almas penadas,* are left wandering on earth until certain ritualistic acts or works put them to rest (77). In the nationwide study by Monteiro de Barros *et al.* (22), nearly half the sample reported having felt the presence of a dead person. Conversely, we included the Unknown beings, spirits or forces category as a way to encompass a variety of experiences involving agents that do not conform to any previous expectation or known figure. Some examples of such encounters could be found in Kardecism, where sometimes unknown errand spirits or noxious forces may interact with the human world (74).

Across the Brazilian landscape of paranormal experiences, accounts of extraterrestrial encounters are fairly common (78), which motivated the inclusion of an Extraterrestrials category within the agent question. When looking at contemporary esoteric religious and neo-shamanic movements in Brazil - e.g. *Vale do Amanhecer* and groups linked to Ayahuasca rituals - it is expected to stumble across reports of extraterrestrial encounters and related beliefs (79). Some of these movements might not only consider the direct interaction between aliens and humans as real, but will also pursue it (80). Chico Xavier, probably the most iconic medium in Brazilian history and a strong propagator of spiritism, was also a believer in such encounters, specifically the benign interaction between humans and spiritually advanced extraterrestrial beings - an idea that has since been ingrained in spiritist culture and appears as a relevant motif in spiritist and transhumanist literary works (78). Last but not least, it is important to consider the previously studied correlation between general paranormal beliefs and experiences - specially extrasensory perception - and the belief in extraterrestrial encounters (81), and also the common attribution of alien abduction to nonordinary phenomena such as sleep paralysis in multiple cultures, including Brazil (82).

The Energy/energies category was grounded on the fact that the metaphysical notions of energy and subtle energies as a driving force for extraordinary events are highly prevalent in Brazilian culture, mainly within contexts of benign and healing experiences such as the spiritist *passe*, Reiki, Johrei and crystal healing (83,84). In New Age and neo-shamanic environments, the concept of energy might be associated with an appropriation and reinterpretation of quantum mechanics, according to which it is possible for some individuals - i.e. the Shaman - to access and interact with a realm of non-matter (84).

**References**

1.	Franco Corso SJ, O’Malley KY, Subaiya S, Mayall D, Dakwar E. The role of non-ordinary states of consciousness occasioned by mind-body practices in mental health illness. J Affect Disord. 2023 Aug 15;335:166–76.

2.	Maraldi E de O, Machado FR, Zangari W. Importance of a Psychosocial Approach for a Comprehensive Understanding of Mediumship. J Sci Explor 2010 Vol 24 Issue 2 P181. 2010;

3.	Zacks JM, Speer NK, Swallow KM, Braver TS, Reynolds JR. Event perception: A mind-brain perspective. Psychol Bull. 2007;133(2):273–93.

4.	Zacks JM. Event Perception and Memory. Annu Rev Psychol. 2020 Jan 4;71(Volume 71, 2020):165–91.

5.	Taves A, Paloutzian RF. Believing and Appraising in Context: Cognizing Experiences as Events. Front Psychol [Internet]. 2022 May 19 [cited 2025 June 16];13. Available from: https://www.frontiersin.org/journals/psychology/articles/10.3389/fpsyg.2022.913603/full

6.	Rouget G. Music and trance : a theory of the relations between music and possession [Internet]. Chicago : University of Chicago Press; 1985 [cited 2025 June 16]. 430 p. Available from: http://archive.org/details/musictrancetheor0000roug

7.	Herbert R. Everyday Music Listening: Absorption, Dissociation and Trancing. Ashgate Publishing, Ltd.; 2011. 256 p.

8.	Collao LD, Peñalba A. Cognición, experiencia musical y conciencias alteradas: la influencia de las ciencias cognitivas en el estudio sobre la música y el trance. El Oído Pensante [Internet]. 2019 Feb 1 [cited 2025 June 16];7(1). Available from: http://revistascientificas.filo.uba.ar/index.php/oidopensante/article/view/7542

9.	Scherer KR, Schorr A, Johnstone T. (eds.). Appraisal processes in emotion: Theory, methods, research. New York, NY, US: Oxford University Press; 2001. 478 p.

10.	Fredrickson BL, Tugade MM, Waugh CE, Larkin GR. What good are positive emotions in crisis? A prospective study of resilience and emotions following the terrorist attacks on the United States on September 11th, 2001. J Pers Soc Psychol. 2003;84(2):365–76.

11.	Long CR, Averill JR. Solitude: An Exploration of Benefits of Being Alone. J Theory Soc Behav. 2003;33(1):21–44.

12.	Havik G, Elands BHM, van Koppen CSA (Kris). An encounter with one’s deeper self and energy: A phenomenological study among spiritually engaged individuals in the Netherlands. Ecopsychology. 2015;7(2):75–83.

13.	Snell TL, Simmonds JG. Mystical experiences in nature: Comparing outcomes for psychological well-being and environmental behaviour. Arch Für Relig Arch Psychol Relig. 2015;37(2):169–84.

14.	Rao RP, Ballard DH. Predictive coding in the visual cortex: a functional interpretation of some extra-classical receptive-field effects. Nat Neurosci. 1999 Jan;2(1):79–87.

15.	Fletcher PC. Predictive coding and hallucinations: a question of balance: Comment on Powers, Mathys, and Corlett (2017) “Pavlovian conditioning-induced hallucinations result from overweighting of perceptual priors.” Cognit Neuropsychiatry. 2017 Nov 2;22(6):453–60.

16.	Salge JH, Pollmann S, Reeder RR. Anomalous visual experience is linked to perceptual uncertainty and visual imagery vividness. Psychol Res. 2021 July;85(5):1848–65.

17.	Corlett PR, Horga G, Fletcher PC, Alderson-Day B, Schmack K, Powers AR. Hallucinations and Strong Priors. Trends Cogn Sci. 2019 Feb;23(2):114–27.

18.	Shipp S. Neural Elements for Predictive Coding. Front Psychol [Internet]. 2016 Nov 18 [cited 2025 June 16];7. Available from: https://www.frontiersin.org/journals/psychology/articles/10.3389/fpsyg.2016.01792/full

19.	Griffin JD, Fletcher PC. Predictive Processing, Source Monitoring, and Psychosis. Annu Rev Clin Psychol. 2017;13(1):265–89.

20.	Waters F, Allen P, Aleman A, Fernyhough C, Woodward TS, Badcock JC, et al. Auditory Hallucinations in Schizophrenia and Nonschizophrenia Populations: A Review and Integrated Model of Cognitive Mechanisms. Schizophr Bull. 2012 July 1;38(4):683–93.

21.	Ross CA, Joshi S. Paranormal Experiences in the General Population. J Nerv Ment Dis. 1992 June;180(6):357.

22.	Monteiro de Barros MC, Leão FC, Vallada Filho H, Lucchetti G, Moreira-Almeida A, Prieto Peres MF. Prevalence of spiritual and religious experiences in the general population: A Brazilian nationwide study. Transcult Psychiatry. 2022 Apr 6;13634615221088701.

23.	Torres CM. Religiosidade e experiências anômalas no protestantismo brasileiro. 2016.

24.	Maraldi E de O, Krippner S. Cross-cultural research on anomalous experiences: Theoretical issues and methodological challenges. Psychol Conscious Theory Res Pract. 2019;6(3):306–19.

25.	Alvarado CS. Out-of-body experiences. In: Varieties of anomalous experience: Examining the scientific evidence. Washington, DC, US: American Psychological Association; 2000. p. 183–218.

26.	Flanagan JR, Johansson RS. Action plans used in action observation. Nature. 2003 Aug 14;424(6950):769–71.

27.	Eisenberg ML, Zacks JM, Flores S. Dynamic prediction during perception of everyday events. Cogn Res Princ Implic. 2018 Dec 29;3(1):53.

28.	Dilevski N, Paterson HM, Walker SA, van Golde C. Adult memory for specific instances of a repeated event: a preliminary review. Psychiatry Psychol Law. 28(5):711–32.

29.	Rubínová E, Kontogianni F. Sources and destinations of misattributions in recall of instances of repeated events. Mem Cognit. 2023;51(1):188–202.

30.	Theunissen TPM, Meyer T, Memon A, Weinsheimer CC. Adult eyewitness memory for single versus repeated traumatic events. Appl Cogn Psychol. 2017;31(2):164–74.

31.	Irwin HJ, Dagnall N, Drinkwater K. Parapsychological experience as anomalous experience plus paranormal attribution: A questionnaire based on a new approach to measurement. J Parapsychol. 2013;77(1):39–53.

32.	Irwin HJ, Schofield MB, Baker IS. Dissociative tendencies, sensory-processing sensitivity and aberrant salience as predictors of anomalous experiences and paranormal attributions. J Soc Psych Res. 2014;78(917[4]):193–206.

33.	Maraldi E de O, Taves A, Moll J, Hartle L, Moreira-de-Oliveira ME, Bortolini T, et al. Nonordinary Experiences, Well-being and Mental Health: A Systematic Review of the Evidence and Recommendations for Future Research. J Relig Health. 2024 Feb;63(1):410–44.

34.	Weingarten E, Zauberman G, Diehl K. Duration sensitivity of key moments. Cognition. 2021 Sept 1;214:104750.

35.	Verduyn P, Delaveau P, Rotgé JY, Fossati P, Van Mechelen I. Determinants of Emotion Duration and Underlying Psychological and Neural Mechanisms. Emot Rev. 2015 Oct 1;7(4):330–5.

36.	Pezdek K, Roe C. Memory for childhood events: How suggestible is it? Conscious Cogn Int J. 1994;3(3–4):374–87.

37.	Brainerd CJ, Reyna VF, Ceci SJ. Developmental reversals in false memory: A review of data and theory. Psychol Bull. 2008;134(3):343–82.

38.	Brewin CR, Andrews B. Creating Memories for False Autobiographical Events in Childhood: A Systematic Review. Appl Cogn Psychol. 2017;31(1):2–23.

39.	Stevens JS, van Rooij SJH, Jovanovic T. Developmental Contributors to Trauma Response: The Importance of Sensitive Periods, Early Environment, and Sex Differences. In: Vermetten E, Baker DG, Risbrough VB, editors. Behavioral Neurobiology of PTSD [Internet]. Cham: Springer International Publishing; 2018 [cited 2025 June 16]. p. 1–22. Available from: https://doi.org/10.1007/7854_2016_38

40.	Callaghan BL, Tottenham N. The stress acceleration hypothesis: Effects of early-life adversity on emotion circuits and behavior. Curr Opin Behav Sci. 2016;7:76–81.

41.	Dunn EC, Nishimi K, Gomez SH, Powers A, Bradley B. Developmental timing of trauma exposure and emotion dysregulation in adulthood: Are there sensitive periods when trauma is most harmful? J Affect Disord. 2018 Feb;227:869–77.

42.	Toutountzidis D, Gale TM, Irvine K, Sharma S, Laws KR. Childhood trauma and schizotypy in non-clinical samples: A systematic review and meta-analysis. PloS One. 2022;17(6):e0270494.

43.	Woolley JD. Thinking about fantasy: Are children fundamentally different thinkers and believers from adults? Child Dev. 1997;68(6):991–1011.

44.	Luna B, Garver KE, Urban TA, Lazar NA, Sweeney JA. Maturation of cognitive processes from late childhood to adulthood. Child Dev. 2004;75(5):1357–72.

45.	Hoemann K, Xu F, Barrett LF. Emotion words, emotion concepts, and emotional development in children: A constructionist hypothesis. Dev Psychol. 2019 Sept;55(9):1830–49.

46.	Phelps KE, Woolley JD. The form and function of young children’s magical beliefs. Dev Psychol. 1994;30(3):385–94.

47.	Brett C, Heriot-Maitland C, McGuire P, Peters E. Predictors of distress associated with psychotic-like anomalous experiences in clinical and non-clinical populations. Br J Clin Psychol. 2014 June;53(2):213–27.

48.	Osis, K. Insiders’ views of the OBE: a questionnaire survey. WG Roll Ed Res Parapsychol 1978 Metuchen NJ Scarecrow Press. 1979;

49.	Blackmore SJ. A psychological theory of the out-of-body experience. J Parapsychol. 1984;48(3):201–18.

50.	Steffen EM, Wilde DJ, Cooper CE. Affirming the positive in anomalous experiences: A challenge to dominant accounts of reality, life, and death. In: The Routledge international handbook of critical positive psychology. New York, NY, US: Routledge/Taylor & Francis Group; 2018. p. 227–44. (Routledge international handbooks).

51.	Tate AJM. Contributory injustice in psychiatry. J Med Ethics. 2019 Feb;45(2):97–100.

52.	Wantoch S. The Meta of Madness: How the social framing of anomalous experience affects its ontology [Internet] [phd]. University of Sheffield; 2022 [cited 2025 June 16]. Available from: https://etheses.whiterose.ac.uk/id/eprint/32646/

53.	Malle BF. Folk Theory of Mind: Conceptual Foundations of Human Social Cognition. In: The new unconscious. New York, NY, US: Oxford University Press; 2005. p. 225–55. (Oxford series in social cognition and social neuroscience).

54.	Taves A, Asprem E. Experience as event: Event cognition and the study of (religious) experiences. Relig Brain Behav. 2017;7(1):43–62.

55.	Esperandio MRG, Marques LF. The Psychology of Religion in Brazil. Int J Psychol Relig. 2015 Oct 2;25(4):255–71.

56.	Aquino TAA de, Correia APM, Marques ALC, Souza CG de, Assis Freitas HC de, Araújo IF de, et al. Atitude religiosa e sentido da vida: um estudo correlacional. Psicol Ciênc E Prof. 2009;29:228–43.

57.	Cavalcanti MLV de C. Life and death in Kardecist Spiritism. Religião Amp Soc. 2006;1(SE):0–0.

58.	Evangelista DF. Fundando um axé: reflexões sobre o processo de construção de um terreiro de candomblé. Religião Soc. 2015 June;35:63–85.

59.	Taves A. Fits, trances, & visions: Experiencing religion and explaining experience from Wesley to James. Princeton, NJ, US: Princeton University Press; 1999. xii, 449 p. (Fits, trances, & visions: Experiencing religion and explaining experience from Wesley to James).

60.	Seligman R. Introduction: Stepping into the “Supernatural World” of Candomblé. In: Seligman R, editor. Possessing Spirits and Healing Selves: Embodiment and Transformation in an Afro-Brazilian Religion [Internet]. New York: Palgrave Macmillan US; 2014 [cited 2025 June 16]. p. 1–45. Available from: https://doi.org/10.1057/9781137409607_1

61.	Langdon EJ. New Perspectives of Shamanism in Brazil. Civilis Rev Int D’anthropologie Sci Hum. 2013 June 28;(61–2):19–35.

62.	Lin H, Gao H, Ye Z, Wang P, Tao L, Ke X, et al. Expectation enhances event-related responses to affective stimuli. Neurosci Lett. 2012 Aug 1;522(2):123–7.

63.	Tamir M, Bigman YE, Rhodes E, Salerno J, Schreier J. An expectancy-value model of emotion regulation: implications for motivation, emotional experience, and decision making. Emot Wash DC. 2015 Feb;15(1):90–103.

64.	van der Hoek S. Spirits and Bodies as an Interdependent Network of Relations and Approach to the Study of Religion in Brazil. Int J Lat Am Relig. 2024 June 1;8(1):194–208.

65.	Siuda-Ambroziak R, Mariano FP. Contemporary Transformations in the Brazilian Popular Catholic Festivals: the Case of the Holy Divine Spirit Festival (Festa do Divino). Int J Lat Am Relig. 2021 Dec 1;5(2):224–45.

66.	Zaluar A. Os homens de Deus: um estudo dos santos e das festas no catolicismo popular. Zahar Editores; 1983. 136 p.

67.	Kuhn W. UNDERSTANDING RELIGIOUS SYNCRETISM IN BRAZIL: CASES IN DUAL ALLEGIANCE WITH IMPLICATIONS FOR ADVENTIST MISSION. Rev Hermenêutica Descontinuada [Internet]. 2007 Jan 15 [cited 2025 June 16];7. Available from: https://adventista.emnuvens.com.br/hermeneutica/article/view/205

68.	Sanchis P. THE SOCIAL SCIENCES OF RELIGION IN BRAZIL. Debates NER [Internet]. 2007 Dec 22 [cited 2025 June 16]; Available from: https://seer.ufrgs.br/index.php/debatesdoner/article/view/2784

69.	Alves JE, Cavenaghi S, Barros LF, Carvalho AA de. Distribuição espacial da transição religiosa no Brasil1. Tempo Soc. 2017 Aug;29:215–42.

70.	Lopez MA. Significados de experiências de devoção: a crença em anjos. Memo Mem E História Em Psicol. 2010 Oct 26;19:26–39.

71.	Cravalho, M. A. An Invisible Universe of Evil: Supernatural Malevolence and Personal Experience Among Amazon Peasants. 1993.

72.	Shapiro M. Moral Topology and the Making of Cosmological Boundaries. Soc Anal. 2019 Sept 1;63:71–88.

73.	Moraes MJD. A filosofia ubuntu e o quilombo: a ancestralidade como questão filosófica. 2019;(32).

74.	Hess DJ. Ghosts and Domestic Politics in Brazil: Some Parallels between Spirit Possession and Spirit Infestation. Ethos. 1990;18(4):407–38.

75.	Iyanaga M. Cangoma Calling: Spirits and Rhythms of Freedom in Brazilian Jongo Slavery Songs ed. by Pedro Meira Monteiro, Michael Stone (review). Lat Am Music Rev. 2015;36(1):128–31.

76.	Lages SRC. Possessão e inversão da subalternidade: com a palavra, Pombagira das Rosas. Psicol Soc. 2012;24:527–35.

77.	Warren Jr. D. Spiritism in Brazil. J Inter-Am Stud. 1968 July;10(3):393–405.

78.	Martins L. Accounts of high strangeness: A Brazilian perspective on the paranormal and popular culture. In: The Paranormal and Popular Culture. Routledge; 2019.

79.	Santos RA dos, Martins LB. Hibridismo e cultura pop: alienígenas como ícones religiosos em contextos ayahuasqueiros e não ayahuasqueiros. REVER Rev Estud Religião. 2020 Dec 11;20(3):183–98.

80.	Magnani JGC. Neo-esotericism. In: Gooren HPP, editor. Encyclopedia of Latin American Religions [Internet]. Cham: Springer International Publishing; 2015 [cited 2025 June 17]. p. 1–11. Available from: https://link.springer.com/10.1007/978-3-319-08956-0_47-1

81.	Swami V, Pietschnig J, Stieger S, Voracek M. Alien psychology: Associations between extraterrestrial beliefs and paranormal ideation, superstitious beliefs, schizotypy, and the Big Five personality factors. Appl Cogn Psychol. 2011;25(4):647–53.

82.	Sá JFR de, Mota-Rolim SA. A paralisia do sono no folclore brasileiro e em outras culturas. Rev Estud Cult. 2023 Oct 21;(8):61–83.

83.	Cavalcante R de S, Banin VB, de Moura Ribeiro Paula NA, Daher SR, Habermann MC, Habermann F, et al. Effect of the Spiritist “passe” energy therapy in reducing anxiety in volunteers: A randomized controlled trial. Complement Ther Med. 2016 Aug 1;27:18–24.

84.	Stern FL. The Religious Profile of Brazilian Alternative Therapists: the Case of Naturology. Int J Lat Am Relig. 2019 June 1;3(1):156–69.
