# Supplementary material for: Making sense of the extraordinary – An investigation of the content and prevalence of appraisals of nonordinary experiences
Source: PLoS One. 2026 Aug 5;21(8):e0353126. doi: 10.1371/journal.pone.0353126 (PMC13440842; doi:10.1371/journal.pone.0353126)
Supplement: Supplementary file 5 [file pone.0353126.s007.docx]

Appraisals review letter

Dear Dr. Fischer,

Thank you for submitting your manuscript to PLOS ONE.

After careful consideration, we feel that it has a great deal of merit but some revisions are required. Therefore, we invite you to submit a revised version of the manuscript that addresses the points raised during the review process.

_______________________

Dear Dr David Smailes,

Thank you and the two reviewers for your constructive and encouraging comments on our paper now entitled “Making sense of the extraordinary – An investigation of the content and prevalence of appraisals of nonordinary experiences”. We appreciate the opportunity to strengthen our manuscript. Addressing your comments has allowed us to improve clarity and precision in our arguments and presentation of the data. If there are any remaining issues, please do not hesitate to contact us.

=======================================

The reviewers have provided a really useful set of comments that – when addressed – should improve the manuscript. In addition to the comments from the reviewers, please consider the comments from me below.

Sub01AE1 = I think it would be very helpful for readers if a simple hyperlink (such as <https://doi.org/10.1371/journal.pone.0287780.s001>) was inserted in the MS, that ‘takes’ readers to a copy of the original INOE. This would make it easier for the reader to get a good understanding of the range of experiences classed as an ‘NOE’ (although that may just reflect my lack of familiarity with this concept).

_______________________

**Our reply:**

Thank you for this great suggestion. We now included a link to the original study in the abstract, and we also included the full INOE as well as annotated changes to the appraisal section made in both English and Portuguese on the OSF and supporting information (Tables S1, S2 and S3). This should allow readers to gain a good understanding of the original inventory and the changes that we made.

Sub01AE2 = On line 355, the study’s pre-registration is mentioned and a citation is provided for that pre-registration. However, I do not think that the document cited is a pre-registration (although it may contain info about the pre-registration). I think this - <https://osf.io/ze6cb/overview> – link needs to be provided at line 355 instead? Apologies if I am mistaken.

_______________________

**Our reply:**

Thank you for highlighting this omission. You are correct about the missing link. We have now incorporated the link directly into the body of the text at line 457 to ensure readers can easily access the pre-registration of study 1.

Sub01AE3 = On lines 752-753, two papers are cited re: predictive coding and source-monitoring theories of hallucinations. I am not sure, however that these are particularly effective publications to cite here. Other papers – such as <https://academic.oup.com/schizophreniabulletin/article/38/4/683/1870142> or <https://www.cell.com/trends/cognitive-sciences/fulltext/S1364-6613(18)30282-1> or <https://www.annualreviews.org/content/journals/10.1146/annurev-clinpsy-032816-045145> - may be better to cite (although it does depend on the precise point you are making; especially if the aim is to cite a paper that supports the claim that being alone/socially isolated may affect predictive processing/source monitoring in a way that increases the likelihood of a hallucinatory experience).

_______________________

**Our reply:**

We appreciate the valuable input and excellent reference suggestions. We have now included citations to the three papers mentioned to support our claim regarding the contextual factor of being alone increasing the likelihood of hallucinations and further expand our argumentation regarding our claim (lines 895 to 902).

Sub01AE4 = Can you please make the OSF project page associated with the manuscript 'public' please? PLOS ONE operates single-blind peer review (reviewers can remain anonymous; authors do not), and so making the OSF project page will not 'unblind' the reviewers. The study data/materials were made available to me after you submitted the manuscript, but I think this was by adding me as a contributor to the project (I have now removed myself from the project, so that it is clear that I have no conflict of interest re: me acting as editor for this manuscript), and I believe the necessary data/scripts are available on the OSF. It would be great if the reviewers could examine this, too, when they review your resubmission.

_______________________

**Our reply:**

Thank you for this suggestion. We have now made all pages associated with this manuscript freely available. This includes the following links:

Data and scripts for studies 1 and 2: <https://osf.io/3fbx4/>

Validation method (study 1) preregistration: <https://osf.io/ze6cb>

Description of data collection for study 2: <https://osf.io/t3q2v>

=======================================

When submitting your revision, we need you to address these additional requirements.

1. Please ensure that your manuscript meets PLOS ONE's style requirements, including those for file naming. The PLOS ONE style templates can be found at

<https://journals.plos.org/plosone/s/file?id=wjVg/PLOSOne_formatting_sample_main_body.pdf> and

[https://journals.plos.org/plosone/s/file?id=ba62/PLOSOne_formatting_sample_title_authors_affiliations.pdf](https://track.editorialmanager.com/CL0/https:%2F%2Fjournals.plos.org%2Fplosone%2Fs%2Ffile%3Fid=ba62%2FPLOSOne_formatting_sample_title_authors_affiliations.pdf/1/010f019ae1f711f1-fe353553-9431-4051-a870-b9d853c33ad7-000000/nFx7stgftDypjgjBLBQDtxamcnhPaupIcKwJZKi3d14=240).

_______________________

**Our reply:**

Thank you for these links, we have now carefully checked all style requirements and revised our manuscript accordingly.

2. Thank you for stating in your Funding Statement:

“The work was supported by a grant from the John Templeton Foundation (Grant ID: 62651), the Pioneer Science Initiative of the D’Or Institute for Research and Education, Rio de Janeiro, Brazil and a grant by the Conselho Nacional de Desenvolvimento Científico e Tecnologico (Process: 309085/2023-3) to the last author.”

Please provide an amended statement that declares *all* the funding or sources of support (whether external or internal to your organization) received during this study, as detailed online in our guide for authors at <http://journals.plos.org/plosone/s/submit-now>.

Please also include the statement “There was no additional external funding received for this study.” in your updated Funding Statement.

_______________________

**Our reply:**

Thank you, we have now updated our funding statement as requested.

3. Thank you for stating the following financial disclosure:

“The work was supported by a grant from the John Templeton Foundation (Grant ID: 62651), the Pioneer Science Initiative of the D’Or Institute for Research and Education, Rio de Janeiro, Brazil and a grant by the Conselho Nacional de Desenvolvimento Científico e Tecnologico (Process: 309085/2023-3) to the last author.”

Please state what role the funders took in the study. If the funders had no role, please state: "The funders had no role in study design, data collection and analysis, decision to publish, or preparation of the manuscript."

If this statement is not correct you must amend it as needed.

Please include this amended Role of Funder statement in your cover letter; we will change the online submission form on your behalf.

_______________________

**Our reply:**

We have now amended our statement and declare that the funding bodies had no involvement in the design of the study, the collection, analysis, or interpretation of the data, nor in the preparation of the manuscript.

4. Please be informed that funding information should not appear in the Acknowledgments section or other areas of your manuscript. We will only publish funding information present in the Funding Statement section of the online submission form. Please remove any funding-related text from the manuscript.

_______________________

**Our reply:**

Thank you, we have now removed this section from our title page.

5. Your ethics statement should only appear in the Methods section of your manuscript. If your ethics statement is written in any section besides the Methods, please delete it from any other section.

_______________________

**Our reply:**

We have removed this information from the title page and included the approval numbers in the relevant sections of the two studies.

6. We note that you have referenced (Fischer R, Bortolini G., Bortolini TS, Maraldi EO, ED, Hartle L, et al. Revisiting the Unusual: ) which has currently not yet been accepted for publication. Please remove this from your References and amend this to state in the body of your manuscript: (Fischer R, Bortolini G., Bortolini TS, Maraldi EO, ED, Hartle L, et al. [Unpublished]”) as detailed online in our guide for authors

[http://journals.plos.org/plosone/s/submission-guidelines#loc-reference-style](http://track.editorialmanager.com/CL0/http:%2F%2Fjournals.plos.org%2Fplosone%2Fs%2Fsubmission-guidelines%23loc-reference-style/1/010f019ae1f711f1-fe353553-9431-4051-a870-b9d853c33ad7-000000/w3nUegdSPtr2t0iETGaMfj80n7Z8FR6V1JskujX0KPU=240).

_______________________

**Our reply:**

Thank you, we have updated the reference and now cite the relevant preprint (as per your guidelines, reference can include: Manuscripts on preprint servers, providing the manuscript has a citable DOI or arXiv URL).

7. Please include captions for your Supporting Information files at the end of your manuscript, and update any in-text citations to match accordingly. Please see our Supporting Information guidelines for more information: <http://journals.plos.org/plosone/s/supporting-information>.

_______________________

**Our reply:**

We apologize for the lack of clarity. We have reformatted our supporting information following the referred guidelines. Our previous “supplementary material” file was thus broken down into four different PDF files, all of which are captioned in the last section of our manuscript and are accordingly cited throughout the text.

8. If the reviewer comments include a recommendation to cite specific previously published works, please review and evaluate these publications to determine whether they are relevant and should be cited. There is no requirement to cite these works unless the editor has indicated otherwise.
_______________________

**Our reply:**

Thank you for giving us the liberty to decide whether to include specific suggestions.

We have added a total of 50 references (highlighted in yellow in the text of the revised manuscript) and removed 7 references.

Much of the added references relate to the requests to elaborate on the sources and process of the item generation (previously only available in the supporting information, now S1 Text file). In addition, we have included the references suggested by the editor and the reviewers. Thank you for these great pointers to relevant literature.

In this revision, the following papers were included:

1. Aquino TAA de, Correia APM, Marques ALC, Souza CG de, Assis Freitas HC de, Araújo IF de, et al. Atitude religiosa e sentido da vida: um estudo correlacional. Psicol Ciênc E Prof. 2009;29:228–43.
2. Behr D, Braun M, Aiglstorfer L. Showcasing the usefulness of web probing: Do subtle variations in questionnaire translation lead to different survey responding? Qual Quant. 2024 Oct 1;58(5):4117–35.
3. Blackmore SJ. A psychological theory of the out-of-body experience. J Parapsychol. 1984;48(3):201–18.
4. Bortolini T, Laport MC, Bortolini GN, de Sá CA, Fischer R, Moll J. Mapping the Content Landscape of Self-Transcendent Emotional Experiences Using a Mixed-Method Approach [Internet]. PsyArXiv; 2026 [cited 2026 Jan 16]. Available from: https://osf.io/preprints/psyarxiv/rsy4b_v1/
5. Brett CMC, Johns LC, Peters EP, McGuire PK. The role of metacognitive beliefs in determining the impact of anomalous experiences: a comparison of help-seeking and non-help-seeking groups of people experiencing psychotic-like anomalies. Psychol Med. 2009 June;39(6):939–50.
6. Callaghan BL, Tottenham N. The stress acceleration hypothesis: Effects of early-life adversity on emotion circuits and behavior. Curr Opin Behav Sci. 2016;7:76–81.
7. Cardeña E, Schaffler Y. “He Who Has the Spirits Must Work a Lot”: A Psycho‐Anthropological Account of Spirit Possession in the Dominican Republic. Ethos. 2018 Dec;46(4):457–76.
8. Collao LD, Peñalba A. Cognición, experiencia musical y conciencias alteradas: la influencia de las ciencias cognitivas en el estudio sobre la música y el trance. El Oído Pensante [Internet]. 2019 Feb 1 [cited 2025 June 16];7(1). Available from: http://revistascientificas.filo.uba.ar/index.php/oidopensante/article/view/7542
9. Corlett PR, Horga G, Fletcher PC, Alderson-Day B, Schmack K, Powers AR. Hallucinations and Strong Priors. Trends Cogn Sci. 2019 Feb;23(2):114–27.
10. De Boer EM. Out of Body, Loss of Self: Spiritual or Scary? Religions. 2020 Oct 28;11(11):558.
11. Dilevski N, Paterson HM, Walker SA, van Golde C. Adult memory for specific instances of a repeated event: a preliminary review. Psychiatry Psychol Law. 28(5):711–32.
12. Dunn EC, Nishimi K, Gomez SH, Powers A, Bradley B. Developmental timing of trauma exposure and emotion dysregulation in adulthood: Are there sensitive periods when trauma is most harmful? J Affect Disord. 2018 Feb;227:869–77.
13. Eisenberg ML, Zacks JM, Flores S. Dynamic prediction during perception of everyday events. Cogn Res Princ Implic. 2018 Dec 29;3(1):53.
14. Esperandio MRG, Marques LF. The Psychology of Religion in Brazil. Int J Psychol Relig. 2015 Oct 2;25(4):255–71.
15. Fischer R, Bortolini G, Bortolini T, Maraldi E de O, Hartle L, Varejao MVDL, et al. Revisiting the Unusual: Investigating the Prevalence and Validity of Nonordinary Experiences as a Window into Consciousness [Internet]. In Review; 2025 [cited 2025 Dec 17]. Available from: https://www.researchsquare.com/article/rs-6907742/v1
16. Franco Corso SJ, O’Malley KY, Subaiya S, Mayall D, Dakwar E. The role of non-ordinary states of consciousness occasioned by mind-body practices in mental health illness. J Affect Disord. 2023 Aug 15;335:166–76.
17. Gaynor K, Ward T, Garety P, Peters E. The role of safety-seeking behaviours in maintaining threat appraisals in psychosis. Behav Res Ther. 2013 Feb;51(2):75–81.
18. Griffin JD, Fletcher PC. Predictive Processing, Source Monitoring, and Psychosis. Annu Rev Clin Psychol. 2017;13(1):265–89.
19. Havik G, Elands BHM, van Koppen CSA (Kris). An encounter with one’s deeper self and energy: A phenomenological study among spiritually engaged individuals in the Netherlands. Ecopsychology. 2015;7(2):75–83.
20. Hoemann K, Xu F, Barrett LF. Emotion words, emotion concepts, and emotional development in children: A constructionist hypothesis. Dev Psychol. 2019 Sept;55(9):1830–49.
21. Ip EH, Magee MF, Youssef GA, Chen SH. Gleaning Information for Cognitive Operations from Don’t Know Responses in Cognitive and Noncognitive Assessments. Multivar Behav Res. 2019;54(2):159–72.
22. Irwin HJ, Schofield MB, Baker IS. Dissociative tendencies, sensory-processing sensitivity and aberrant salience as predictors of anomalous experiences and paranormal attributions. J Soc Psych Res. 2014;78(917[4]):193–206.
23. Kuhn W. Understanding religious syncretism in Brazil: Cases in dual allegiance with implications for adventist mission. Rev Hermenêutica [Internet]. 2007 Jan 15 [cited 2025 June 16];7. Available from: https://adventista.emnuvens.com.br/hermeneutica/article/view/205
24. Laurens KR, Hobbs MJ, Sunderland M, Green MJ, Mould GL. Psychotic-like experiences in a community sample of 8000 children aged 9 to 11 years: an item response theory analysis. Psychol Med. 2012 July;42(7):1495–506.
25. Lopez MA. Significados de experiências de devoção: a crença em anjos. Memo Mem E História Em Psicol. 2010 Oct 26;19:26–39.
26. Maraldi E de O, Machado FR, Zangari W. Importance of a Psychosocial Approach for a Comprehensive Understanding of Mediumship. J Sci Explor 2010 Vol 24 Issue 2 P181. 2010;
27. Mason O, Claridge G. The Oxford-Liverpool Inventory of Feelings and Experiences (O-LIFE): Further description and extended norms. Schizophr Res. 2006 Feb;82(2–3):203–11.
28. Monteiro de Barros MC, Leão FC, Vallada Filho H, Lucchetti G, Moreira-Almeida A, Prieto Peres MF. Prevalence of spiritual and religious experiences in the general population: A Brazilian nationwide study. Transcult Psychiatry. 2022 Apr 6;13634615221088701.
29. Moreira-Almeida A, Cardeña E. Differential diagnosis between non-pathological psychotic and spiritual experiences and mental disorders: a contribution from Latin American studies to the ICD-11. Braz J Psychiatry. 2011;(33):21–8.
30. Muthukrishna M, Doebeli M, Chudek M, Henrich J. The Cultural Brain Hypothesis: How culture drives brain expansion, sociality, and life history. PLoS Comput Biol. 2018 Nov 8;14(11):e1006504.
31. Őri D, Vass E, Vajsz K, Vincze K, Sztancsik V, Szemán-Nagy A, et al. Psychometric validation of the Reported and Intended Behaviour Scale (RIBS) in Hungary with a particular focus on “Don’t know” responses and further scoring recommendations. BMC Public Health. 2023 Sept 12;23(1):1773.
32. Peters E, Ward T, Jackson M, Morgan C, Charalambides M, McGuire P, et al. Clinical, socio-demographic and psychological characteristics in individuals with persistent psychotic experiences with and without a “need for care.” World Psychiatry Off J World Psychiatr Assoc WPA. 2016 Feb;15(1):41–52.
33. R Core Team. R: A Language and Environment for Statistical Computing (version 4.3.0) [Internet]. Vienna, Austria.: R Foundation for Statistical Computing; 2023. Available from: https://www.R-project.org/
34. Sá JFR de, Mota-Rolim SA. A paralisia do sono no folclore brasileiro e em outras culturas. Rev Estud Cult. 2023 Oct 21;(8):61–83.
35. Scherer KR, Schorr A, Johnstone T. (eds.). Appraisal processes in emotion: Theory, methods, research. New York, NY, US: Oxford University Press; 2001. 478 p.
36. Seligman R. Introduction: Stepping into the “Supernatural World” of Candomblé. In: Seligman R, editor. Possessing Spirits and Healing Selves: Embodiment and Transformation in an Afro-Brazilian Religion [Internet]. New York: Palgrave Macmillan US; 2014 [cited 2025 June 16]. p. 1–45. Available from: https://doi.org/10.1057/9781137409607_1
37. Shapiro M. Moral Topology and the Making of Cosmological Boundaries. Soc Anal. 2019 Sept 1;63:71–88.
38. Steffen EM, Wilde DJ, Cooper CE. Affirming the positive in anomalous experiences: A challenge to dominant accounts of reality, life, and death. In: The Routledge international handbook of critical positive psychology. New York, NY, US: Routledge/Taylor & Francis Group; 2018. p. 227–44. (Routledge international handbooks).
39. Stern FL. The Religious Profile of Brazilian Alternative Therapists: the Case of Naturology. Int J Lat Am Relig. 2019 June 1;3(1):156–69.
40. Stevens JS, van Rooij SJH, Jovanovic T. Developmental Contributors to Trauma Response: The Importance of Sensitive Periods, Early Environment, and Sex Differences. In: Vermetten E, Baker DG, Risbrough VB, editors. Behavioral Neurobiology of PTSD [Internet]. Cham: Springer International Publishing; 2018 [cited 2025 June 16]. p. 1–22. Available from: https://doi.org/10.1007/7854_2016_38
41. Tate AJM. Contributory injustice in psychiatry. J Med Ethics. 2019 Feb;45(2):97-100.
42. Toutountzidis D, Gale TM, Irvine K, Sharma S, Laws KR. Childhood trauma and schizotypy in non-clinical samples: A systematic review and meta-analysis. PloS One. 2022;17(6):e0270494.
43. Underwood LG, Teresi JA. The daily spiritual experience scale: development, theoretical description, reliability, exploratory factor analysis, and preliminary construct validity using health-related data. Ann Behav Med. 2002 Feb 1;24(1):22–33
44. van der Hoek S. Spirits and Bodies as an Interdependent Network of Relations and Approach to the Study of Religion in Brazil. Int J Lat Am Relig. 2024 June 1;8(1):194–208.
45. Verduyn P, Delaveau P, Rotgé JY, Fossati P, Van Mechelen I. Determinants of Emotion Duration and Underlying Psychological and Neural Mechanisms. Emot Rev. 2015 Oct 1;7(4):330–5.
46. Wantoch S. The Meta of Madness: How the social framing of anomalous experience affects its ontology [Internet] [Dissertation]. [Sheffield]: University of Sheffield; 2022. Available from: https://etheses.whiterose.ac.uk/id/eprint/32646/
47. Waters F, Allen P, Aleman A, Fernyhough C, Woodward TS, Badcock JC, et al. Auditory Hallucinations in Schizophrenia and Nonschizophrenia Populations: A Review and Integrated Model of Cognitive Mechanisms. Schizophr Bull. 2012 July 1;38(4):683–93.
48. Weingarten E, Zauberman G, Diehl K. Duration sensitivity of key moments. Cognition. 2021 Sept 1;214:104750.
49. Zacks JM, Speer NK, Swallow KM, Braver TS, Reynolds JR. Event perception: A mind-brain perspective. Psychol Bull. 2007;133(2):273–93.
50. Zacks JM. Event Perception and Memory. Annu Rev Psychol. 2020 Jan 4;71(Volume 71, 2020):165–91.

9. Please review your reference list to ensure that it is complete and correct. If you have cited papers that have been retracted, please include the rationale for doing so in the manuscript text, or remove these references and replace them with relevant current references. Any changes to the reference list should be mentioned in the rebuttal letter that accompanies your revised manuscript. If you need to cite a retracted article, indicate the article’s retracted status in the References list and also include a citation and full reference for the retraction notice.
_______________________

**Our reply:**

We have now carefully revised and updated our reference list. To the best of our knowledge, we do not cite any retracted papers. As mentioned in the previous reply, we have added a total of 50 references to our revised manuscript, which are marked in yellow both in the body of the text and in the references section.

=======================================

Reviewers' comments:

**Reviewer #1:** “Thank you for this paper. I have some minor revisions.

Study I

Abstract:
A stronger conclusion is needed. The meaning and importance of the paper is not clear.”
_______________________

**Our reply:**

We agree that the conclusion of the abstract did not sufficiently emphasize the central implications of our validation and prevalence studies. We have therefore added relevant information highlighting the significance of our findings across both studies (lines 61–70). Particular attention was given to the results showing unimodal distributions for most contextual features and appraisals across all experiences, which point to shared cognitive frameworks used to interpret these types of experiences and common contextual patterns in which they occur.

Most importantly, as suggested by the reviewer, we included a remark on the prevalence of appraisals related to the perceived immediate and long-term effects of these experiences. Notably, we observed a higher prevalence of positive evaluations of long-term effects, even when experiences were sometimes perceived as immediately negative. This pattern suggests a potential role of nonordinary experiences in supporting well-being over the longer term.

**Reviewer #1: “**Introduction:

It is difficult to follow across the Tables what the included appraisal items were. How they were worded? and why certain appraisal were included or excluded. The paper would benefit from a copy of the Appraisal Form either in a new Table, In Table 1 instead of the adaptations column or in supplemental material"

_______________________

**Our reply:**

Thank you for this suggestion. We have updated Table 2 in our manuscript, which contains the English version of the appraisal items, to clearly indicate which items from the original validation study by Taves et al. (2023) were altered or extended and which items were newly developed for the current study. We have also added a comprehensive list of all response options for each item to our supporting information (S3 Table). The theoretical foundations for item extensions and additions have been reformatted as S1 Text in the supporting information. Finally, the Portuguese version of the appraisal items is available on the OSF at <https://osf.io/3fbx4>.

**Reviewer #1:** “It was unclear whether appraisals of personal/ non personal, benevolent/ benign/ malevolent, omnipotence/ omniscience/ neutrality were included? These are important appraisals with the AANEX, and Beliefs about Voices questionnaires, and are predictive of distress and believability. If they were excluded, a justification should be given. God’s intentions, for instance, maybe appraised as benevolent or malevolent.”

_______________________

**Our reply:**

This is a very insightful observation regarding the specific nature of agentic and personal appraisals. The original set of appraisals was derived from the Inventory of Non-Ordinary Experiences (INOE) and comprised seven items: *mental state*, *agent*, *reason*, *science*, *spiritual*, *life effect*, and *impact*.

Our aim was to better capture features relevant for discriminating and predicting adaptive versus nonadaptive reactions for mental health, as well as relevant interpretation frameworks within the Brazilian cultural context. A second aim was to create appraisals that would be applicable across the relatively wider set of experiences, to be able to assess appraisal processes more broadly. For that purpose, we extended and validated a set of new appraisal items, including *context*, *duration*, *frequency*, *onset*, *effect moment*, *distress*, *community*, *control*, and *induction*. In addition, we expanded the response options for the *agent* and *reason* items and added the option *awake and alert* to the *mental state* question.

The AANEX was a source for the original list of appraisals (see Taves et al., 2023). We were aware of the Beliefs about Voices instruments during item construction, but it was not an explicit source for developing our items. One specific reason for not including items from this instrument was the domain specificity. Several of the items in the Beliefs about Voices Questionnaire are highly specific to the phenomena of hearing voices, whereas the INOE comprises a range of experiences for which these specific appraisal items would not be adequate.

However, we believe that we were able to capture most of the appraisal categories of the AANEX and Beliefs about Voices indirectly. Specifically:

1) The *personal/nonpersonal* appraisal in AANEX corresponds closely to the INOE *agent* item, which we expanded to include a wider range of personal agents, as well as the explicitly nonpersonal option *“I do not believe anyone made me have this experience.”*

2) The *benevolent/benign/malevolent* dimension is adequately captured by combining a number of appraisals: a) the newly introduced *effect moment* item, which assesses whether the experience was perceived as immediately helpful, disturbing, or neutral; b) the *distress* item, which evaluates whether the experience elicited joy or suffering; (c) the *agent* item, assessing personal versus nonpersonal agency; and (d) the *reason* item, evaluating whether the experience happened by chance or due to specific, meaningful, self-referenced reasons. Together, these appraisals provide a coherent framework for inferring benevolent versus malevolent interpretations.

3) We did not include an item that directly targets the *omnipotence* construct assessed in the Beliefs About Voices questionnaire. Our expansion of the *agent* item may capture some of these associations more indirectly via an ethnographic exploration of the expanded list of entities such as gods, the Holy Spirit, angels, saints, demons, Afro-Brazilian entities or deities, ancestors, among others. These response options provide greater nuance in relation to the perceived entity involved. In addition, we highlight the *control* item, which assesses whether individuals experienced control over the event or attempted to exert control, a factor known to be a relevant predictor of distress and closely (inversely) related to the notion of omnipotence.

**Reviewer #1:** “Cognitive theories would suggest that it is when an negative appraisal is matched with safety behaviours that a maintaining process of distress occurs. (for instance <https://doi.org/10.1016/j.brat.2012.10.008>). A reflection on how benign experiences may become psychopathological would be helpful.”

_______________________

**Our reply:**

Thank you for this excellent suggestion. We have now included these references and briefly elaborate on this point as an important avenue for future research (see lines 980-987).

**Reviewer #1:** “Methods

No demographic data was collected due to time restrictions - this is a limitation and should be noted especially in relation to validity and generalisability”
_______________________

**Our reply:**

Thank you for highlighting this important limitation. We have now included this point specifically in the discussion section for study 1. We write:

“One important limitation of our study was that no information on demographics was available. This implies that we cannot make strong claims about the external validity and generalizability of our validation study. Comparative work using this panel service and samples invited through similar methods used in this study suggested that samples tend to be representative of the larger population in terms of age, gender and geographic location, but are typically more well educated than expected based on census data” (page 21, lines 553 to 558).

**Reviewer #1:** “Sample size was determined by the stopping criteria described in the section entitled 'Decision criteria for item validation” could this be explained further in the text. N=160 seems small for a multi-item scale and strong justification is needed.

_______________________

**Our reply:**

Thank you for highlighting this important point. We now further explain the reasoning for our sample size according to the **Response Process Evaluation** (RPE) protocol in our methods section (lines 392 to 408, 438 to 449). This protocol requires 80% comprehension among at least 20 participants for an item to be considered provisionally validated. Given our number of appraisal items and the 20 participants threshold, we would need a minimum of 40 participants, half of which would be assigned to the original items (version A) and the other half to new items (version B). Therefore, our final sample of 160 participants exceeded the methodologically justified minimum of 40 participants required by the RPE protocol. Data integrity was ensured through quality control measures implemented in the questionnaire, which excluded participants providing random, unintelligible, or excessively vague responses.

**Reviewer #1:** “Results:

Missing data should be discussed at a sample and item level.”
_______________________

**Our reply:**

We thank the reviewer for raising this point. We clarify that, by design, there was no missing data at the sample or item level in either study.

The way we collected data required participants to complete all items before advancing in the survey to the next page. As a result, incomplete data could not occur, but individuals could leave the study at any point. At the item level, the only missingness in the dataset for study 1 is structural and reflects the experimental design: participants were randomly assigned to either the original appraisal items (version A) or the new appraisal items (version B). Items from the non-assigned version were not administered and are therefore structurally missing, as illustrated in Figure 1 in the manuscript. Within each assigned version, all appraisal items were mandatory, resulting in complete item-level data and no need for imputation.

We now write:

“Regarding missing data, no case level imputation was applied since incomplete interviews were not retained in the final sample. At the item level, missingness in the dataset reflects the randomization either to the original (version A) or new appraisal items (version B), as illustrated in Figure 1. Items from the non-assigned version were not administered and are therefore structurally missing. Within each version, participants were required to respond to the complete set of appraisal items as part of the RPE protocol, so there was effectively no item non-response and no item-level imputation.” (Study 1, lines 458 to 464)

**Reviewer #1:** “VS = full wording should be used”
_______________________

**Our reply:**

We now spell out the full wording of VS throughout the manuscript.

**Reviewer #1:** “The table location and formatting through out the document is difficult to follow.”

_______________________

**Our reply:**

We have now updated the formatting and placement of the tables in our revised manuscript.

**Reviewer #1:** “Study II

Results

Item level missing data should be report”

_______________________

**Our reply:**

Thank you for highlighting this missing information. In Study 2, we required participants to provide complete responses in order to advance in the survey, which precluded item-level non-response and incomplete cases. As a result, there was no missing data at either the sample or item level, and no imputation or other missing data treatment was necessary.

We now write on page 33:

“We required complete responses in order for participants to advance in the survey. For this reason, we did not have any missing information at item level and no imputations or other missing data treatment was necessary.” (lines 658-660)

**Reviewer #1:** "What was the missing data strategy?”

_______________________

**Our reply:**

We assume that you refer to strategies of how missing data was handled. Since we required responses to all items for participants to advance to the next section, we did not have missing data that required imputation or some other form of missing data treatment. We have now clarified this in the manuscript on page 33, as stated above.

**Reviewer #1:** “Table 4 should be presented in the results section”

_______________________

**Our reply:**

We have now moved it to the result section of study 2. We apologies for this formatting issue with the previous version.

**Reviewer #1:** “Discussion

“This is particularly important when considering that a vast majority of experiences, including negative experiences, in the long run, were associated with positive effects overall. This shows the enormous potential of human resilience and requires more focused follow-up studies.”

I’m particularly struck by this comment. It fits the wider literature, (i.e., <https://doi.org/10.1002/wps.20301>) and highlights the importance of protective factors, and natural human resilience. I wonder would this be an appropriate conclusion for the abstract?”
_______________________

**Our reply:**

Thank you for your positive evaluation of our work and the suggested literature. We have now included this line of reasoning in the abstract, incorporated Peters et al. reference (lines 273, 276) and also updated our revised discussion section.

**Reviewer #2: “**Overview:

These studies offer valuable contributions to the literature on non-ordinary experiences, addressing the important and thorny issue of attempting to disentangle NOEs and appraisals of these experiences. The studies and analyses themselves are generally well done. However, the paper in terms of its framing, language and overall communication of the studies and their position in the wider literature needs substantial editing.”

_______________________

**Our reply:**

Thank you so much for your positive reading of our manuscript.

“There are times where the goals and scope of the paper are unclear. There is also a need for a fair bit of editing in terms of language precision. I have included a number of specific language edits in my recommendations below, but please proofread the whole paper for grammar and clarity of language.”

_______________________

**Our reply:**

Thank you for your constructive and encouraging comments. We have now revised the introduction to make the goals of the paper clearer. We have edited the paper to improve grammar and clarity of language.

**Reviewer #2:** “Changes needed:

The definition and scope of NOEs as a concept need to be made clear at the outset. At one point you refer to “subjectively salient” experiences (lines 104-106) and towards the end of the paper (705-706) they appear to be officially defined this way. However, this needs to be stated clearly early on.”

_______________________

**Our reply:**

We have now defined the NOEs consistently throughout the introduction. We now bring the formal definition in the first paragraph of the manuscript on page 4 (lines 84 to 87).

**Reviewer #2:** “Furthermore, a discussion of the reasoning behind focusing on experiences which are subjectively salient, as discussed by Taves et al. (2023), is needed. You’ve described quite a wide array of experiences ranging from voice-hearing to love and despair as non-ordinary experiences (e.g. lines 96-99). The paper needs to directly address the fact that this wide range of experiences are being discussed as part of the same category.”

_______________________

**Our reply:**

This is an important point. The inventory was developed in a way to avoid disciplinary biases due to researcher-defined types of experience that may exclude phenomenologically similar or dissimilar experiences due to some specific researcher defined criterion (see Taves et al., 2023). Therefore, the development of the inventory was based on the following criteria: a) these experiences had to be subject to conflicting interpretations, b) interpretations ideally should involve strong claims in terms of the causation or impact of these experiences, c) experiences typically were found to have some profound positive or negative impact on people’s lives in the studies reported in the literature (see the section “A subject-dependent definition of “nonordinary”” in Taves et al. 2023). Therefore, the focus was not to select experiences from the same category, but rather select experiences that vary widely and have been examined in highly distinct ways in the literature. We believe that this approach is of value when examining appraisal processes because it will elicit a variety of appraisals that can be studied and probed further. We have elaborated on this on pages 5-6 (lines 145 to 173) and we also note the relevance of this diverse set of experiences for the study of appraisal processes on page 7 (lines 197 to 199).

**Reviewer #2:** “The paper should also provide a short, clear definition of appraisal early on. As reference, Taves et al.’s original paper includes the following clear definition: “Appraisal, as used here, refers to the outcome of a multi-level process that humans and other animals rely on when determining what is happening, that is, to interpret situations and events””

_______________________

**Our reply:**

Thank you for this great suggestion. We have now updated our definition on page 4 (lines 95 to 99), drawing upon the work by Taves et al.

**Reviewer #2:** “89 There is a conjunction missing before the word “strong””

_______________________

**Our reply:**

We apologize, but we were unable to identify the specific line and word combination. We have removed one instance of strong in the sentence ‘strong connection to something greater’. As indicated earlier, we have carefully proofread the whole manuscript and edited grammar and clarity throughout.

**Reviewer #2:** “97-99 “On the other hand, phenomenologically comparable experiences are being used in psychiatric manuals as symptoms of dissociation or hallucinations” This is unclear because hallucination is a symptom, not a condition. One way to fix this would be say change the word “hallucinations” to “psychosis”"

_______________________

**Our reply:**

Thank you for highlighting our imprecision in the previous version. We have now updated this section and refer to symptoms of dissociative disorders or psychosis (see line 95).

105-106 “In this study, we aim to focus squarely on the appraisals and situational factors surrounding a broad set of experiences. When and where are subjectively salient experiences likely to happen?” The word “squarely” doesn’t make sense here. Remove the word “squarely” or change it to something like “in particular” or “specifically”.

_______________________

**Our reply:**

We have removed the term and reorganized the section to make it more coherent and improve clarity.

**Reviewer #2:** “In the introduction, there are a number of places where specific examples would make the paper easier to follow. For instance: 120-122: provide an example of the way in which the description of the experience may incorporate an appraisal.”

_______________________

**Our reply:**

Thank you for bringing this to our attention. To enhance clarity, we have incorporated concrete examples in the referenced section (lines 107 to 113), highlighting two instruments that embed a priori researcher interpretations to nonordinary experiences. Specific items from each instrument are provided to illustrate this point more effectively.

We now write:

“An example of an inherently spiritual/religious framework can be seen in the Daily Spiritual Experience Scale (12), which frames an experience of a nonordinary presence positively (“I feel God’s presence”). In contrast, a pathological framing of NOEs is observable in clinical scales such as The Oxford-Liverpool Inventory of Feelings and Experiences (O-LIFE) (14) which interprets similar experiences as traits of schizotypal personality and includes explicitly negative appraisals within the query about the experience (e.g., “evil presence”).”

We believe these examples illustrate how sometimes phenomenologically similar NOEs may be described as either indicators of spiritual growth or risk for psychopathology, depending on the theoretical assumptions that underlie the instrument.

**Reviewer #2:** “115-116 “to see if there are patterns that can be used to describe the human cognitive system used to make sense of” This sentence is unclear. I think you mean “patterns that can shed light on the cognitive processes involves in making sense of…”

_______________________

**Our reply:**

Thank you for this great suggestion. We have incorporated your proposed phrasing, as it aligns precisely with our original intended meaning while making the text considerably clearer.

**Reviewer #2:** “118-120: do you actually mean “conceptual overlap” or do you mean that the causal relationships are difficult to disentangle?”

_______________________

**Our reply:**

This is indeed a crucial distinction. You are correct in noting that causal relationships (and directionality in causal linkages) between nonordinary experiences and psychopathological constructs are often difficult to disentangle. However, our focus here is specifically on the conceptual overlap between these two categories of phenomena, which introduces an additional layer of complexity to this discussion.

We believe the boundary separating many forms of nonordinary experience from psychopathological symptoms of psychosis or dissociation is notably porous. Certain experiences, including perceptual alterations, alterations of the self, and automatisms, may be interpreted as psychopathological signs and symptoms depending on their alignment with the individual's sociocultural context, the individual's appraisal of these experiences, associated levels of distress, need for care and the intensity and frequency with which they manifest. It is this context-dependent “interpretive ambiguity” that we characterize as conceptual overlap.

**Reviewer #2:** “159-160: “We draw on multiple theoretical approaches”. More info is needed. The following section goes on to describe only one theoretical framework.”

_______________________

**Our reply:**

Thank you for highlighting our need to be more precise. We now removed this statement and explicitly refer to appraisal theory only. We have reworded the opening statement to better capture the theoretical foundations we develop throughout the paragraph:

“Drawing insight from two recent review studies that examined the role of nonordinary experiences on mental health (15) and a systematic review of dissociative experiences in Brazil (16) and taking inspiration from appraisal theory (9–11,21), we suggest and test additional appraisals that may be of importance, with a particular focus on possible mental health associations. We chose appraisal theory because this approach posits that emotions arise from the evaluations individuals make of events and situations, explaining variations in emotional responses across individuals and over time" (lines 181 to 185).

**Reviewer #2:** “232-233: “A substantive number of participants in the original validation study thought that chance might be a good reason” The word should be “substantial”, not “substantive” and the sentence overall needs proof-reading.”

_______________________

**Our reply:**

The grammatical error has been corrected, and the sentence has been rewritten for improved clarity and tone: “A substantial proportion of participants in the original validation study indicated that pure chance could constitute a valid explanatory reason” (lines 259 to 260). Thank you for bringing this error to our attention.

**Reviewer #2:** “240-241 I think you are correct that there is a need for broader information about situational factors. But the paper needs to explicitly make the case for WHY this information is needed. Overall, there needs to be a clearer explanation given as to why Taves et al.’s original list of appraisal options is insufficient.”

_______________________

**Our reply:**

Thank you for raising this very important point. We have now elaborated on why it is important to extend the appraisal and situational characteristics questions. Please see our revised section on page 9 (lines 269 to 278).

**Reviewer #2:** “245-253: More information is needed about how this study’s list of new appraisal options were generated from the previous list.”

_______________________

**Our reply:**

Thank you for highlighting the lack of clarity regarding the construction of new appraisal items. In the section "Extension of the Nonordinary Experiences Appraisals" (page 9-11), we have included new paragraphs describing the motivations, theoretical basis, and references for the extension process (lines 280 to 341).

The main sources for our item construction were two previous systematic reviews on (a) associations between nonordinary experiences and wellbeing and (b) mediumship/possession and dissociation experiences in Brazil. The former identified 28 previous measures of nonordinary experiences and health, which also served as a source for our new items. In this revised section, we now describe in greater detail the rationale for each new appraisal item. Additionally, we clarify in the text that further elaboration on the theoretical basis of the new appraisal items and response options can be found in the supporting information (S1 Text).

More information about phrasing alterations and response option extensions to the original items (not including entirely new items) can also be found in the supporting information (S2 Table). Finally, the full list of appraisal items validated in Study 1 and used in Study 2 is available in S3 Table.

**Reviewer #2:** “256. Explain what you mean by “based on these insights”. Which insights? Did you take all the appraisal options listed in these papers and divide them into the categories that you outline? Please explain the process in greater detail. Here, once again, the explanation would benefit from reference to specific examples.”

_______________________

**Our reply:**

We agree that this section lacked sufficient detail. In response, we have revised the paragraph to provide additional context regarding our two previous systematic reviews on nonordinary experiences and the rationale underlying each of our appraisal extensions (lines 280 to 341). As mentioned in the previous reply to the reviewer’s comment, we clarify in the text that the detailed elaboration on the theoretical basis of the new appraisal items and response options can be found in the supporting information (lines 348 to 351).

**Reviewer #2:** “381: Change “till” to “until”"

_______________________

**Our reply:**

This has been corrected accordingly.

**Reviewer #2:** “403: Explain (perhaps via a brief example) the distinction between cause and reason”

_______________________

**Our reply:**

We appreciate you raising this point. We agree that this distinction needed clarification and we have now expanded the discussion (lines 535 to 543). Reasons are invoked when we perceive an event as resulting from an intentional agent's actions. Within this framework, to explain an event by giving a reason is to attribute goals, motives, or purposes to an agent (whether human or nonhuman). Reasons thus presuppose intentionality. Causes, by contrast, explain events understood as unintended or non-agentic. Causal explanations rely on chains of antecedent conditions or mechanisms.

**Reviewer #2:** “411-413: I think you mean “selected by a small number of participants”. If so, change to make clearer.”

_______________________

**Our reply:**

You are correct. Within the context of our study, "selected" and "participants" are indeed more appropriate and precise than "chosen" and "individuals." We have therefore adopted this suggestion, writing: “One issue is that some of the categories were selected by a relatively small number of participants.”

**Reviewer #2:** “424 There are words missing in the title: appraisals across what?”

_______________________

**Our reply:**

We apologize for this formatting error and have corrected it to "Population level prevalence of appraisals across Brazilian adults” (line 568). Thank you for bringing this to our attention.

**Reviewer #2:** “439: Should be "a unique" not "an unique””

_______________________

**Our reply:**

We have corrected this grammatical error and highly appreciate your careful reading of our manuscript.
