# Supplementary material for: Making sense of the extraordinary – An investigation of the content and prevalence of appraisals of nonordinary experiences
Source: PLoS One. 2026 Aug 5;21(8):e0353126. doi: 10.1371/journal.pone.0353126 (PMC13440842; doi:10.1371/journal.pone.0353126)
Supplement: Supplementary file 6 [file pone.0353126.s005.zip › CEP-material/PB_PARECER_CONSUBSTANCIADO_CEP_5819411.pdf]

PARECER CONSUBSTANCIADO DO CEP

DADOS DO PROJETO DE PESQUISA

Título da Pesquisa: Adaptação e validação do Inventário de Experiências Não-Ordinárias para o português brasileiro

Pesquisador: Ronald Fischer

Área Temática:

Versão: 1

CAAE: 65573322.6.0000.5249

Instituição Proponente: INSTITUTO D'OR DE PESQUISA E ENSINO

Patrocinador Principal: INSTITUTO D'OR DE PESQUISA E ENSINO

DADOS DO PARECER

Número do Parecer: 5.819.411

Apresentação do Projeto:

Trata-se do Projeto: Adaptação e validação do Inventário de Experiências Não-Ordinárias para o português brasileiro com a seguinte introdução:

Experiências não-ordinárias, como a sensação de ser tocado ou ouvir uma voz quando ninguém está por perto, sentir que os próprios pensamentos parecem controlados externamente, ou ainda perder a percepção de tempo e espaço, podem ser interpretadas como religiosas, anômalas ou patológicas, dependendo da origem cultural e das características individuais de quem as vivencia (Acunzo et al., 2020; Cardeña et al., 2014; Larøi et al., 2014). Além disso, pessoas podem interpretar essas experiências como muito positivas e altamente significativas ou como perturbadoras, intrigantes ou sem sentido. Em busca de compreensão e em função de tais interpretações, é comum consultar especialistas religiosos ou médicos. O tipo de especialista consultado pode resultar na espiritualização ou medicalização da experiência (Moreira-Almeida et al., 2008).

Diversos estudos indicam que também podem existir diferenças de interpretação entre pesquisadores e participantes de estudos, mesmo considerando experiências aparentemente semelhantes, mas que podem ser interpretadas como religiosas, espirituais, anômalas, paranormais e/ou patológicas (Luhrmann, 2017; McCauley & Graham, 2020; Schmidt, 2014, 2015). Apesar da sobreposição fenomenológica de tais experiências (Hageman et al., 2011;

McCauley & Graham, 2020; Moreira-Almeida et al., 2006), muitos instrumentos usam descrições que definem as experiências a priori como religiosas, espirituais, anômalas, paranormais e/ou patológicas. Consequentemente, é difícil obter estimativas confiáveis da frequência dessas experiências ao longo da vida devido à sobreposição dos construtos, do desenho das medidas e da formulação das perguntas. Não apenas itens semelhantes são incluídos em vários construtos definidos pelo pesquisador sob diferentes rótulos, mas muitos itens são redigidos de maneiras que lhes dão conotações religiosas, paranormais ou psicopatológicas (Taves, 2020), o que pode alterar a resposta do participante que não interpreta a experiência da mesma forma.

A interpretação também parece influenciar a relação entre experiências e bem-estar. A literatura que analisa essa relação apresenta resultados contraditórios. Existem, por exemplo, estudos que identificam níveis maiores de ansiedade e depressão em grupos que relatam experiências anômalas e crenças paranormais (Maraldi, 2014), enquanto outros reportam as mesmas crenças (Haider, 2019) e outras experiências anômalas (Newberg & Waldman, 2019) se relacionando com a felicidade. Uma possibilidade para explicar os resultados contraditórios é justamente que, em geral, as pesquisas não separam a vivência em si da sua atribuição de sentido ou valência. Isso pode gerar vieses de acordo com a interpretação implícita na escala utilizada para medição (Irwin et al., 2013; Lange et al., 2019; Taves, 2020) ou de acordo com o contexto cultural que valoriza ou estigmatiza aquela experiência (Larøi et al., 2014). Algumas experiências podem estar relacionadas ao desenvolvimento espiritual ou pessoal (Benning et al., 2021; Khanna & Greyson, 2015; Royse & Badger, 2017), sendo que o bem-estar espiritual, bem como o significado de vida, podem ser considerados uma dimensão importante para a qualidade de vida (Ellison, 1983; Ryff & Keyes, 1995).

Além da potencial influência de perfis psicológicos, aspectos culturais também parecem ter relevância. Os pesquisadores geralmente assumem que experiências e interpretações são estáveis entre as culturas e que os itens são entendidos pelos entrevistados como os pesquisadores pretendiam. No entanto, há evidências crescentes que desafiam as suposições de universalidade cultural de experiências não-ordinárias, como elas podem ser experimentadas e especialmente como os indivíduos as entendem (ver especialmente o trabalho de Cassaniti & Luhrmann, 2012; Luhrmann et al., 2021; mas também Cohen et al., 2011). Embora esforços etnográficos tenham sido feitos para descrever grupos e/ou experiências específicas (Cohen, 2007; Cohen & Barrett, 2008; Schmidt, 2014, 2017), os dados quantitativos e psicométricos atualmente disponíveis não podem nos dizer a) como as pessoas em vários contextos culturais caracterizam as experiências não-ordinárias em relação às experiências comuns ou cotidianas, b) como essas experiências são

interpretadas; e c) como essas experiências podem impactar a saúde mental e bem-estar psicológico dos indivíduos.
O “Inventário de Experiências Não-Ordinárias” (INOE) desenvolvido por Taves e colaboradores (Taves, 2020; Taves & Barlev, 2022) foi desenvolvido para preencher as lacunas anteriormente citadas dos instrumentos pré-existentes. O INOE foca em aspectos subjetivamente reconhecíveis de experiências em vez de constructos pré-definidos, procura separar as características fenomenológicas das experiências em relação às interpretações culturalmente infundidas, e amplia a gama de experiências medidas incluindo algumas que podem ser relativamente comuns, mas que, ainda assim, podem se destacar para pessoas em algumas culturas e contextos. Essa lista mais abrangente, envolvendo experiências possivelmente positivas e negativas (desde vivenciar emoções positivas fortes até experiências de quase morte), amplia o escopo dos trabalhos anteriores, que focaram apenas em experiências clinicamente relevantes ou paranormais (American Psychiatric Association, 2013; Theresa M. Kelly, 2014; World Health Organization, 1993).

Hipótese:
A versão em português brasileiro do INOE irá apresentar validade.

Metodologia Proposta:
Adaptaremos e validaremos o Inventário de Experiências Não-ordinárias (INOE) para o contexto brasileiro e ampliaremos a seção de experiências dissociativas, bem como os componentes de atribuição do INOE considerando o contexto brasileiro. Primeiro, a versão em inglês (ver ANEXO I) será traduzida para o português do Brasil, usando uma abordagem de comitê paralelo (Harkness et al., 2010) que tem várias vantagens sobre o método tradicional de tradução-retro tradução (translation-back translation). Dada a natureza experiencial dos itens, é importante evitar interpretações específicas e preservar nuances dos itens originais. Por meio de dois comitês paralelos, serão obtidas duas traduções independentes, que serão comparadas por representantes de ambos os grupos. As divergências serão discutidas até que haja um consenso sobre a tradução de cada item.
Novos itens serão escritos com base em escalas de dissociação existentes, trabalho de campo anterior dos pesquisadores da equipe (por exemplo, Fischer & Kruekaew, 2020), bem como por meio de discussões de grupos focais com profissionais de saúde mental e religiosos. Esses itens serão validados com o mesmo protocolo do desenvolvimento original do INOE.

Usaremos o método de “Avaliação do Processo de Resposta” (RPE) (Wolf et al., 2019) para examinar a validade das experiências de cada item. O RPE utiliza paráfrases e entrevistas cognitivas para identificar se os respondentes da pesquisa interpretam um item como pretendido (Castillo-Díaz & Padilla, 2013).

O protocolo será realizado inteiramente de forma digital, através de questionários online divulgados através de um link. Ao acessar o link da pesquisa, o participante será apresentado ao TCLE e poderá aceitar ou não participar. Caso aceite participar da pesquisa, o participante será encaminhado ao questionário para avaliação da compreensão dos itens. O questionário será construído na plataforma FormR (Arslan et al., 2018). Essa plataforma é largamente utilizada em estudos científicos e possui métodos rigorosos de anonimato das informações pessoais dos participantes. Após consentir a participação na pesquisa, o participante é identificado por um código, não sendo possível identificá-lo individualmente.

Cada participante visualizará 5 itens do inventário relacionado a diferentes experiências, e deverá fornecer um exemplo para cada item, parafrasear o item e explicar suas respostas (ANEXO II). Seguindo o protocolo RPE original, uma equipe de codificadores avaliará se o item foi entendido como pretendido. A cada bloco de respostas (5 pessoas para um grupo de 5 itens), os avaliadores irão pontuar os itens de acordo com as respostas dos participantes em uma escala de 5 pontos (1 = Entendido a 5 = Não entendido). Caso algum dos itens seja considerado incompreendido, será modificado conforme necessário, e o processo continuará até que o item seja compreendido satisfatoriamente por uma amostra de 20 participantes (ver Seção 8.1 Análise dos dados).

Para o recrutamento dos participantes, utilizaremos método bola de neve com divulgação do link para questionário online em mídias sociais, na plataforma da saúde mental (saudemental.idor.org), empresas de painéis online que funcionam de acordo com as leis brasileiras (ex:NetQuest: www.netquest.com; Opinionbox: https://www.opinionbox.com/painel/), empresas de comunicação (ex: FutureHealth: futurehealth.cc) e contatos do IDOR. O recrutamento e participação dos participantes estará em conformidade com as diretrizes da Lei Geral de Proteção de Dados Pessoais (LGPD) e diretrizes internacionais de proteção de dados.

Critério de Inclusão:

Os critérios de inclusão serão participantes brasileiros letrados, com idades entre 18 e 90 anos.

Critério de Exclusão:

Serão excluídos aqueles participantes que não tiverem preenchido os questionários satisfatoriamente (respostas incompletas ou com indícios de desatenção).

Metodologia de Análise de Dados:
O processo de validação utilizará o método qualitativo RPE mencionado anteriormente. Membros treinados da equipe avaliarão as respostas dos participantes independentemente, considerando as interpretações para cada item. As respostas de cada participante a cada item da pesquisa serão avaliadas em uma escala de 5 pontos: 'Entendido' (1), 'Provavelmente compreendido' (2), ‘Sem informações suficientes' (3), 'Provavelmente não entendido' (4), ou 'Não entendido' (5).
Itens com classificação de 1 ou 5 indicarão respostas consistentes (1) ou inconsistentes (5) com a interpretação pretendida do item. Uma classificação de 2 ou 4 indicará que a resposta era consistente (2) ou inconsistente (4) com a interpretação pretendida, mas mais informações seriam necessárias para confirmar isso. Uma classificação de 3 indicará que a resposta não continha informações suficientes para avaliar se o item foi interpretado como pretendido. Se uma resposta não teve nenhuma relação com o que foi perguntado (por exemplo, respostas automatizadas ou de baixa qualidade), será atribuída uma classificação de 3*.
Itens classificados como 1 ou 2 por todos os codificadores receberão uma classificação geral de 'Entendido' (E), aqueles classificados como 4 ou 5 por todos os codificadores receberão uma classificação geral de 'Não entendido' (NE) e aqueles que foram classificados como 3 ou 3* por todos os codificadores manterão essa pontuação como sua classificação geral. Quando os codificadores discordarem sobre um item, será discutido em grupo para haver uma pontuação de consenso para essa resposta. Se forem identificados problemas de compreensão com os itens, esses serão revisados conforme necessário e uma nova rodada de coleta será feita com outros cinco participantes. Esse processo será repetido iterativamente até que a versão final de cada item seja avaliada pelo menos 20 vezes.
Cada item receberá uma pontuação de validação, indicando a proporção de respostas com uma classificação geral de ‘Entendido’, excluindo 3s e 3*s. Itens com uma pontuação de validação de pelo menos 80% serão considerados válidos provisoriamente. Os itens para os quais não obtermos uma pontuação de validação de pelo menos 80% serão eliminados da versão brasileira da INOE.
Desfecho Primário:
i) A maioria dos itens da versão em inglês de 42 itens apresentará validade na versão brasileira. Porém, espera-se que alguns itens não sejam replicados por questões linguísticas/culturais;
ii) Itens com conteúdo relacionado à cultura brasileira não contemplados na versão em inglês apresentarão evidência de validade.

Tamanho da Amostra no Brasil: 4.800

Informe o número de indivíduos abordados pessoalmente, recrutados, ou que sofrerão algum tipo de intervenção neste centro de pesquisa: 4.800

Objetivo da Pesquisa:
Objetivo Primário:

O objetivo primário do presente projeto é validar o INOE para o português brasileiro.

Objetivo Secundário:

Como objetivos secundários pretendemos expandir o instrumento desenvolvido anteriormente e capturar uma gama mais ampla de experiências voltadas para a cultura brasileira.

Avaliação dos Riscos e Benefícios:
Riscos:

Os riscos referentes ao estudo se referem àqueles relacionados ao responder perguntas abertas e fechadas durante preenchimento de questionários online, como invasão de privacidade, divulgação de dados confidenciais e tomar o tempo do participante. A equipe de pesquisa está atenta a estes riscos e trabalhando de forma a minimizá-los. Os dados serão armazenados em servidores mantidos pelo instituto, com equipe de TI dedicada a manutenção e alinhada com as principais diretrizes de segurança digital e LGPD. Os dados serão armazenados com segurança em um servidor da Amazon Web Services, Inc. (AWS) anonimizados e protegidos por criptografia. Vários requisitos de segurança estão implementados para garantir a confidencialidade e segurança dos dados, dentre eles: o MFA (múltiplo fator de autenticação), rastreamento CloudTrail de todas as contas, buckets privados, firewall habilitado em todos os acessos e Cognito (grupo de identidade de credenciais para AWS). Desta forma, a equipe de pesquisa tem o compromisso que os dados serão tratados de forma confidencial e as respostas individuais não serão divulgadas em nenhum caso. Adicionalmente, procuraremos minimizar ao máximo o tempo de resposta dos questionários, através do refinamento dos instrumentos da pesquisa. Caso os participantes desejarem, poderão retirar-se da pesquisa a qualquer momento.

Benefícios:

A pesquisa não trará nenhum benefício direto aos participantes, porém como benefício indireto, a participação ajudará na compreensão da validade do INOE para a população brasileira.

Comentários e Considerações sobre a Pesquisa:

Trata-se de um estudo observacional, unicêntrico, que objetiva traduzir e validar o Inventário de Experiências Não-ordinárias (INOE) para o contexto brasileiro. Sobre o conceito de "experiências não-ordinárias", os pesquisadores mencionam: "sensação de ser tocado ou ouvir uma voz quando ninguém está por perto, sentir que os próprios pensamentos parecem controlados externamente, ou ainda perder a percepção de tempo e espaço". Estas podem ser interpretadas como religiosas, anômalas ou patológicas, dependendo da origem cultural e das características individuais de quem as vivencia.

O INOE foi desenvolvido por Taves e cols. (2020) e consiste em uma listagem de frases descrevendo experiências não-ordinárias (p.ex., "I have had an experience of joy, ecstasy, or bliss that stood out from all other such experiences"), seguidas de perguntas acerca do (1) estado mental do participante durante a experiência, (2) do impacto desta vivência na vida do indivíduo, (3) da caracterização da experiência como religiosa / espiritual ou explicável pela ciência, (4) da possível razão pela qual a experiência ocorreu, e (5) de quem teria causado a vivência (divindades, espíritos etc.).

Todos os procedimentos ocorrerão exclusivamente em ambiente online. A pesquisa será divulgada em mídias digitais e o recrutamento utilizará método bola-de-neve. Serão incluídos participantes brasileiros letrados com idades entre 18 e 90 anos.

A pesquisa envolverá as seguintes etapas: (1) tradução por comitês paralelos; e (2) aplicação da versão traduzida em um grupo de 4800 participantes, visando à avaliação da validade de constructo por método semi-quantitativo (Avaliação do Processo de Resposta - RPE).

O orçamento da pesquisa totaliza R$ 295.418,49. O projeto possui financiamento interno aprovado pelo programa Ciência Pioneira (IDOR), conforme informado na carta de anuência da instituição, assinada pela presidente do IDOR. A referida presidente assinou também a seção correspondente ao Patrocinador na Folha de Rosto.

A pesquisa possui metodologia simples e adequada aos objetivos citados.

Considerações sobre os Termos de apresentação obrigatória:

Os seguintes documentos foram analisados nesta apreciação ética:

• 5_ID317_FR_RONALDFISHER_RJ_assinada.pdf – Folha de rosto assinada pelo pesquisador responsável e pela presidente do IDOR (instituição proponente e patrocinador principal).
• 2_ID317_INFRA_RONALDFISCHER_RJ_assinada.pdf – Declaração de infraestrutura institucional assinada pela presidente do IDOR.
• 3_ID317_TERMO_CONF_RONALDFISCHER_RJ_assinada.pdf – Termo de confidencialidade dos dados assinado pelo pesquisador principal e pela presidente do IDOR.
• Orcamento_Validacao_INOE.docx – Orçamento detalhado.
• 1_ID317_ANUENCIA_RONALDFISCHER_RJ_assinada.pdf – Termo de anuência da instituição assinado pelo pesquisador principal e pela presidente do IDOR.
• 4_ID317_VINCULO_RONALDFISCHER_RJ_assinada.pdf – Declaração de vínculo institucional assinado pelo pesquisador principal e pela presidente do IDOR.
• TCLE_Validacao_INOE.docx – TCLE digital.
• Cronograma_Validacao_INOE.docx – Cronograma do estudo.
• Submissao_Projeto_Validacao_INOE.docx – Projeto detalhado.

Recomendações:

Não há.

Conclusões ou Pendências e Lista de Inadequações:

Não foram observados óbices éticos à realização do presente projeto de pesquisa.

Considerações Finais a critério do CEP:

Este parecer foi elaborado baseado nos documentos abaixo relacionados:

Tipo Documento	Arquivo	Postagem	Autor	Situação
Informações Básicas do Projeto	PB_INFORMAÇÕES_BÁSICAS_DO_PROJETO_1998316.pdf	23/11/2022 11:13:58		Aceito
Projeto Detalhado	Submissao_Projeto_Validacao_INOE.	23/11/2022	Tiago Soares	Aceito

/ Brochura Investigador	docx	11:13:20	Bortolini	Aceito
Cronograma	Cronograma_Validacao_INOE.docx	23/11/2022 11:12:34	Tiago Soares Bortolini	Aceito
TCLE / Termos de Assentimento / Justificativa de Ausência	TCLE_Validacao_INOE.docx	23/11/2022 11:11:55	Tiago Soares Bortolini	Aceito
Outros	4_ID317_VINCULO_RONALDFISCHER_RJ_assinada.pdf	21/09/2022 09:56:50	Tiago Soares Bortolini	Aceito
Outros	1_ID317_ANUENCIA_RONALDFISCHER_RJ_assinada.pdf	21/09/2022 09:56:20	Tiago Soares Bortolini	Aceito
Orçamento	Orcamento_Validacao_INOE.docx	21/09/2022 09:51:54	Tiago Soares Bortolini	Aceito
Declaração de Pesquisadores	3_ID317_TERMO_CONF_RONALDFISCHER_RJ_assinada.pdf	21/09/2022 09:51:01	Tiago Soares Bortolini	Aceito
Declaração de Instituição e Infraestrutura	2_ID317_INFRA_RONALDFISCHER_RJ_assinada.pdf	21/09/2022 09:50:30	Tiago Soares Bortolini	Aceito
Folha de Rosto	5_ID317_FR_RONALDFISHER_RJ_assinada.pdf	21/09/2022 09:48:26	Tiago Soares Bortolini	Aceito

Situação do Parecer:
Aprovado

Necessita Apreciação da CONEP:
Não

RIO DE JANEIRO, 15 de Dezembro de 2022

Assinado por:
DENILSON CAMPOS DE ALBUQUERQUE (Coordenador(a))
