# Supplementary material for: Making sense of the extraordinary – An investigation of the content and prevalence of appraisals of nonordinary experiences
Source: PLoS One. 2026 Aug 5;21(8):e0353126. doi: 10.1371/journal.pone.0353126 (PMC13440842; doi:10.1371/journal.pone.0353126)
Supplement: Supplementary file 6 [file pone.0353126.s005.zip › CEP-material/PB_PARECER_CONSUBSTANCIADO_CEP_5819411_approved_translated.pdf]

Machine Translated by Google

SUBSTANTIATE OPINION OF THE CEP

RESEARCH PROJECT DATA

Search Title: Adaptation and validation of the Non-Ordinary Experiences Inventory into Brazilian Portuguese

Researcher: Ronald Fischer
Thematic Area:
Version: 1
CAAE: 65573322.6.0000.5249
Proposing Institution: D'OR INSTITUTE OF RESEARCH AND EDUCATION
Main Sponsor: D'OR INSTITUTE OF RESEARCH AND EDUCATION

OPINION DATA

Opinion Number: 5,819,411

Project Presentation:
This is the Project: Adaptation and validation of the Inventory of Non-Ordinary Experiences into Brazilian Portuguese with the following introduction:

Non-ordinary experiences, such as the sensation of being touched or hearing a voice when no one is around, feeling that one's thoughts seem externally controlled, or even losing one's perception of time and space, can be interpreted as religious, anomalous, or pathological, depending on the cultural background and individual characteristics of the person experiencing them (Acunzo et al., 2020; Cardeña et al., 2014; Larøi et al., 2014). Furthermore, people may interpret these experiences as very positive and highly meaningful or as disturbing, intriguing, or meaningless. In search of understanding and based on such interpretations, it is common to consult religious or medical experts. The type of specialist consulted can result in the spiritualization or medicalization of the experience (Moreira-Almeida et al., 2008).

Several studies indicate that there may also be differences in interpretation between researchers and study participants, even considering apparently similar experiences, but which can be interpreted as religious, spiritual, anomalous, paranormal and/or pathological (Luhrmann, 2017; McCauley & Graham, 2020; Schmidt, 2014, 2015). Despite the phenomenological overlap of such experiences (Hageman et al., 2011;

Machine Translated by Google

McCauley & Graham, 2020; Moreira-Almeida et al., 2006), many instruments use descriptions that define experiences a priori as religious, spiritual, anomalous, paranormal, and/or pathological.
Consequently, obtaining reliable estimates of the frequency of these experiences across the lifespan is difficult due to overlapping constructs, measurement designs, and question wording. Not only are similar items included in multiple constructs defined by the researcher under different labels, but many items are worded in ways that give them religious, paranormal, or psychopathological connotations (Taves, 2020), which can alter the response of participants who do not interpret the experience in the same way.

Interpretation also appears to influence the relationship between experiences and well-being. The literature analyzing this relationship presents contradictory results. For example, studies identify higher levels of anxiety and depression in groups that report anomalous experiences and paranormal beliefs (Maraldi, 2014), while others report the same beliefs (Haider, 2019) and other anomalous experiences (Newberg & Waldman, 2019) as being related to happiness. One possible explanation
The contradictory results are precisely due to the fact that, in general, research does not separate the experience itself from its attribution of meaning or valence. This can generate biases according to the interpretation implicit in the scale used for measurement (Irwin et al., 2013; Lange et al., 2019; Taves, 2020) or according to the cultural context that values or stigmatizes that experience (Larøi et al., 2014). Some experiences may be related to spiritual or personal development (Benning et al., 2021; Khanna & Greyson, 2015; Royse & Badger, 2017), and spiritual well-being, as well as meaning in life, can be considered an important dimension of quality of life (Ellison, 1983; Ryff & Keyes, 1995).
Beyond the potential influence of psychological profiles, cultural aspects also appear to be relevant. Researchers often assume that experiences and interpretations are stable across cultures and that items are understood by respondents as researchers intended. However, there is growing evidence challenging assumptions of the cultural universality of non-ordinary experiences, how they can be experienced, and especially how individuals understand them (see especially the work of Cassaniti & Luhrmann, 2012; Luhrmann et al., 2021; but also Cohen et al., 2011). Although ethnographic efforts have been made to describe specific groups and/or experiences (Cohen, 2007; Cohen & Barrett, 2008; Schmidt, 2014, 2017), currently available quantitative and psychometric data cannot tell us a) how people in various cultural contexts characterize non-ordinary experiences relative to ordinary or everyday experiences, b) how these experiences are

Machine Translated by Google

interpreted; and c) how these experiences can impact the mental health and psychological well-being of individuals.

The "Inventory of Non-Ordinary Experiences" (INOE), developed by Taves and colleagues (Taves, 2020; Taves & Barlev, 2022), was developed to fill the aforementioned gaps in existing instruments. The INOE focuses on subjectively recognizable aspects of experiences rather than predefined constructs, seeks to separate the phenomenological characteristics of experiences from culturally infused interpretations, and expands the range of experiences measured by including some that may be relatively common but may still be salient for people in some cultures and contexts. This more comprehensive list, encompassing potentially positive and negative experiences (from experiencing strong positive emotions to near-death experiences), broadens the scope of previous work, which focused solely on clinically relevant or paranormal experiences (American Psychiatric Association, 2013; Theresa M. Kelly, 2014; World Health Organization, 1993).

Hypothesis:
The Brazilian Portuguese version of the INOE will demonstrate validity.

Proposed Methodology:
We will adapt and validate the Inventory of Non-Ordinary Experiences (INOE) for the Brazilian context and expand the dissociative experiences section, as well as the attributional components of the INOE, considering the Brazilian context. First, the English version (see ANNEX I) will be translated into Brazilian Portuguese using a parallel committee approach (Harkness et al., 2010), which has several advantages over the traditional translation-back translation method. Given the experiential nature of the items, it is important to avoid specific interpretations and preserve the nuances of the original items. Through two parallel committees, two independent translations will be obtained, which will be compared by representatives of both groups. Disagreements will be discussed until a consensus is reached on the translation of each item.

New items will be written based on existing dissociation scales, previous fieldwork by the team's researchers (e.g., Fischer & Kruekaew, 2020), and focus group discussions with mental health professionals and religious leaders. These items will be validated using the same protocol as the original INOE development.

Machine Translated by Google

We will use the "Response Process Evaluation" (RPE) method (Wolf et al., 2019) to examine the validity of each item's experiences. RPE utilizes paraphrasing and cognitive interviews to identify whether survey respondents interpret an item as intended (Castillo-Díaz & Padilla, 2013).
The protocol will be conducted entirely digitally, through online questionnaires distributed via a link. Upon accessing the survey link, participants will be presented with the informed consent form and may choose to accept or decline participation. If they accept, they will be directed to the questionnaire to assess their understanding of the items.
The questionnaire will be developed on the FormR platform (Arslan et al., 2018). This platform is widely used in scientific studies and has rigorous methods for anonymizing participants' personal information. After consenting to participate in the survey, participants are identified by a code, making it impossible to identify them individually.

Each participant will view five inventory items related to different experiences and will be asked to provide an example for each item, paraphrase the item, and explain their responses (ANNEX II). Following the original RPE protocol, a team of coders will assess whether the item was understood as intended. For each block of responses (five people for a group of five items), the raters will score the items based on the participants' responses on a five-point scale (1 = Understood to 5 = Not Understood). If any of the items are considered misunderstood, they will be modified as necessary, and the process will continue until the item is satisfactorily understood by a sample of 20 participants (see Section 8.1 Data Analysis).

To recruit participants, we will use a snowball method, sharing the link to the online questionnaire on social media, the mental health platform (saudemental.idor.org), online panel companies that operate in accordance with Brazilian law (e.g., NetQuest: www.netquest.com; Opinionbox: https://www.opinionbox.com/painel/), communications companies (e.g., FutureHealth: futurehealth.cc), and IDOR contacts. Participant recruitment and participation will comply with the General Data Protection Law (LGPD) and international data protection guidelines.

Inclusion Criteria:

The inclusion criteria will be literate Brazilian participants, aged between 18 and 90 years old.

Exclusion Criteria:

Participants who have not completed the questionnaires satisfactorily (incomplete answers or with signs of inattention) will be excluded.

Machine Translated by Google

Data Analysis Methodology: The validation process will utilize the previously mentioned qualitative RPE method. Trained team members will evaluate participants' responses independently, considering their interpretations for each item. Each participant's responses to each survey item will be rated on a 5-point scale: 'Understood' (1), 'Probably understood' (2), 'Not enough information' (3), 'Probably not understood' (4), or 'Not understood' (5).

Items with a rating of 1 or 5 will indicate responses that were consistent (1) or inconsistent (5) with the intended interpretation of the item. A rating of 2 or 4 will indicate that the response was consistent (2) or inconsistent (4) with the intended interpretation, but more information would be needed to confirm this. A rating of 3 will indicate that the response did not contain enough information to assess whether the item was interpreted as intended. If a response was completely unrelated to what was asked (e.g., automated or low-quality responses), a rating will be assigned.

of 3*.

Items rated 1 or 2 by all coders will receive an overall rating of 'Understood' (E), those rated 4 or 5 by all coders will receive an overall rating of 'Not understood' (NE), and those rated 3 or 3* by all coders will retain that score as their overall rating. When coders disagree on an item, it will be discussed as a group to reach a consensus score for that response. If comprehension issues are identified with the items, they will be revised as needed, and a new round of data collection will be conducted with five additional participants. This process will be repeated iteratively until the final version of each item has been rated at least 20 times.

Each item will receive a validation score, indicating the proportion of responses with an overall rating of 'Understood,' excluding 3s and 3*s. Items with a validation score of at least 80% will be considered provisionally valid. Items for which we do not obtain a validation score of at least 80% will be eliminated from the Brazilian version of the INOE.

Primary Outcome:

i) Most items in the 42-item English version will be valid in the Brazilian version. However, it is expected that some items will not be replicated due to linguistic/cultural issues; ii) Items with content related to Brazilian culture not covered in the English version

will be

evidence of validity.

Sample Size in Brazil: 4,800

Machine Translated by Google

Report the number of individuals personally approached, recruited, or who will undergo some type of intervention at this research center: 4,800

Research Objective:

Primary Objective:

The primary objective of this project is to validate the INOE for Brazilian Portuguese.

Secondary Objective:

As secondary objectives, we intend to expand the previously developed instrument and capture a wider range of experiences focused on Brazilian culture.

Risk and Benefit Assessment:

Risks:

The risks associated with the study include those associated with answering open- and closed-ended questions while completing online questionnaires, such as invasion of privacy, disclosure of confidential data, and wasting participants' time. The research team is aware of these risks and is working to minimize them. The data will be stored on servers maintained by the institute, with a dedicated IT team for maintenance and aligned with the main digital security and LGPD guidelines. The data will be securely stored on an Amazon Web Services, Inc. (AWS) server, anonymized and protected by encryption. Several security requirements are implemented to ensure data confidentiality and security, including: MFA (multi-factor authentication), CloudTrail tracking of all accounts, private buckets, a firewall enabled on all accesses, and Cognito (an identity pool of credentials for AWS). Therefore, the research team is committed to ensuring that the data will be treated confidentially and that individual responses will not be accessed.

disclosed under no circumstances. Additionally, we will strive to minimize questionnaire response times by refining the survey instruments. Participants may withdraw from the survey at any time if they so wish.

Benefits:

Machine Translated by Google

The research will not bring any direct benefit to the participants, but as an indirect benefit, participation will help in understanding the validity of the INOE for the Brazilian population.

Comments and Considerations on the Research:

This is a single-center, observational study that aims to translate and validate the Inventory of Non-Ordinary Experiences (INOE) for the Brazilian context. Regarding the concept of "non-ordinary experiences," the researchers mention: "the sensation of being touched or hearing a voice when no one is around, feeling that one's thoughts seem externally controlled, or even losing one's perception of time and space." These can be interpreted as religious, anomalous, or pathological, depending on the cultural background and individual characteristics of those experiencing them.

The INOE was developed by Taves et al. (2020) and consists of a list of sentences describing non-ordinary experiences (e.g., "I have had an experience of joy, ecstasy, or bliss that stood out from all other such experiences"), followed by questions about (1) the participant's mental state during the experience, (2) the impact of this experience on the individual's life, (3) the characterization of the experience as religious/ spiritual or explainable by science, (4) the possible reason why the experience occurred, and (5) who could have caused the experience (deities, spirits, etc.).

All procedures will take place exclusively online. The survey will be publicized on digital media, and recruitment will use a snowball method. Brazilian participants will be included.
literate people aged between 18 and 90 years.

The research will involve the following steps: (1) translation by parallel committees; and (2) application of the translated version to a group of 4,800 participants, aiming to assess construct validity using a semi-quantitative method (Response Process Evaluation - RPE).

The research budget totals R$295,418.49. The project has internal funding approved by the Pioneering Science Program (IDOR), as stated in the institution's letter of approval, signed by the IDOR president. The president also signed the Sponsor section in the

Title Page.

Address: Diniz Cordeiro Street, 30 - 2nd Floor
Neighborhood: Botafogo **ZIP Code:** 22.281-100
State: RJ **Municipality:** RIO DE JANEIRO
Telephone: (21)3883-6013 **Fax:** (21)3883-6013 **Email:** cep.idor@idor.org

Machine Translated by Google

The research has a simple methodology and is appropriate for the objectives mentioned.

Considerations on Mandatory Presentation Terms:

The following documents were analyzed in this ethical assessment:

•5_ID317_FR_RONALDFISHER_RJ_assinada.pdf – Cover page signed by the principal investigator and the president of IDOR (proposing institution and main sponsor). •2_ID317_INFRA_RONALDFISCHER_RJ_assinada.pdf – Declaration of institutional infrastructure signed by the president of IDOR. •3_ID317_TERMO_CONF_RONALDFISCHER_RJ_assinada.pdf – Data confidentiality agreement signed by the principal investigator and the president of IDOR.

•Orcamento_Validacao_INOE.docx – Detailed budget.
•1_ID317_ANUENCIA_RONALDFISCHER_RJ_assinada.pdf – Institutional consent form signed by the principal investigator and the president of IDOR. •4_ID317_VINCULO_RONALDFISCHER_RJ_assinada.pdf – Declaration of institutional affiliation signed by the principal investigator and the president of IDOR. •TCLE_Validacao_INOE.docx – Digital TCLE. •Cronograma_Validacao_INOE.docx – Study schedule.
•Submissao_Projeto_Validacao_INOE.docx – Detailed project.

Recommendations:

There is none.

Conclusions or Pending Issues and List of Inadequacies: No ethical obstacles were observed in carrying out this research project.

Final Considerations at the discretion of the CEP:

This opinion was prepared based on the documents listed below:

Type Document File PB	INFORMAÇÕES_BÁSICAS_DO_P	Post	Author	Situation
Detailed Project Submission_Project_Validation_INOE.	Basic Project Information ROJETO_1998316.pdf	11/23/2022 11:13:58 AM		Accepted
		11/23/2022	Tiago Soares	Accepted

Address: Diniz Cordeiro Street, 30 - 2nd Floor
Neighborhood: Botafogo **ZIP Code:** 22.281-100
State: RJ **Municipality:** RIO DE JANEIRO
Telephone: (21)3883-6013 **Fax:** (21)3883-6013 **Email:** cep.idor@idor.org

Machine Translated by Google

/ Brochure Researcher	docx	11:13:20	Bortolini	Accepted
Timeline	Schedule_Validation_INOE.docx	23/11/2022 11:12:34	Tiago Soares Bortolini	Accepted
TCLE / Terms of Assent / Justification of Absence	TCLE_Validacao_INOE.docx	23/11/2022 11:11:55	Tiago Soares Bortolini	Accepted
Others	4_ID317_VINCULO_RONALDFISCHER_RJ_assinada.pdf	21/09/2022 09:56:50	Tiago Soares Bortolini	Accepted
Others	1_ID317_ANUENCIA_RONALDFISCHER_RJ_assinada.pdf	21/09/2022 09:56:20	Tiago Soares Bortolini	Accepted
Budget	Orcamento_Validacao_INOE.docx	21/09/2022 09:51:54	Tiago Soares Bortolini	Accepted
Declaration of Researchers	3_ID317_TERMO_CONF_RONALDFISCHER_RJ_assinada.pdf	21/09/2022 09:51:01	Tiago Soares Bortolini	Accepted
Declaration of Institution and Infrastructure	2_ID317_INFRA_RONALDFISCHER_RJ_assinada.pdf	21/09/2022 09:50:30	Tiago Soares Bortolini	Accepted
Title Page	5_ID317_FR_RONALDFISHER_RJ_assinada.pdf	09/21/2022 09:48:26	Tiago Soares Bortolini	Accepted

Opinion Status:

Approved

Needs CONEP's Appreciation:

No

RIO DE JANEIRO, December 15, 2022

Signed by:
DENILSON CAMPOS DE ALBUQUERQUE
(Coordinator)
