# Supplementary material for: Making sense of the extraordinary – An investigation of the content and prevalence of appraisals of nonordinary experiences
Source: PLoS One. 2026 Aug 5;21(8):e0353126. doi: 10.1371/journal.pone.0353126 (PMC13440842; doi:10.1371/journal.pone.0353126)
Supplement: Supplementary file 6 [file pone.0353126.s005.zip › CEP-material/PB_PARECER_CONSUBSTANCIADO_CEP_7358228-aprovado.pdf]

PARECER CONSUBSTANCIADO DO CEP

DADOS DO PROJETO DE PESQUISA

Título da Pesquisa: Perfil psicológico de Experiências Não-Ordinárias (NOE) e suas associações com diferenças individuais no contexto brasileiro.

Pesquisador: Ronald Fischer

Área Temática:

Versão: 2

CAAE: 85708425.3.0000.5249

Instituição Proponente: INSTITUTO D'OR DE PESQUISA E ENSINO

Patrocinador Principal: INSTITUTO D'OR DE PESQUISA E ENSINO
INSTITUTO D'OR DE PESQUISA

DADOS DO PARECER

Número do Parecer: 7.358.228

Apresentação do Projeto:

As informações contidas nas seções "Apresentação do projeto", "Objetivo da pesquisa", "Avaliação de riscos e benefícios" foram extraídas do documento PB_INFORMAÇÕES_BÁSICAS_DO_PROJETO_2481019.

INTRODUÇÃO:

Experiências anômalas, como a sensação de ser tocado ou ouvir uma voz quando ninguém está por perto, sentir que os próprios pensamentos parecem controlados externamente, ou ainda perder a percepção de tempo e espaço, podem ser interpretadas como religiosas, anômalas ou patológicas, dependendo da origem cultural e das características individuais de quem as vivencia (Acunzo et al., 2020; Cardeña et al., 2014). Além disso, pessoas podem interpretar essas experiências como muito positivas e altamente significativas ou como perturbadoras, intrigantes ou sem sentido. Em busca de compreensão e em função de tais interpretações, é comum consultar especialistas religiosos ou médicos. O tipo de especialista consultado pode resultar na espiritualização ou medicalização da experiência (Moreira-Almeida et al., 2008). A interpretação também parece influenciar a relação entre experiências e bem-estar. A literatura que analisa essa relação apresenta resultados contraditórios. Uma possibilidade para explicar

esses resultados é justamente que, em geral, as pesquisas não separam a vivência em si da sua atribuição de sentido ou valência. Isso pode gerar vieses de acordo com a interpretação implícita na escala utilizada para medição (Irwin et al., 2013; Lange et al., 2019; Taves, 2020) ou de acordo com o contexto cultural que valoriza ou estigmatiza aquela experiência. Nesse sentido, O Inventário de Experiências Não-Ordinárias (INOE) foi desenvolvido por Taves e colaboradores (Taves, 2020; Taves & Barlev, 2022) para preencher as lacunas e vieses observados nos instrumentos pré-existentes. O INOE foca em aspectos subjetivamente reconhecíveis de experiências em vez de constructos pré-definidos, procurando separar as características fenomenológicas das experiências de interpretações culturalmente infundidas. Assim, o inventário amplia a gama de experiências medidas incluindo algumas que podem ser relativamente comuns, mas que, ainda assim, podem se destacar para pessoas em algumas culturas e contextos. Essa lista mais abrangente, envolvendo experiências possivelmente positivas e negativas (desde vivenciar emoções positivas fortes até experiências de quase morte), amplia o escopo dos trabalhos anteriores, que focaram apenas em experiências clinicamente relevantes ou paranormais (American Psychiatric Association, 2013; Kelly, 2014; World Health Organization, 1993). O uso do INOE na coleta de dados oferecerá maior flexibilidade ao projeto, uma vez que o instrumento não se propõe a mensurar um construto único (Taves et al., 2019), sendo possível optar por aplicar apenas parte dos seus itens - isto é, apenas as experiências anômalas mais pertinentes ao estudo. O mesmo vale para os itens adicionais relativos à interpretação atribuída às experiências - contexto, espiritualidade, agente causador, percepção de controle, impacto na vida, etc. É importante investigar a frequência e distribuição de NOEs em amostras representativas. Quão comuns são os diferentes tipos de NOEs, dadas as estimativas generalizadas de menos de 10% a cerca de 80% em estudos que usaram escalas de experiências selecionadas orientadas por pesquisadores? A descrição da frequência e distribuição de NOEs em uma população é de grande interesse para diferentes estudiosos, principalmente médicos e profissionais de saúde mental, mas também antropólogos e psicólogos interessados no funcionamento da mente (Luhrmann et al., 2015a). Uma segunda questão é a possibilidade de explorar o agrupamento dessas experiências e suas avaliações. Como mencionamos acima, cada item é considerado como um fenômeno único, semelhante a uma variável latente na psicologia. Ao mesmo tempo, é possível que algumas experiências coocorram na população em geral, seja por possíveis vias neurobiológicas comuns (Cohen & Barrett, 2008; Cristofori et al., 2016; Kapogiannis et al. , 2009) ou em virtude da socialização cultural (por exemplo, Luhrmann et al., 2015b; Maraldi & Krippner, 2019) . Ao

explorar o agrupamento dessas experiências, é possível ter novos insights sobre a estrutura da mente humana e experiências não-ordinárias, que serão informativas para a compreensão da psicopatologia e do bem-estar psicológico. A fim de compreender os mecanismos psicológicos subjacentes às experiências não ordinárias, muitos autores têm trabalhado no sentido de identificar traços e características individuais capazes de melhor predizê-las. Irwin (2009) observa que alguns estilos de atribuição de significado, como atribuições e crenças paranormais, são mais comumente observados em pessoas que relatam experiências não ordinárias. Junto a isso, alguns aspectos da personalidade e da cognição parecem se correlacionar simultaneamente com a vivência de experiências não ordinárias e com estilos de atribuição orientados ao paranormal, dentre os quais se destacam: esquizotipia, ideação mágica, anomalias sensoriais (Claridge & Beech, 1995; Irwin, 2009; Wolfradt, 1999), raciocínio emocional, suspensão do teste de realidade (Irwin, 2009), traços específicos da personalidade (e.g., extroversão: Rattet, 2001; abertura: McCrae, 1994), absorção (Tellegen & Atkinson, 1974), propensão à fantasia (Woody & Barnier, 2008) e transliminaridade (Sherwood & Milner, 2005; Thalbourne & Houran, 2000). Para além das particularidades da personalidade, ao mesmo tempo que indissociável destas, a história de vida e os eventos externos aos quais o indivíduo é exposto também se correlacionam com relatos de experiências não ordinárias (Cardeña, Lynn & Krippner, 2017; Parra, 2019). A associação entre eventos traumáticos ocorridos no período da infância e da adolescência e indicadores de sofrimento mental na vida adulta é consolidada na literatura psiquiátrica. Sabe-se que a exposição a traumas agudos ou crônicos está ligada à depressão (Mandelli, Petrelli & Serretti, 2015), transtorno bipolar (Aas et al., 2016), transtornos de personalidade (Cummings, Berkowitz & Scribano, 2012), transtorno do estresse pós-traumático (Yehuda, Halligan & Grossman, 2001) e prejuízos em competências cognitivas e socioemocionais (Enoch, 2011). A associação entre traumas na infância e o desenvolvimento de estados dissociativos e relatos de experiências não ordinárias vem sendo amplamente explorada na literatura (Lawrence et al., 1995; McLewin & Muller, 2005; Parra, 2019; Rabeyron & Watt, 2010; Toutountzidis et al., 2022), e alguns autores sugerem que esses processos podem apresentar função adaptativa em resposta às experiências adversas (Bailey & Brand, 2017; Marks, 2020) e atuar na atenuação de sintomas psicopatológicos (Corso et al., 2023). Especula-se que tanto as crenças em fenômenos sobrenaturais quanto as experiências não ordinárias proporcionem uma via de simbolização e produção de sentido diante de experiências de adversidade, de forma que alguma interpretação sobre o evento traumático se torne possível (Lasikiewicz, 2016; Park, 2010; Rabeyron & Loose, 2015). Contudo, a potencial

função adaptativa das experiências não ordinárias não é clara, e compreende-se que estas também podem apresentar uma dimensão problemática para a saúde mental, contribuindo para o afloramento de emoções negativas, estigma e isolamento social (Rabeyron, 2022) ou podem funcionar como forma protetor (Lasikiewicz, 2016; Park, 2010; Rabeyron & Loose, 2015). Como visto anteriormente, é possível que tanto a qualidade fenomenológica das experiências anômalas quanto a forma como são interpretadas subjetivamente e culturalmente influenciem a força e a direção do seu efeito sobre a saúde mental (Hartle et al., 2023; Maraldi et al., 2023). Estudos descrevem que interpretações de causalidade atribuída a agentes externos, assim como uma maior tentativa de controle sobre o evento e sensações de alteração no funcionamento cognitivo estão associadas a impactos emocionais negativos, enquanto experiências interpretadas sob um viés espiritual, com maior percepção de controle e senso de aceitabilidade e suporte social tendem a produzir menos sofrimento (Brett et al., 2014). Nesse sentido, valendo-se de instrumentos validados para o contexto brasileiro, este projeto de pesquisa busca elucidar a complexa associação entre as experiências não ordinárias, características da personalidade e cognição, o histórico de eventos adversos na vida e indicadores de saúde mental. Um aspecto de singular importância será a investigação voltada tanto para a dimensão fenomenológica da experiência quanto para a sua interpretação subjetiva - a forma como os indivíduos atribuem sentido e valor aos fenômenos anômalos - e como essa interpretação se relaciona com os desfechos de bem-estar psicológico. Dessa forma, será possível trazer avanços na compreensão de mecanismos psicológicos relacionados a experiências não ordinárias, na distinção entre o não ordinário e o psicopatológico, e na redução do estigma associado.

HIPÓTESE:

Hipóteses do Estudo:

¿ Hipótese 1: A prevalência e distribuição de experiências não-ordinárias na população brasileira será heterogênea de acordo com fatores sociodemográficos, assim como de acordo com a afiliação religiosa.

¿ Hipótese 2: Afiliações religiosas que incentivam experiências não-ordinárias apresentarão uma frequência maior de experiências não-ordinárias.

¿ Hipótese 3: Indivíduos com um índice maior de fantasia e abertura relatarão mais experiências não ordinárias.

¿ Hipótese 4: Indivíduos com um índice maior de extroversão relatarão mais experiências não

ordinárias.

¿ Hipótese 5: Indivíduos com um índice maior de necessidade de certeza relatarão menos experiências não ordinárias.

¿ Hipótese 6: Indivíduos mais jovens relatarão mais experiências não ordinárias.

¿ Hipótese 7 Indivíduos inseridos em contextos religiosos relatarão mais experiências não ordinárias.

¿ Hipótese 8: Indivíduos com histórico de experiências traumáticas relatarão mais experiências não ordinárias.

¿ Hipótese 9: A atribuição de significado sobrenatural à experiência não ordinária se correlacionará com traços de personalidade, valores, crenças e formas de pensamento.

METODOLOGIA PROPOSTA:

Coleta de dados: Para o recrutamento, utilizaremos o método bola de neve e empresas de painéis online que funcionam de acordo com as leis brasileiras (por exemplo, www.netquest.com; https://www.opinionbox.com/painel/; https://www.qualtrics.com/pt-br/) e contatos da equipe de pesquisa. O recrutamento e participação dos participantes estará em conformidade com as diretrizes da Lei Geral de Proteção de Dados Pessoais (LGPD) e diretrizes internacionais de proteção de dados. A coleta de dados será realizada com amostra representativa da população brasileira, constituída de participantes advindos de painéis online. A população alvo do estudo corresponde à população brasileira adulta. A amostra será distribuída proporcionalmente por cotas de idade, sexo, região e nível socioeconômico. Análise dos dados: Inicialmente, utilizaremos análises estatísticas descritivas de frequências, médias e variabilidade usando o pacote psych utilizando a linguagem R. Posteriormente, utilizaremos Análise Exploratória de Gráficos (EGA) (28) para identificar possíveis agrupamentos das experiências com outras variáveis psicológicas. Serão também realizadas análises de regressão linear através do mesmo software, utilizando a linguagem R. Instrumentos: Para a construção de um perfil psicológico abrangente dos participantes da pesquisa, serão utilizados diversos testes psicométricos amplamente reconhecidos na literatura científica e validados para o contexto brasileiro. Esses instrumentos avaliam diferentes dimensões psicológicas e características individuais, como personalidade, cognição, emoções, bem-estar e experiências de vida. Por exemplo, testes de personalidade incluem o Inventário dos Cinco Grandes Fatores de Personalidade (John, 1991), que mede os cinco grandes traços de personalidade, e o Inventário de Personalidade IPO (Lenzenweger, 2001), que explora aspectos de organização da

personalidade. Para avaliar crenças e atitudes, serão aplicados a Escala de Dogmatismo de Rokeach (Rokeach, 1960) e a Escala de Sentido de Coerência (Antonovsky, 1993). No campo das experiências de vida, instrumentos como o Questionário de Experiências Adversas na Infância ¿ ACE (Felitti et al., 1998) e o Questionário de Trauma na Infância ¿ CTQ (Bernstein et al., 2003) analisam o impacto de eventos adversos na infância. Outros testes, como o Indicador de Condição do Sono (Espie et al., 2014), investigam a qualidade do sono, enquanto escalas como o Breve COPE (Carver, 1997) avaliam estratégias de enfrentamento diante de situações desafiadoras. Além disso, o Questionário de Vivacidade da Imagem Visual ¿ VVIQ (Marks, 1973) mede a vivacidade das imagens mentais visuais, e a Escala de Controle Fenomenológico (Lush, 2021) examina a capacidade de controlar experiências perceptivas A lista de instrumentos completa está disponível no anexo A e formará o banco de itens a serem apresentados aos participantes usando o método Massive Missing Completely at Random - MMCAR (Reveille, 2024), que permite medir um grande número de construtos em amostras representativas para cobrir um escopo comportamental abrangente. O método é parecido com uma série de radiotelescópios que individualmente se limitam a uma cobertura individual com muitos ruídos, mas um conjunto de milhares de radiotelescópios direcionados para todas as direções geográficas resulta em uma imagem nítida do céu completo. Apresentamos aleatoriamente itens traduzidos e validados para o português. A estimativa de tempo para o voluntário participar na pesquisa será de 15 minutos. Exemplos de itens podem ser vistos no anexo B.

CRITÉRIO DE INCLUSÃO:
Brasileiros acima de 18 anos, alfabetizados.
CRITÉRIO DE EXCLUSÃO:
¿ Indivíduos sem condições plenas de compreender e/ou responder aos instrumentos utilizados na coleta de dados.
¿ Falha em preencher o TCLE.
¿ Preenchimento indevido ou incompleto de um ou mais instrumentos do estudo.

METODOLOGIA DE ANÁLISE DE DADOS:
Inicialmente, utilizaremos análises estatísticas descritivas de frequências, médias e variabilidade usando o pacote psych utilizando a linguagem R. Posteriormente, utilizaremos Análise Exploratória de Gráficos (EGA) (28) para identificar possíveis agrupamentos das experiências com outras variáveis psicológicas. Serão também realizadas análises de regressão

linear através do mesmo software, utilizando a linguagem R. Os dados serão armazenados em servidores mantidos pelo instituto, com equipe de TI dedicada a manutenção e alinhada com as principais diretrizes de segurança digital e LGPD. Os dados serão armazenados com segurança em um servidor da Amazon Web Services, Inc. (AWS) anonimizados e protegidos por criptografia. Vários requisitos de segurança estão implementados para garantir a confidencialidade e segurança dos dados, dentre eles: o MFA (múltiplo fator de autenticação), rastreamento CloudTrail de todas as contas, buckets privados, firewall habilitado em todos os acessos e Cognito (grupo de identidade de credenciais para AWS).

DESFECHO PRIMÁRIO:
Esperamos um panorama complexo de associações entre experiências não ordinárias e fatores psicológicos.
DESFECHO SECUNDÁRIO:
De uma forma positiva, esperamos que pessoas com um índice maior de fantasia e abertura relatarão mais experiências não ordinárias. Também há a expectativa de que o apoio social diminua qualquer efeito negativo destas experiências não ordinárias sobre a saúde mental.

Tamanho da Amostra no Brasil: 20.000

Haverá uso de fontes secundárias de dados (prontuários, dados demográficos, etc)?
Não

Informe o número de indivíduos abordados pessoalmente, recrutados, ou que sofrerão algum tipo de intervenção neste centro de pesquisa: 20.000

Objetivo da Pesquisa:
OBJETIVO PRIMÁRIO:
Explorar a prevalência, as formas de agrupamento e os correlatos psicológicos das experiências não ordinárias, de forma a identificar possíveis preditores que se relacionam com uma maior probabilidade de vivenciá-las.
OBJETIVO SECUNDÁRIO:
¿ Entender a prevalência de experiências não-ordinárias usando amostras representativas que

possibilitem relatar a distribuição de experiências não ordinárias no Brasil na população em geral e em diferentes afiliações religiosas.
¿ Investigar a existência de perfis psicológicos que aumentam a probabilidade de relatar experiências não ordinárias.
¿ Explorar a clusterização de experiências não-ordinárias e seus correlatos psicológicos.
¿ Testar se experiências não-ordinárias atuam como fatores de proteção contra os efeitos do trauma sobre a saúde mental na vida adulta.

Avaliação dos Riscos e Benefícios:
RISCOS:
Estudos acerca dos aspectos éticos da pesquisa com enfoque no trauma sugerem que o uso de instrumentos de autorrelato ou entrevistas está associado a sensações de desconforto momentâneo em uma pequena parcela de participantes (Ferrier-Auerbach et al., 2009; Jaffe et al., 2015; Yeater et al., 2012). Por outro lado, o desconforto relatado pelos participantes é leve e é excedido pela percepção de benefício em participar das pesquisas, sendo considerada, em geral, uma experiência positiva (Jaffe et al., 2015, Newman & Kaloupek, 2004). No momento da coleta de dados, realizada através da plataforma FormR, o participante deverá ser avisado previamente sobre o teor do questionário e a possibilidade deste provocar desconforto emocional. Neste momento, será enfatizada a possibilidade de o participante não responder a quaisquer perguntas e interromper a participação na pesquisa quando desejar. Outros aspectos considerados no momento inicial do questionário serão: a explicação detalhada dos propósitos do estudo e seus possíveis benefícios diretos e indiretos, a indicação do tempo estimado para completar o questionário e o uso de linguagem empática e não-julgadora. Ao final do questionário, haverá a inclusão de informações sobre os impactos do trauma na saúde mental e a possibilidade de se buscar ajuda profissional quando necessário, junto a informações sobre serviços de saúde mental na RAPS e a possibilidade de o participante oferecer feedback aos pesquisadores.

BENEFÍCIOS:
Benefícios diretos: O preenchimento dos questionários proporciona aos participantes a possibilidade de refletir sobre como suas experiências e característica pessoais, além de como tais podem ter impactado bem-estar atual, auxiliando na identificação da necessidade de apoio. As informações disponibilizadas ao final dos questionários também poderão contribuir

Endereço: Rua Diniz Cordeiro, 30 - 2º Andar
Bairro: Botafogo **CEP:** 22.281-100
UF: RJ **Município:** RIO DE JANEIRO
Telefone: (21)3883-6013 **Fax:** (21)3883-6013 **E-mail:** cep.idor@idor.org

com a desestigmatização de experiências e traumas, de maneira a promover uma percepção mais compassiva e de não julgamento acerca do tema. Benefícios indiretos: Os resultados do estudo fornecerão dados sobre a prevalência de experiências não-ordinárias no Brasil, sua relação com variáveis em saúde mental e características individuais, contribuindo para a psicologia no âmbito teórico e clínico, e também para a saúde pública.

Comentários e Considerações sobre a Pesquisa:

A apreciação ética anterior destacou as pendências abaixo que foram respondidas pelo pesquisador:

1. No projeto, devem ser detalhados todos os procedimentos que serão realizados na pesquisa. Por exemplo, deve ser esclarecido quais procedimentos serão realizados de modo remoto e presencial, e onde estas etapas serão executadas (item 3.4 da Norma Operacional n. 001/2013).

RESPOSTA DO PESQUISADOR: O estudo será conduzido integralmente de forma online nesta etapa inicial. A coleta de dados presencial será considerada em uma fase futura, após a análise dos resultados obtidos online e a identificação e esclarecimento de pontos-chave levantados durante essa análise. Essa estratégia permite otimizar o planejamento e assegurar maior eficiência na condução do estudo. O projeto foi ajustado para contemplar exclusivamente a versão online nesta etapa. Caso a coleta de dados presencial venha a ser realizada no futuro, será submetida a um novo processo de autorização pelo CEP.

PARECER DO RELATOR: Pendência atendida.

2. No TCLE, há campos não preenchidos e destacados em amarelo, como o nome da instituição, título do projeto e data da versão. Ademais, este termo deve ser redigido com linguagem clara e acessível aos participantes da pesquisa (item IV.1b da Resolução CNS n. 466/2012). Assim, as seções ¿Objetivos do Estudo¿ e ¿Justificativa¿ do TCLE devem ser revisadas para que se adequem à normativa citada.

RESPOSTA DO PESQUISADOR: Preenchemos os campos abertos, incluindo o nome da

instituição, o título do projeto e a data da versão. Além disso, revisamos as seções de objetivos e justificativa, alinhando-as às diretrizes apresentadas no item IV.1b da Resolução CNS n.º 466/2012.

PARECER DO RELATOR: Pendência atendida.

3. Além disso, no TCLE - versão presencial, deverá estar explícita a garantia de ressarcimento das despesas tidas pelos participantes da pesquisa e dela decorrentes (item IV.3g da Portaria CNS n. 466/2012).

RESPOSTA DO PESQUISADOR: Conforme mencionado no item 1 acima, esclarecemos que a coleta de dados será realizada de forma online nesta etapa da pesquisa.

PARECER DO RELATOR: A versão presencial do TCLE foi retirada da Plataforma Brasil, restando somente a versão remota. Pendência atendida.

4. Deve ser submetida carta de outorga ou similar contendo garantia de financiamento do projeto pelo patrocinador. Sempre enviar o orçamento detalhado.

RESPOSTA DO PESQUISADOR: Acrescentamos uma carta da Iniciativa Ciência Pioneira/IDOR, que detalha a ciência do projeto e o apoio oferecido pela pesquisa em curso. Também anexamos o contrato firmado entre o Instituto D¿Or de Pesquisa e Ensino e a Fundação John Templeton, que apresenta o orçamento disponível para os fins desta pesquisa.

PARECER DO RELATOR: Foi inserida carta assinada pelo diretor científico da Ciência Pioneira/IDOR e a explicação de que esta iniciativa será responsável pelo custeio de uma bolsa de pós-doutorado. O contrato assinado entre IDOR e Fundação John Templeton custeará as demais despesas do projeto, conforme explicitado pelo pesquisador. Pendência atendida.

5. Há documentos submetidos à Plataforma Brasil que não contêm assinatura do pesquisador ou do responsável institucional, a saber: 6_Termo_Cobertura_Custos.doc, 07_Termo_Utilizacao_Dados.doc. Versões assinadas destes documentos deverão ser enviadas pela Plataforma.

RESPOSTA DO PESQUISADOR: Nossa equipe pede desculpas por ter submetido inicialmente um termo sem a assinatura do responsável. Enviamos agora todos os documentos recebidos pelo NAPE devidamente assinados pelos responsáveis, conforme exigido pela Plataforma Brasil.

PARECER DO RELATOR: Pendência atendida.

Considerações sobre os Termos de apresentação obrigatória:

Os seguintes documentos foram analisados nesta apreciação ética:

¿ 01_Carta_Anuencia_Infra.pdf
¿ 01_Carta_Anuencia_Infra.pdf
¿ 02_Declaracao_Vinculo_Pesquisador.pdf
¿ 02_Declaracao_Vinculo_Pesquisador.pdf
¿ 03_Declaracao_confidencialidade_privacidade.pdf
¿ 03_Declaracao_confidencialidade_privacidade.pdf
¿ 04_Compromisso_Resolucoes.docx
¿ 04_Compromisso_Resolucoes.docx
¿ 05_Delineamento_Amostras.docx
¿ 05_Delineamento_Amostras.docx
¿ 06_Termo_Cobertura_Custos.doc
¿ 06_Termo_Cobertura_Custos.doc
¿ 06_Termo_Cobertura_Custos.pdf
¿ 07_Termo_Utilizacao_Dados.doc
¿ 07_Termo_Utilizacao_Dados.doc
¿ 07_Termo_Utilizacao_Dados.pdf
¿ 08_Declaracao_Infraestrutura.pdf
¿ 08_Declaracao_Infraestrutura.pdf
¿ 09_Declaracao_Infraestrutura_RJ.pdf
¿ 09_Declaracao_Infraestrutura_RJ.pdf
¿ 10a_INOE_TCLE_Presencial_nov26_2024.docx
¿ 10a_INOE_TCLE_Presencial_nov26_2024.docx
¿ 10a_INOE_TCLE_Presencial_nov26_2024.docx

¿ 10b_INOE_TCLE_Online.docx
¿ 10b_INOE_TCLE_Online.docx
¿ 10b_INOE_TCLE_Online.docx
¿ 10b_INOE_TCLE_Online_rev.docx
¿ 12__Detalhado.docx
¿ 12__Detalhado.docx
¿ 13_PerfilpsicologicoINOE_Orcamento.docx
¿ 13_PerfilpsicologicoINOE_Orcamento.docx
¿ 14_62651_Instituto_DOr_Countersigned_Agreement.pdf
¿ 15_carta_resposta_jan22.docx
¿ 16_Declaracao_NOEs_Ron_2025_vf.pdf
¿ Anexo_A_e_B_v2.docx
¿ Anexo_A_e_B_v2.docx
¿ carta_de_anuencia_e_infraestrutura_da_instituicao.pdf
¿ carta_infraestrutura_da_instituicao.pdf
¿ declaracao_de_vinculo_do_pesquisador.pdf
¿ folha_de_rosto.pdf
¿ folha_de_rosto.pdf
¿ Orcamento_jan21_revised.xlsx
¿ PB_COMPROVANTE_RECEPCAO_2481019.pdf
¿ PB_INFORMAÇÕES_BÁSICAS_DO_PROJETO_2481019.pdf
¿ PB_PARECER_CONSUBSTANCIADO_CEP_7335614.pdf
¿ PROJETO_OBSERVACIONAL_NAPE_INOE_FASE2.doc
¿ PROJETO_OBSERVACIONAL_NAPE_INOE_FASE2.doc
¿ PROJETO_OBSERVACIONAL_NAPE_INOE_FASE2_rev_Jan16.doc
¿ PROJETO_OBSERVACIONAL_NAPE_INOE_FASE2_rev_Jan16.doc
¿ termo_de_confidencialidade.pdf

Recomendações:

Não há.

Conclusões ou Pendências e Lista de Inadequações:

Não se observaram óbices éticos à realização da pesquisa nesta versão.

Endereço: Rua Diniz Cordeiro, 30 - 2º Andar
Bairro: Botafogo **CEP:** 22.281-100
UF: RJ **Município:** RIO DE JANEIRO
Telefone: (21)3883-6013 **Fax:** (21)3883-6013 **E-mail:** cep.idor@idor.org

Considerações Finais a critério do CEP:

Este parecer foi elaborado baseado nos documentos abaixo relacionados:

Tipo Documento	Arquivo	Postagem	Autor	Situação
Informações Básicas do Projeto	PB_INFORMAÇÕES_BÁSICAS_DO_PROJETO_2481019.pdf	22/01/2025 13:40:35		Aceito
Projeto Detalhado / Brochura Investigador	PROJETO_OBSERVACIONAL_NAPE_INOE_FASE2_rev_Jan16.doc	22/01/2025 13:40:14	GIOVANNA NOVAES BORTOLINI	Aceito
Orçamento	Orcamento_jan21_revised.xlsx	22/01/2025 13:02:12	GIOVANNA NOVAES BORTOLINI	Aceito
Declaração do Patrocinador	16_Declaracao_NOEs_Ron_2025_vf.pdf	22/01/2025 13:01:28	GIOVANNA NOVAES BORTOLINI	Aceito
Outros	15_carta_resposta_jan22.docx	22/01/2025 12:59:45	GIOVANNA NOVAES BORTOLINI	Aceito
Parecer Anterior	PB_PARECER_CONSUBSTANCIADO_CEP_7335614.pdf	22/01/2025 12:57:23	GIOVANNA NOVAES BORTOLINI	Aceito
Declaração do Patrocinador	14_62651_Instituto_DOr_Countersigned_Agreement.pdf	22/01/2025 12:52:54	GIOVANNA NOVAES BORTOLINI	Aceito
Outros	07_Termo_Utilizacao_Dados.pdf	22/01/2025 12:48:35	GIOVANNA NOVAES BORTOLINI	Aceito
Outros	06_Termo_Cobertura_Custos.pdf	22/01/2025 12:47:33	GIOVANNA NOVAES BORTOLINI	Aceito
TCLE / Termos de Assentimento / Justificativa de Ausência	10b_INOE_TCLE_Online_rev.docx	22/01/2025 12:44:14	GIOVANNA NOVAES BORTOLINI	Aceito
Outros	Anexo_A_e_B_v2.docx	06/01/2025 17:10:48	GIOVANNA NOVAES BORTOLINI	Aceito
Cronograma	12_Cronograma_Detalhado.docx	06/01/2025 16:06:11	GIOVANNA NOVAES BORTOLINI	Aceito
Declaração de Instituição e Infraestrutura	09_Declaracao_Infraestrutura_RJ.pdf	06/01/2025 16:05:54	GIOVANNA NOVAES BORTOLINI	Aceito
Declaração de	08_Declaracao_Infraestrutura.pdf	06/01/2025	GIOVANNA	Aceito

Instituição e Infraestrutura	08_Declaracao_Infraestrutura.pdf	16:05:46	NOVAES BORTOLINI	Aceito
Outros	05_Delineamento_Amostras.docx	06/01/2025 16:04:32	GIOVANNA NOVAES BORTOLINI	Aceito
Outros	04_Compromisso_Resolucoes.docx	06/01/2025 16:02:52	GIOVANNA NOVAES BORTOLINI	Aceito
Outros	03_Declaracao_confidencialidade_privacidade.pdf	06/01/2025 16:01:00	GIOVANNA NOVAES BORTOLINI	Aceito
Declaração de Pesquisadores	02_Declaracao_Vinculo_Pesquisador.pdf	06/01/2025 16:00:31	GIOVANNA NOVAES BORTOLINI	Aceito
Declaração de Instituição e Infraestrutura	01_Carta_Anuencia_Infra.pdf	06/01/2025 15:59:50	GIOVANNA NOVAES BORTOLINI	Aceito
Folha de Rosto	folha_de_rosto.pdf	06/01/2025 15:02:15	GIOVANNA NOVAES BORTOLINI	Aceito

Situação do Parecer:
Aprovado

Necessita Apreciação da CONEP:
Não

RIO DE JANEIRO, 04 de Fevereiro de 2025

Assinado por:
DENILSON CAMPOS DE ALBUQUERQUE
(Coordenador(a))

Endereço: Rua Diniz Cordeiro, 30 - 2º Andar
Bairro: Botafogo **CEP:** 22.281-100
UF: RJ **Município:** RIO DE JANEIRO
Telefone: (21)3883-6013 **Fax:** (21)3883-6013 **E-mail:** cep.idor@idor.org
