# Supplementary material for: Making sense of the extraordinary – An investigation of the content and prevalence of appraisals of nonordinary experiences
Source: PLoS One. 2026 Aug 5;21(8):e0353126. doi: 10.1371/journal.pone.0353126 (PMC13440842; doi:10.1371/journal.pone.0353126)
Supplement: Supplementary file 6 [file pone.0353126.s005.zip › CEP-material/PB_PARECER_CONSUBSTANCIADO_CEP_7358228-aprovado_translated.pdf]

Machine Translated by Google

SUBSTANTIATE OPINION OF THE CEP

RESEARCH PROJECT DATA

Research Title: Psychological profile of Non-Ordinary Experiences (NOE) and its associations with individual differences in the Brazilian context.

Researcher: Ronald Fischer

Thematic Area:

Version: 2

CAAE: 85708425.3.0000.5249

Proposing Institution: D'OR INSTITUTE OF RESEARCH AND EDUCATION

Main Sponsor: D'OR INSTITUTE OF RESEARCH AND EDUCATION
D'OR INSTITUTE OF RESEARCH

OPINION DATA

Opinion Number: 7,358,228

Project Presentation: The

information contained in the sections "Project Presentation", "Research Objective", "Risk and Benefit Assessment" was extracted from the document PB_INFORMAÇÕES_BÁSICAS_DO_PROJETO_2481019.

INTRODUCTION:

Anomalous experiences, such as the sensation of being touched or hearing a voice when no one is around, feeling that one's thoughts seem externally controlled, or even losing the perception of time and space, can be interpreted as religious, anomalous or pathological, depending on the cultural origin and individual characteristics of those who experience them (Acunzo et al., 2020; Cardeña et al., 2014).
Furthermore, people may interpret these experiences as very positive and highly meaningful, or as disturbing, intriguing, or meaningless. In search of understanding and based on such interpretations, it is common to consult religious or medical experts. The type of expert consulted can result in the spiritualization or medicalization of the experience (Moreira-Almeida et al., 2008). Interpretation also appears to influence the relationship between experiences and well-being. The literature analyzing this relationship presents contradictory results. One possible explanation

Machine Translated by Google

These results are due precisely to the fact that, in general, research does not separate the experience itself from its attribution of meaning or valence. This can generate biases based on the interpretation implicit in the scale used for measurement (Irwin et al., 2013; Lange et al., 2019; Taves, 2020) or the cultural context that values or stigmatizes that experience. In this sense, the Inventory of Non-Ordinary Experiences (INOE) was developed by Taves and colleagues (Taves, 2020; Taves & Barlev, 2022) to fill the gaps and biases observed in pre-existing instruments. The INOE focuses on subjectively recognizable aspects of experiences rather than predefined constructs, seeking to separate the phenomenological characteristics of experiences from culturally infused interpretations. Thus, the inventory broadens the range of experiences measured by including some that may be relatively common but may still be salient for people in some cultures and contexts. This more comprehensive list, encompassing potentially positive and negative experiences (from experiencing strong positive emotions to near-death experiences), broadens the scope of previous work, which focused solely on clinically relevant or paranormal experiences (American Psychiatric Association, 2013; Kelly, 2014; World Health Organization, 1993). Using the INOE in data collection will offer greater flexibility to the project, since the instrument does not aim to measure a single construct (Taves et al., 2019), allowing the option to apply only part of its items—that is, only the anomalous experiences most relevant to the study. The same applies to additional items related to the interpretation attributed to the experiences—context, spirituality, causative agent, perception of control, impact on life, etc. It is important to investigate the frequency and distribution of NOEs in representative samples. How common are the different types of NOEs, given widespread estimates of less than 10% to around 80% in studies using researcher-guided experience scales? Describing the frequency and distribution of NOEs in a population is of great interest to various scholars, primarily physicians and mental health professionals, but also anthropologists and psychologists interested in the functioning of the mind (Luhrmann et al., 2015a). A second question is the possibility of exploring the clustering of these experiences and their assessments. As mentioned above, each item is considered a unique phenomenon, similar to a latent variable in psychology. At the same time, it is possible that some experiences co-occur in the general population, either through possible common neurobiological pathways (Cohen & Barrett, 2008; Cristofori et al., 2016; Kapogiannis et al., 2009) or by virtue of cultural socialization (e.g., Luhrmann et al., 2015b; Maraldi & Krippner, 2019). By

Machine Translated by Google

By exploring the clustering of these experiences, it is possible to gain new insights into the structure of the human mind and non-ordinary experiences, which will be informative for understanding psychopathology and psychological well-being. To understand the psychological mechanisms underlying non-ordinary experiences, many authors have worked to identify individual traits and characteristics capable of better predicting them. Irwin (2009) notes that some meaning-making styles, such as paranormal attributions and beliefs, are more commonly observed in people who report non-ordinary experiences. In addition, some aspects of personality and cognition seem to correlate simultaneously with the experience of non-ordinary experiences and with paranormal-oriented attribution styles, among which the following stand out: schizotypy, magical ideation, sensory anomalies (Claridge & Beech, 1995; Irwin, 2009; Wolfradt, 1999), emotional reasoning, suspension of reality testing (Irwin, 2009), specific personality traits (e.g., extraversion: Rattet, 2001; openness: McCrae, 1994), absorption (Tellegen & Atkinson, 1974), fantasy proneness (Woody & Barnier, 2008), and transliminality (Sherwood & Milner, 2005; Thalbourne & Houran, 2000). Beyond the particularities of personality, while inseparable from them, life history and the external events to which the individual is exposed also correlate with reports of non-ordinary experiences (Cardeña, Lynn & Krippner, 2017; Parra, 2019). The association between traumatic events occurring in childhood and adolescence and indicators of mental distress in adulthood is well-established in the psychiatric literature.

Exposure to acute or chronic trauma is known to be linked to depression (Mandelli, Petrelli & Serretti, 2015), bipolar disorder (Aas et al., 2016), personality disorders (Cummings, Berkowitz & Scribano, 2012), post-traumatic stress disorder (Yehuda, Halligan & Grossman, 2001) and impairments in cognitive and socio-emotional skills (Enoch, 2011). The association between childhood trauma and the development of dissociative states and reports of non-ordinary experiences has been widely explored in the literature (Lawrence et al., 1995; McLewin & Muller, 2005; Parra, 2019; Rabeyron & Watt, 2010; Toutountzidis et al., 2022), and some authors suggest that these processes may have an adaptive function in response to adverse experiences (Bailey & Brand, 2017; Marks, 2020) and act to mitigate psychopathological symptoms (Corso et al., 2023). It is speculated that both beliefs in supernatural phenomena and non-ordinary experiences provide a means of symbolization and meaning-making in the face of adversity, so that some interpretation of the traumatic event becomes possible (Lasikiewicz, 2016; Park, 2010; Rabeyron & Loose, 2015). However, the potential

Machine Translated by Google

The adaptive function of non-ordinary experiences is unclear, and it is understood that they can also present a problematic dimension for mental health, contributing to the emergence of negative emotions, stigma, and social isolation (Rabeyron, 2022), or they can function as a protective factor (Lasikiewicz, 2016; Park, 2010; Rabeyron & Loose, 2015). As seen previously, it is possible that both the phenomenological quality of anomalous experiences and the way they are subjectively and culturally interpreted influence the strength and direction of their effect on mental health (Hartle et al., 2023; Maraldi et al., 2023). Studies describe that interpretations of causality attributed to external agents, as well as a greater attempt to control the event and feelings of altered cognitive functioning, are associated with negative emotional impacts, while experiences interpreted from a spiritual perspective, with a greater perception of control and a sense of acceptability and social support, tend to produce less suffering (Brett et al., 2014). Therefore, using instruments validated for the Brazilian context, this research project seeks to elucidate the complex association between non-ordinary experiences, personality and cognitive characteristics, a history of adverse life events, and mental health indicators. A particularly important aspect will be the investigation focused on both the phenomenological dimension of the experience and its subjective interpretation—the way individuals attribute meaning and value to anomalous phenomena—and how this interpretation relates to psychological well-being outcomes. This will advance the understanding of psychological mechanisms related to non-ordinary experiences, the distinction between the non-ordinary and the psychopathological, and the reduction of associated stigma.

HYPOTHESIS:

Study Hypotheses:

Hypothesis 1: The prevalence and distribution of non-ordinary experiences in the Brazilian population will be heterogeneous according to sociodemographic factors, as well as according to religious affiliation.

Hypothesis 2: Religious affiliations that encourage non-ordinary experiences will show a higher frequency of non-ordinary experiences.

Hypothesis 3: Individuals with a higher fantasy and openness index will report more non-fantasy experiences.
ordinary.

Hypothesis 4: Individuals with a higher index of extraversion will report more non-extroversion experiences.

Machine Translated by Google

ordinary.

Hypothesis 5: Individuals with a higher need for certainty will report fewer non-certainty experiences.
ordinary.

Hypothesis 6: Younger individuals will report more non-ordinary experiences.

Hypothesis 7 Individuals in religious contexts will report more non-ordinary experiences.

Hypothesis 8: Individuals with a history of traumatic experiences will report more non-traumatic experiences.
ordinary.

Hypothesis 9: The attribution of supernatural meaning to non-ordinary experience will correlate with personality traits, values, beliefs, and ways of thinking.

PROPOSED METHODOLOGY:

Data collection: For recruitment, we will use the snowball method and online panel companies.
that operate in accordance with Brazilian law (e.g., www.netquest.com; https://www.opinionbox.com/painel/; https://www.qualtrics.com/pt-br/) and contact information for the research team. Participant recruitment and participation will comply with the guidelines of the General Data Protection Law (LGPD) and international data protection guidelines. Data collection will be conducted with a representative sample of the Brazilian population, consisting of participants from online panels. The target population of the study corresponds to the adult Brazilian population. The sample will be distributed proportionally by age, gender, region, and socioeconomic status. Data analysis: Initially, we will use descriptive statistical analyses of frequencies, means, and variability using the psych package in the R language. Subsequently, we will use Exploratory Graph Analysis (EGA) (28) to identify possible clusters of experiences with other psychological variables. Linear regression analyses will also be performed using the same software, in the R language.

Instruments: To construct a comprehensive psychological profile of the research participants, several psychometric tests widely recognized in the scientific literature and validated for the Brazilian context will be used. These instruments assess different psychological dimensions and individual characteristics, such as personality, cognition, emotions, well-being, and life experiences. For example, personality tests include the Big Five Personality Inventory (John, 1991), which measures the five major personality traits, and the IPO Personality Inventory (Lenzenweger, 2001), which explores aspects of organizational behavior.

Machine Translated by Google

personality. To assess beliefs and attitudes, the Rokeach Dogmatism Scale (Rokeach, 1960) and the Sense of Coherence Scale (Antonovsky, 1993) will be applied. In the field of life experiences, instruments such as the Adverse Childhood Experiences Questionnaire (ACE) (Felitti et al., 1998) and the Childhood Trauma Questionnaire (CTQ) (Bernstein et al., 2003) analyze the impact of adverse childhood events. Other tests, such as the Sleep Condition Indicator (Espie et al., 2014), investigate sleep quality, while scales such as the Brief COPE (Carver, 1997) assess coping strategies in challenging situations. Additionally, the Visual Image Vividness Questionnaire (VVIQ) (Marks, 1973) measures the vividness of visual mental imagery, and the Phenomenological Control Scale (Lush, 2021) examines the ability to control perceptual experiences. The full instrument list is available in Appendix A and will form the item bank to be presented to participants using the Massive Missing Completely at Random (MMCAR) method (Reveille, 2024), which allows for the measurement of a large number of constructs in representative samples to cover a broad behavioral scope. The method is similar to an array of radio telescopes that individually are limited to a single, noisy coverage, but an array of thousands of radio telescopes directed in all geographic directions results in a clear image of the entire sky.

We randomly present items translated and validated into Portuguese. The estimated time for volunteers to participate in the survey is 15 minutes. Sample items can be found in Appendix B.

INCLUSION CRITERIA:

Brazilians over 18 years old, literate.

EXCLUSION CRITERIA:

Individuals who are not fully able to understand and/or respond to the instruments used to collect data
data.

Failure to complete the TCLE.

Incorrect or incomplete completion of one or more study instruments.

DATA ANALYSIS METHODOLOGY:

Initially, we will use descriptive statistical analyses of frequencies, means, and variability using the psych package in the R language. Subsequently, we will use Exploratory Graph Analysis (EGA) (28) to identify possible clusters of experiences with other psychological variables. Regression analyses will also be performed.

Machine Translated by Google

linear analysis using the same software, using the R language. The data will be stored on servers maintained by the institute, with a dedicated IT team for maintenance and aligned with the main digital security and LGPD guidelines. The data will be securely stored on an Amazon Web Services, Inc. (AWS) server, anonymized and protected by encryption. Several security requirements are implemented to ensure data confidentiality and security, including: MFA (multiple-factor authentication), CloudTrail tracking of all accounts, private buckets, a firewall enabled for all accesses, and Cognito (an identity pool for AWS credentials).

PRIMARY OUTCOME:

We expect a complex panorama of associations between non-ordinary experiences and psychological factors.

SECONDARY OUTCOME:

On a positive note, we expect that people with higher fantasy and openness scores will report more unusual experiences. We also expect that social support will mitigate any negative effects of these unusual experiences on mental health.

Sample Size in Brazil: 20,000

Will secondary data sources (medical records, demographic data, etc.) be used?
No

Report the number of individuals personally approached, recruited, or who will undergo some type of intervention at this research center: 20,000

Research Objective:

PRIMARY OBJECTIVE:

Explore the prevalence, clustering patterns, and psychological correlates of non-ordinary experiences in order to identify potential predictors that relate to a greater likelihood of
experience them.

SECONDARY OBJECTIVE:

Understand the prevalence of non-ordinary experiences using representative samples that

Machine Translated by Google

make it possible to report the distribution of non-ordinary experiences in Brazil among the general population and across different religious affiliations.

Investigate the existence of psychological profiles that increase the likelihood of reporting non-life-threatening experiences. ordinary.

Explore the clustering of non-ordinary experiences and their psychological correlates.

To test whether non-ordinary experiences act as protective factors against the effects of trauma on mental health in adulthood.

Risk and Benefit Assessment:

RISKS:

Studies on the ethical aspects of trauma-focused research suggest that the use of self-report instruments or interviews is associated with feelings of momentary discomfort in a small portion of participants (Ferrier-Auerbach et al., 2009; Jaffe et al., 2015; Yeater et al., 2012).

On the other hand, the discomfort reported by participants is mild and is outweighed by the perceived benefit of participating in the research, generally considered a positive experience (Jaffe et al., 2015, Newman & Kaloupek, 2004). At the time of data collection, conducted through the FormR platform, participants should be warned in advance about the content of the questionnaire and the possibility of it causing emotional discomfort. At this point, the possibility of the participant not answering any questions and stopping participation in the research at any time will be emphasized. Other aspects considered at the beginning of the questionnaire will be: a detailed explanation of the study's purposes and its possible direct and indirect benefits, an indication of the estimated time to complete the questionnaire, and

the use of empathetic and non-judgmental language. At the end of the questionnaire, information will be included about the impacts of trauma on mental health and the possibility of seeking professional help when needed, along with information about mental health services in the RAPS and the opportunity for participants to provide feedback to the researchers.

BENEFITS:

Direct benefits: Completing the questionnaires provides participants with the opportunity to reflect on how their experiences and personal characteristics may have impacted their current well-being, helping to identify support needs. The information provided at the end of the questionnaires may also contribute to

Machine Translated by Google

by destigmatizing experiences and traumas, promoting a more compassionate and non-judgmental perception of the topic. Indirect benefits: The study results will provide data on the prevalence of non-ordinary experiences in Brazil, their relationship with mental health variables and individual characteristics, contributing to psychology in theoretical and clinical settings, as well as to public health.

Comments and Considerations on the Research:

The previous ethical assessment highlighted the following pending issues, which were addressed by the researcher:

1. The project must detail all procedures that will be performed during the research. For example, it must be clarified which procedures will be performed remotely and in person, and where these steps will be executed (item 3.4 of Operational Standard No. 001/2013).

RESEARCHER'S RESPONSE: The study will be conducted entirely online at this initial stage. In-person data collection will be considered in a future phase, after analyzing the online results and identifying and clarifying key points raised during this analysis. This strategy allows for optimized planning and ensures greater efficiency in conducting the study. The project has been adjusted to include an exclusively online version at this stage. If in-person data collection is conducted in the future, it will be subject to a new authorization process by the CEP.

REPORTER'S OPINION: Issue resolved.

2. In the Informed Consent Form (TCLE), there are unfilled fields highlighted in yellow, such as the name of the institution, project title, and release date. Furthermore, this form must be written in clear language that is accessible to research participants (item IV.1b of CNS Resolution No. 466/2012). Therefore, the "Study Objectives" and "Justification" sections of the Informed Consent Form (TCLE) must be revised to comply with the aforementioned regulations.

RESEARCHER'S RESPONSE: We filled in the open fields, including the name of the

Machine Translated by Google

institution, project title, and release date. Furthermore, we revised the objectives and justification sections, aligning them with the guidelines presented in item IV.1b of CNS Resolution No. 466/2012.

REPORTER'S OPINION: Issue resolved.

3. Furthermore, in the in-person version of the TCLE, the guarantee of reimbursement of expenses incurred by research participants and arising therefrom must be explicit (item IV.3g of CNS Ordinance No. 466/2012).

RESEARCHER'S RESPONSE: As mentioned in item 1 above, we clarify that data collection will be carried out online at this stage of the research.

REPORTER'S OPINION: The in-person version of the TCLE was removed from the Plataforma Brasil, leaving only the remote version. Issue resolved.

4. A grant letter or similar document containing a guarantee of project financing from the sponsor must be submitted. Always submit a detailed budget.

RESEARCHER'S RESPONSE: We have included a letter from the Pioneering Science Initiative/IDOR, which details the project's scientific background and the support offered for the ongoing research. We have also attached the contract signed between the D'Or Institute for Research and Education and the John Templeton Foundation, which outlines the budget available for this research.

REPORTER'S OPINION: A letter signed by the scientific director of Ciência Pioneira/IDOR was included, explaining that this initiative will be responsible for funding a postdoctoral fellowship. The contract signed between IDOR and the John Templeton Foundation will cover the remaining project expenses, as explained by the researcher. The matter has been resolved.

5. There are documents submitted to Plataforma Brasil that do not contain the signature of the researcher or the institutional manager, namely: 6_Termo_Cobertura_Custos.doc, 07_Termo_Utilizacao_Dados.doc.
Signed versions of these documents must be sent via the Platform.

Machine Translated by Google

RESEARCHER'S RESPONSE: Our team apologizes for initially submitting a document without the responsible person's signature. We are now sending all documents received by NAPE duly signed by the responsible person, as required by Plataforma Brasil.

REPORTER'S OPINION: Issue resolved.

Considerations on the Mandatory Presentation Terms: The following documents were analyzed in this ethical assessment:
¿01_Carta_Anuencia_Infra.pdf
¿01_Carta_Anuencia_Infra.pdf
¿02_Declaracao_Vinculo_Pesquisador.pdf
¿02_Declaracao_Vinculo_Pesquisador.pdf
¿03_Declaracao_confidencialidade_privacidade.pdf
¿03_Declaracao_confidencialidade_privacidade.pdf
¿04_Compromisso_Resolucoes.docx
¿04_Compromisso_Resolucoes.docx
¿05_Delineamento_Amostras.docx
¿05_Delineamento_Amostras.docx
¿06_Termo_Cobertura_Custos.doc
¿06_Termo_Cobertura_Custos.doc
¿06_Term_Cobertura_Custos.pdf
¿07_Term_Utilizacao_Dados.doc
¿07_Term_Utilizacao_Dados.doc
¿07_Term_Utilizacao_Dados.pdf
¿08_Declaracao_Infraestrutura.pdf
¿08_Declaracao_Infraestrutura.pdf
¿09_Declaracao_Infraestrutura_RJ.pdf
¿09_Declaracao_Infraestrutura_RJ.pdf
¿10a_INOE_TCLE_Presencial_nov26_2024.docx
¿10a_INOE_TCLE_Presencial_nov26_2024.docx
¿10a_INOE_TCLE_Presencial_nov26_2024.docx

Machine Translated by Google

¿10b_INOE_TCLE_Online.docx

¿10b_INOE_TCLE_Online.docx

¿10b_INOE_TCLE_Online.docx

¿10b_INOE_TCLE_Online_rev.docx

¿12__Detalhado.docx

¿12__Detalhado.docx

¿13_PerfilpsicologicoINOE_Orcamento.docx

¿13_PerfilpsicologicoINOE_Orcamento.docx

¿14_62651_Instituto_DOr_Countersigned_Agreement.pdf

¿15_carta_resposta_jan22.docx

¿16_Declaracao_NOEs_Ron_2025_vf.pdf

¿Anexo_A_e_B_v2.docx

Annex_A_and_B_v2.docx

Letter_of_Consent_and_Infrastructure_of_the_Institution.pdf

Letter_of_Infrastructure_of_the_Institution.pdf

Researcher_Statement_of_Linkage.pdf Cover_sheet.pdf

Cover_sheet.pdf

Budget_Jan21_Revised.xlsx

PB_COMPROVANTE_RECEPCAO_2481019.pdf

PB_INFORMAÇÕES_BÁSICAS_DO_PROJETO_2481019.pdf

PB_PARECER_CONSUBSTANCIADO_CEP_7335614.pdf

PROJETO_OBSERVACIONAL_NAPE_INOE_FASE2.doc

¿PROJETO_OBSERVACIONAL_NAPE_INOE_FASE2.doc

¿PROJETO_OBSERVACIONAL_NAPE_INOE_FASE2_rev_Jan16.doc

¿PROJETO_OBSERVACIONAL_NAPE_INOE_FASE2_rev_Jan16.doc

¿termo_de_confidencialidade.pdf

Recommendations:

There is none.

Conclusions or Pending Issues and List of Inadequacies: No ethical obstacles were observed to carrying out the research in this version.

Machine Translated by Google

Final Considerations at the discretion of the CEP:

This opinion was prepared based on the documents listed below:

Type Document File PB_INFORMAÇÕES_BÁSICAS_DO_P Basic		Post	Author	Situation
PROJETO_OBSERVACIONAL_NAPE_I Brochure	Project Information ROJETO_2481019.pdf Detailed Project /	01/22/2025 13:40:35		Accepted
NOE_FASE2_rev_Jan16.doc Researcher Budget		01/22/2025 13:40:14	GIOVANNA NOVAES BORTOLINI	Accepted
	Budget_Jan21_revised.xlsx	01/22/2025 1:02:12 PM	GIOVANNA NOVAES BORTOLINI	Accepted
Declaration of Sponsor	16_Declaracao_NOEs_Ron_2025_vf.pdf 01/22/2025 13:01:28		GIOVANNA NOVAES BORTOLINI	Accepted
Others	15_letter_response_jan22.docx	01/22/2025 12:59:45 PM	GIOVANNA NOVAES BORTOLINI	Accepted
Previous Opinion PB_PARECER_CONSUBSTANCIADO_ CEP_7335614.pdf		01/22/2025 12:57:23 PM	GIOVANNA NOVAES BORTOLINI	Accepted
Declaration of Sponsor	14_62651_Instituto_DOr_Countersigned _Agreement.pdf	01/22/2025 12:52:54	GIOVANNA NOVAES BORTOLINI	Accepted
Others	07_Terms_of_Data_Use.pdf	01/22/2025 12:48:35	GIOVANNA NOVAES BORTOLINI	Accepted
Others	06_Term_Coverage_Costs.pdf	01/22/2025 12:47:33 PM	GIOVANNA NOVAES BORTOLINI	Accepted
TCLE / Terms of Assent / Justification of Absence	10b_INOE_TCLE_Online_rev.docx	01/22/2025 12:44:14 PM	GIOVANNA NOVAES BORTOLINI	Accepted
Others	Annex_A_and_B_v2.docx	06/01/2025 17:10:48	GIOVANNA NOVAES BORTOLINI	Accepted
Timeline	12_Detailed_Schedule.docx	06/01/2025 16:06:11	GIOVANNA NOVAES BORTOLINI	Accepted
Declaration of Institution and Infrastructure	09_Declaracao_Infraestrutura_RJ.pdf	06/01/2025 16:05:54	GIOVANNA NOVAES BORTOLINI	Accepted
Declaration of	08_Declaracao_Infraestrutura.pdf	06/01/2025 GIOVANNA		Accepted

Machine Translated by Google

Institution and Infrastructure	08_Declaracao_Infraestrutura.pdf	16:05:46	NOVAES BORTOLINI	Accepted
Others	05_Sample_Design.docx	06/01/2025 16:04:32	GIOVANNA NOVAES BORTOLINI	Accepted
Others	04_Commitment_Resolutions.docx	06/01/2025 16:02:52	GIOVANNA NOVAES BORTOLINI	Accepted
Others	03_Declaration_confidentiality_privacy.pdf	06/01/2025 16:01:00	GIOVANNA NOVAES BORTOLINI	Accepted
Declaration of Researchers	02_Declaration_Link_Researcher.pd f	06/01/2025 16:00:31	GIOVANNA NOVAES BORTOLINI	Accepted
Declaration of Institution and Infrastructure	01_Carta_Anuencia_Infra.pdf	06/01/2025 15:59:50	GIOVANNA NOVAES BORTOLINI	Accepted
Title Page	face_sheet.pdf	06/01/2025 15:02:15	GIOVANNA NOVAES BORTOLINI	Accepted

Opinion Status:

Approved

Needs CONEP's Appreciation:

No

RIO DE JANEIRO, February 4, 2025

Signed by:
DENILSON CAMPOS DE ALBUQUERQUE
(Coordinator)

Address: Diniz Cordeiro Street, 30 - 2nd Floor
Neighborhood: Botafogo **ZIP Code:** 22.281-100
State: RJ **Municipality:** RIO DE JANEIRO
Telephone: (21)3883-6013 **Fax:** (21)3883-6013 **Email:** cep.idor@idor.org
