# Supplementary material for: Making sense of the extraordinary – An investigation of the content and prevalence of appraisals of nonordinary experiences
Source: PLoS One. 2026 Aug 5;21(8):e0353126. doi: 10.1371/journal.pone.0353126 (PMC13440842; doi:10.1371/journal.pone.0353126)
Supplement: Peer review history

# Peer review: Decision Letter 0

**DOI:** 10.1371/journal.pone.0353126.r001

**Authors:** David Smailes

2 Dec 2025

PONE-D-25-47007

Making sense of the extraordinary: An exploration of the content and prevalence of appraisals of nonordinary experiences

PLOS ONE

Dear Dr. Fischer,

Thank you for submitting your manuscript to PLOS ONE. After careful consideration, we feel that it has a great deal of merit but some revisions are required. Therefore, we invite you to submit a revised version of the manuscript that addresses the points raised during the review process.

======================================

The reviewers have provided a really useful set of comments that – when addressed – should improve the manuscript. In addition to the comments from the reviewers, please consider the comments from me below.

Sub01AE1 = I think it would be very helpful for readers if a simple hyperlink (such as https://doi.org/10.1371/journal.pone.0287780.s001) was inserted in the MS, that ‘takes’ readers to a copy of the original INOE. This would make it easier for the reader to get a good understanding of the range of experiences classed as an ‘NOE’ (although that may just reflect my lack of familiarity with this concept).

Sub01AE2 = On line 355, the study’s pre-registration is mentioned and a citation is provided for that pre-registration. However, I do not think that the document cited is a pre-registration (although it may contain info about the pre-registration). I think this - https://osf.io/ze6cb/overview – link needs to be provided at line 355 instead? Apologies if I am mistaken.

Sub01AE3 = On lines 752-753, two papers are cited re: predictive coding and source-monitoring theories of hallucinations. I am not sure, however that these are particularly effective publications to cite here. Other papers – such as https://academic.oup.com/schizophreniabulletin/article/38/4/683/1870142 or https://www.cell.com/trends/cognitive-sciences/fulltext/S1364-6613(18)30282-1 or https://www.annualreviews.org/content/journals/10.1146/annurev-clinpsy-032816-045145 - may be better to cite (although it does depend on the precise point you ar emaking; especially if the aim is to cite a paper that supports the claim that being alone/socially isolated may affect predictive processing/source monitoring in a way that increases the likelihood of a hallucinatory experience).

Sub01AE4 = Can you please make the OSF project page assocaited with the manuscript 'public' please? PLOS ONE operates single-blind peer review (reviewers can remain anonymous; authors do not), and so making the OSF project page will not 'unblind' the reviewers. The study data/materials were made available to me after you submitted the manuscript, but i think this was by adding me as a contributor to the project (I have now removed mysefl from the project, so that it is clear that I have no conflict of interest re: me acting as editor for this manuscript), and I believe the necessary data/scripts are available on the OSF. It would be great if the reviewers could examine this, too, when they review your resubmission.

I should note, however, that both reviewers, as well as me, have a background in hallucinations/psychosis research. And so some of our comments/assumptions may well be shaped by that background

=======================================

Please submit your revised manuscript by Jan 16 2026 11:59PM. If you will need more time than this to complete your revisions, please reply to this message or contact the journal office at plosone@plos.org. When you're ready to submit your revision, log on to https://www.editorialmanager.com/pone/ and select the 'Submissions Needing Revision' folder to locate your manuscript file.

Please include the following items when submitting your revised manuscript:

- A rebuttal letter that responds to each point raised by the academic editor and reviewer(s). You should upload this letter as a separate file labeled 'Response to Reviewers'.
- A marked-up copy of your manuscript that highlights changes made to the original version. You should upload this as a separate file labeled 'Revised Manuscript with Track Changes'.
- An unmarked version of your revised paper without tracked changes. You should upload this as a separate file labeled 'Manuscript'.

-->If you would like to make changes to your financial disclosure, please include your updated statement in your cover letter. Guidelines for resubmitting your figure files are available below the reviewer comments at the end of this letter.

If applicable, we recommend that you deposit your laboratory protocols in protocols.io to enhance the reproducibility of your results. Protocols.io assigns your protocol its own identifier (DOI) so that it can be cited independently in the future. For instructions see: https://journals.plos.org/plosone/s/submission-guidelines#loc-laboratory-protocols. Additionally, PLOS ONE offers an option for publishing peer-reviewed Lab Protocol articles, which describe protocols hosted on protocols.io. Read more information on sharing protocols at https://plos.org/protocols?utm_medium=editorial-email&utm_source=authorletters&utm_campaign=protocols.

We look forward to receiving your revised manuscript.

Kind regards,

David Smailes, Ph.D.

Academic Editor

PLOS ONE

Journal Requirements:

When submitting your revision, we need you to address these additional requirements.

1. Please ensure that your manuscript meets PLOS ONE's style requirements, including those for file naming. The PLOS ONE style templates can be found at

https://journals.plos.org/plosone/s/file?id=wjVg/PLOSOne_formatting_sample_main_body.pdf and

https://journals.plos.org/plosone/s/file?id=ba62/PLOSOne_formatting_sample_title_authors_affiliations.pdf.

2. Thank you for stating in your Funding Statement:

“The work was supported by a grant from the John Templeton Foundation (Grant ID: 62651), the Pioneer Science Initiative of the D’Or Institute for Research and Education, Rio de Janeiro, Brazil and a grant by the Conselho Nacional de Desenvolvimento Científico e Tecnologico (Process: 309085/2023-3) to the last author.”

Please provide an amended statement that declares *all* the funding or sources of support (whether external or internal to your organization) received during this study, as detailed online in our guide for authors at http://journals.plos.org/plosone/s/submit-now.  Please also include the statement “There was no additional external funding received for this study.” in your updated Funding Statement.

Please include your amended Funding Statement within your cover letter. We will change the online submission form on your behalf.

3. Thank you for stating the following financial disclosure:

“The work was supported by a grant from the John Templeton Foundation (Grant ID: 62651), the Pioneer Science Initiative of the D’Or Institute for Research and Education, Rio de Janeiro, Brazil and a grant by the Conselho Nacional de Desenvolvimento Científico e Tecnologico (Process: 309085/2023-3) to the last author.”

Please state what role the funders took in the study.  If the funders had no role, please state: "The funders had no role in study design, data collection and analysis, decision to publish, or preparation of the manuscript."

If this statement is not correct you must amend it as needed.

Please include this amended Role of Funder statement in your cover letter; we will change the online submission form on your behalf.

4. Please be informed that funding information should not appear in the Acknowledgments section or other areas of your manuscript. We will only publish funding information present in the Funding Statement section of the online submission form. Please remove any funding-related text from the manuscript.

5. Your ethics statement should only appear in the Methods section of your manuscript. If your ethics statement is written in any section besides the Methods, please delete it from any other section.

6. We note that you have referenced (Fischer R, Bortolini G., Bortolini TS,  Maraldi EO, ED, Hartle L, et al. Revisiting the Unusual: ) which has currently not yet been accepted for publication. Please remove this from your References and amend this to state in the body of your manuscript: (Fischer R, Bortolini G., Bortolini TS,  Maraldi EO, ED, Hartle L, et et al. [Unpublished]”) as detailed online in our guide for authors

http://journals.plos.org/plosone/s/submission-guidelines#loc-reference-style.

7. Please include captions for your Supporting Information files at the end of your manuscript, and update any in-text citations to match accordingly. Please see our Supporting Information guidelines for more information: http://journals.plos.org/plosone/s/supporting-information.

8. If the reviewer comments include a recommendation to cite specific previously published works, please review and evaluate these publications to determine whether they are relevant and should be cited. There is no requirement to cite these works unless the editor has indicated otherwise.

9. Please review your reference list to ensure that it is complete and correct. If you have cited papers that have been retracted, please include the rationale for doing so in the manuscript text, or remove these references and replace them with relevant current references. Any changes to the reference list should be mentioned in the rebuttal letter that accompanies your revised manuscript. If you need to cite a retracted article, indicate the article’s retracted status in the References list and also include a citation and full reference for the retraction notice.

[Note: HTML markup is below. Please do not edit.]

Reviewers' comments:

Reviewer's Responses to Questions

**Comments to the Author**

1. Is the manuscript technically sound, and do the data support the conclusions?

The manuscript must describe a technically sound piece of scientific research with data that supports the conclusions. Experiments must have been conducted rigorously, with appropriate controls, replication, and sample sizes. The conclusions must be drawn appropriately based on the data presented.

Reviewer #1: Yes

Reviewer #2: Yes

**********

2. Has the statistical analysis been performed appropriately and rigorously?

Reviewer #1: Yes

Reviewer #2: Yes

**********

3. Have the authors made all data underlying the findings in their manuscript fully available?

The PLOS Data policy requires authors to make all data underlying the findings described in their manuscript fully available without restriction, with rare exception (please refer to the Data Availability Statement in the manuscript PDF file). The data should be provided as part of the manuscript or its supporting information, or deposited to a public repository. For example, in addition to summary statistics, the data points behind means, medians and variance measures should be available. If there are restrictions on publicly sharing data—e.g. participant privacy or use of data from a third party—those must be specified.

Reviewer #1: Yes

Reviewer #2: Yes

**********

4. Is the manuscript presented in an intelligible fashion and written in standard English?

PLOS ONE does not copyedit accepted manuscripts, so the language in submitted articles must be clear, correct, and unambiguous. Any typographical or grammatical errors should be corrected at revision, so please note any specific errors here.

Reviewer #1: Yes

Reviewer #2: No

**********

5. Review Comments to the Author

Please use the space provided to explain your answers to the questions above. You may also include additional comments for the author, including concerns about dual publication, research ethics, or publication ethics. (Please upload your review as an attachment if it exceeds 20,000 characters)

Reviewer #1: Thank you for this paper. I have some minor revisions.

Study I

• Abstract

A stronger conclusion is needed. the meaning and importance of the paper is not clear.

Introduction

• It is difficult to follow across the Tables what the included appraisal items were. How they were worded? and why certain appraisal were included or excluded. The paper would benefit from a copy of the Appraisal Form either in a new Table, In Table 1 instead of the adaptations column or in supplemental material

o It was unclear whether appraisals of personal/ non personal, benevolent/ benign/ malevolent, omnipotence/ omniscience/ neutrality were included? These are important appraisals with the AANEX, and Beliefs about Voices questionnaires, and are predictive of distress and believability. If they were excluded, a justification should be given. God’s intentions, for instance, maybe appraised as benevolent or malevolent.

o Cognitive theories would suggest that it is when an negative appraisal is matched with safety behaviours that a maintaining process of distress occurs. (for instance https://doi.org/10.1016/j.brat.2012.10.008). A reflection on how benign experiences may become psychopathological would be helpful.

• Methods

o No demographic data was collected due to time restrictions- this is a limitation and should be noted especially in relation to validity and generalisability.

o “Sample size was determined by the stopping criteria described in the section entitled 'Decision criteria for item validation” could this be explained further in the text. N=160 seems small for a multi-item scale and strong justification is needed.

• Results:

• Missing data should be discussed at a sample and item level.

• VS= full wording should be used

o The table location and formatting through out the document is difficult to follow.

Study II

• Results- item level missing data should be report

• What was the missing data strategy?

Table 4 should be presented in the results section

• Discussion

o “This is particularly important when considering that a vast majority of experiences, including negative experiences, in the long run, were associated with positive effects overall. This shows the enormous potential of human resilience and requires more focused follow-up studies.”

o I’m particularly struck by this comment. It fits the wider literature, (i.e., https://doi.org/10.1002/wps.20301) and highlights the importance of protective factors, and natural human resilience. I wonder would this be an appropriate conclusion for the abstract?

Thank you for this paper

Reviewer #2: Overview:

These studies offer valuable contributions to the literature on non-ordinary experiences, addressing the important and thorny issue of attempting to disentangle NOEs and appraisals of these experiences. The studies and analyses themselves are generally well done. However, the paper in terms of its framing, language and overall communication of the studies and their position in the wider literature needs substantial editing.

There are times where the goals and scope of the paper are unclear. There is also a need for a fair bit of editing in terms of language precision. I have included a number of specific language edits in my recommendations below, but please proofread the whole paper for grammar and clarity of language.

Changes needed:

The definition and scope of NOEs as a concept need to be made clear at the outset. At one point you refer to “subjectively salient” experiences (lines 104-106) and towards the end of the paper (705-706) they appear to be officially defined this way. However, this needs to be stated clearly early on.

Furthermore, a discussion of the reasoning behind focusing on experiences which are subjectively salient, as discussed by Taves et al. (2023), is needed. You’ve described quite a wide array of experiences ranging from voice-hearing to love and despair as non-ordinary experiences (e.g. lines 96-99). The paper needs to directly address the fact that this wide range of experiences are being discussed as part of the same category.

The paper should also provide a short, clear definition of appraisal early on. As reference, Taves et al.’s original paper includes the following clear definition: “Appraisal, as used here, refers to the outcome of a multi-level process that humans and other animals rely on when determining what is happening, that is, to interpret situations and events”

89 There is a conjunction missing before the word “strong”

97-99 “On the other hand, phenomenologically comparable experiences are being used in psychiatric manuals as symptoms of dissociation or hallucinations” This is unclear because hallucination is a symptom, not a condition. One way to fix this would be say change the word “hallucinations” to “psychosis”.

105-106 “In this study, we aim to focus squarely on the appraisals and situational factors surrounding a broad set of experiences. When and where are subjectively salient experiences likely to happen?” The word “squarely” doesn’t make sense here. Remove the word “squarely” or change it to something like “in particular” or “specifically”.

In the introduction, there are a number of places where specific examples would make the paper easier to follow. For instance: 120-122: provide an example of the way in which the description of the experience may incorporate an appraisal.

115-116 “to see if there are patterns that can be used to describe the human cognitive system used to make sense of” This sentence is unclear. I think you mean “patterns that can shed light on the cognitive processes involves in making sense of…”

118-120: do you actually mean “conceptual overlap” or do you mean that the causal relationships are difficult to disentangle?

159-160: “We draw on multiple theoretical approaches”. More info is needed. The following section goes on to describe only one theoretical framework.

232-233: “A substantive number of participants in the original validation study thought that chance might be a good reason” The word should be “substantial”, not “substantive” and the sentence overall needs proof-reading.

240-241 I think you are correct that there is a need for broader information about situational factors. But the paper needs to explicitly make the case for WHY this information is needed. Overall, there needs to be a clearer explanation given as to why Taves et al.’s original list of appraisal options is insufficient.

245-253: More information is needed about how this study’s list of new appraisal options were generated from the previous list.

256. Explain what you mean by “based on these insights”. Which insights? Did you take all the appraisal options listed in these papers and divide them into the categories that you outline? Please explain the process in greater detail. Here, once again, the explanation would benefit from reference to specific examples.

381: Change “till” to “until”

403: Explain (perhaps via a brief example) the distinction between cause and reason

411-413: I think you mean “selected by a small number of participants”. If so, change to make clearer.

424 There are words missing in the title: appraisals across what?

439: Should be “a unique” not “an unique”

**********

6. PLOS authors have the option to publish the peer review history of their article (what does this mean?). If published, this will include your full peer review and any attached files.

If you choose “no”, your identity will remain anonymous but your review may still be made public.

**Do you want your identity to be public for this peer review?**  For information about this choice, including consent withdrawal, please see our Privacy Policy.

Reviewer #1: No

Reviewer #2: No

**********

[NOTE: If reviewer comments were submitted as an attachment file, they will be attached to this email and accessible via the submission site. Please log into your account, locate the manuscript record, and check for the action link "View Attachments". If this link does not appear, there are no attachment files.]

To ensure your figures meet our technical requirements, please review our figure guidelines: https://journals.plos.org/plosone/s/figures

You may also use PLOS’s free figure tool, NAAS, to help you prepare publication quality figures: https://journals.plos.org/plosone/s/figures#loc-tools-for-figure-preparation.

NAAS will assess whether your figures meet our technical requirements by comparing each figure against our figure specifications.

# Author comment: Author response to Decision Letter 1

**DOI:** 10.1371/journal.pone.0353126.r002

18 Jan 2026

Dear Dr. Fischer,

Thank you for submitting your manuscript to PLOS ONE.

After careful consideration, we feel that it has a great deal of merit but some revisions are required. Therefore, we invite you to submit a revised version of the manuscript that addresses the points raised during the review process.

_______________________

Dear Dr David Smailes,

Thank you and the two reviewers for your constructive and encouraging comments on our paper now entitled “Making sense of the extraordinary – An investigation of the content and prevalence of appraisals of nonordinary experiences”. We appreciate the opportunity to strengthen our manuscript. Addressing your comments has allowed us to improve clarity and precision in our arguments and presentation of the data. If there are any remaining issues, please do not hesitate to contact us.

=======================================

The reviewers have provided a really useful set of comments that – when addressed – should improve the manuscript. In addition to the comments from the reviewers, please consider the comments from me below.

Sub01AE1 = I think it would be very helpful for readers if a simple hyperlink (such as https://doi.org/10.1371/journal.pone.0287780.s001) was inserted in the MS, that ‘takes’ readers to a copy of the original INOE. This would make it easier for the reader to get a good understanding of the range of experiences classed as an ‘NOE’ (although that may just reflect my lack of familiarity with this concept).

_______________________

Our reply:

Thank you for this great suggestion. We now included a link to the original study in the abstract, and we also included the full INOE as well as annotated changes to the appraisal section made in both English and Portuguese on the OSF and supporting information (Tables S1, S2 and S3). This should allow readers to gain a good understanding of the original inventory and the changes that we made.

Sub01AE2 = On line 355, the study’s pre-registration is mentioned and a citation is provided for that pre-registration. However, I do not think that the document cited is a pre-registration (although it may contain info about the pre-registration). I think this - https://osf.io/ze6cb/overview – link needs to be provided at line 355 instead? Apologies if I am mistaken.

_______________________

Our reply:

Thank you for highlighting this omission. You are correct about the missing link. We have now incorporated the link directly into the body of the text at line 457 to ensure readers can easily access the pre-registration of study 1.

Sub01AE3 = On lines 752-753, two papers are cited re: predictive coding and source-monitoring theories of hallucinations. I am not sure, however that these are particularly effective publications to cite here. Other papers – such as https://academic.oup.com/schizophreniabulletin/article/38/4/683/1870142 or https://www.cell.com/trends/cognitive-sciences/fulltext/S1364-6613(18)30282-1 or https://www.annualreviews.org/content/journals/10.1146/annurev-clinpsy-032816-045145 - may be better to cite (although it does depend on the precise point you are making; especially if the aim is to cite a paper that supports the claim that being alone/socially isolated may affect predictive processing/source monitoring in a way that increases the likelihood of a hallucinatory experience).

_______________________

Our reply:

We appreciate the valuable input and excellent reference suggestions. We have now included citations to the three papers mentioned to support our claim regarding the contextual factor of being alone increasing the likelihood of hallucinations and further expand our argumentation regarding our claim (lines 895 to 902).

Sub01AE4 = Can you please make the OSF project page associated with the manuscript 'public' please? PLOS ONE operates single-blind peer review (reviewers can remain anonymous; authors do not), and so making the OSF project page will not 'unblind' the reviewers. The study data/materials were made available to me after you submitted the manuscript, but I think this was by adding me as a contributor to the project (I have now removed myself from the project, so that it is clear that I have no conflict of interest re: me acting as editor for this manuscript), and I believe the necessary data/scripts are available on the OSF. It would be great if the reviewers could examine this, too, when they review your resubmission.

_______________________

Our reply:

Thank you for this suggestion. We have now made all pages associated with this manuscript freely available. This includes the following links:

Data and scripts for studies 1 and 2: https://osf.io/3fbx4/

Validation method (study 1) preregistration: https://osf.io/ze6cb

Description of data collection for study 2: https://osf.io/t3q2v

=======================================

When submitting your revision, we need you to address these additional requirements.

1. Please ensure that your manuscript meets PLOS ONE's style requirements, including those for file naming. The PLOS ONE style templates can be found at

https://journals.plos.org/plosone/s/file?id=wjVg/PLOSOne_formatting_sample_main_body.pdf and

https://journals.plos.org/plosone/s/file?id=ba62/PLOSOne_formatting_sample_title_authors_affiliations.pdf.

_______________________

Our reply:

Thank you for these links, we have now carefully checked all style requirements and revised our manuscript accordingly.

2. Thank you for stating in your Funding Statement:

“The work was supported by a grant from the John Templeton Foundation (Grant ID: 62651), the Pioneer Science Initiative of the D’Or Institute for Research and Education, Rio de Janeiro, Brazil and a grant by the Conselho Nacional de Desenvolvimento Científico e Tecnologico (Process: 309085/2023-3) to the last author.”

Please provide an amended statement that declares *all* the funding or sources of support (whether external or internal to your organization) received during this study, as detailed online in our guide for authors at http://journals.plos.org/plosone/s/submit-now.

Please also include the statement “There was no additional external funding received for this study.” in your updated Funding Statement.

_______________________

Our reply:

Thank you, we have now updated our funding statement as requested.

3. Thank you for stating the following financial disclosure:

“The work was supported by a grant from the John Templeton Foundation (Grant ID: 62651), the Pioneer Science Initiative of the D’Or Institute for Research and Education, Rio de Janeiro, Brazil and a grant by the Conselho Nacional de Desenvolvimento Científico e Tecnologico (Process: 309085/2023-3) to the last author.”

Please state what role the funders took in the study. If the funders had no role, please state: "The funders had no role in study design, data collection and analysis, decision to publish, or preparation of the manuscript."

If this statement is not correct you must amend it as needed.

Please include this amended Role of Funder statement in your cover letter; we will change the online submission form on your behalf.

_______________________

Our reply:

We have now amended our statement and declare that the funding bodies had no involvement in the design of the study, the collection, analysis, or interpretation of the data, nor in the preparation of the manuscript.

4. Please be informed that funding information should not appear in the Acknowledgments section or other areas of your manuscript. We will only publish funding information present in the Funding Statement section of the online submission form. Please remove any funding-related text from the manuscript.

_______________________

Our reply:

Thank you, we have now removed this section from our title page.

5. Your ethics statement should only appear in the Methods section of your manuscript. If your ethics statement is written in any section besides the Methods, please delete it from any other section.

_______________________

Our reply:

We have removed this information from the title page and included the approval numbers in the relevant sections of the two studies.

6. We note that you have referenced (Fischer R, Bortolini G., Bortolini TS, Maraldi EO, ED, Hartle L, et al. Revisiting the Unusual: ) which has currently not yet been accepted for publication. Please remove this from your References and amend this to state in the body of your manuscript: (Fischer R, Bortolini G., Bortolini TS, Maraldi EO, ED, Hartle L, et al. [Unpublished]”) as detailed online in our guide for authors

http://journals.plos.org/plosone/s/submission-guidelines#loc-reference-style.

_______________________

Our reply:

Thank you, we have updated the reference and now cite the relevant preprint (as per your guidelines, reference can include: Manuscripts on preprint servers, providing the manuscript has a citable DOI or arXiv URL).

7. Please include captions for your Supporting Information files at the end of your manuscript, and update any in-text citations to match accordingly. Please see our Supporting Information guidelines for more information: http://journals.plos.org/plosone/s/supporting-information.

_______________________

Our reply:

We apologize for the lack of clarity. We have reformatted our supporting information following the referred guidelines. Our previous “supplementary material” file was thus broken down into four different PDF files, all of which are captioned in the last section of our manuscript and are accordingly cited throughout the text.

8. If the reviewer comments include a recommendation to cite specific previously published works, please review and evaluate these publications to determine whether they are relevant and should be cited. There is no requirement to cite these works unless the editor has indicated otherwise.

_______________________

Our reply:

Thank you for giving us the liberty to decide whether to include specific suggestions.

We have added a total of 50 references (highlighted in yellow in the text of the revised manuscript) and removed 7 references.

Much of the added references relate to the requests to elaborate on the sources and process of the item generation (previously only available in the supporting information, now S1 Text file). In addition, we have included the references suggested by the editor and the reviewers. Thank you for these great pointers to relevant literature.

In this revision, the following papers were included:

1. Aquino TAA de, Correia APM, Marques ALC, Souza CG de, Assis Freitas HC de, Araújo IF de, et al. Atitude religiosa e sentido da vida: um estudo correlacional. Psicol Ciênc E Prof. 2009;29:228–43.

2. Behr D, Braun M, Aiglstorfer L. Showcasing the usefulness of web probing: Do subtle variations in questionnaire translation lead to different survey responding? Qual Quant. 2024 Oct 1;58(5):4117–35.

3. Blackmore SJ. A psychological theory of the out-of-body experience. J Parapsychol. 1984;48(3):201–18.

4. Bortolini T, Laport MC, Bortolini GN, de Sá CA, Fischer R, Moll J. Mapping the Content Landscape of Self-Transcendent Emotional Experiences Using a Mixed-Method Approach [Internet]. PsyArXiv; 2026 [cited 2026 Jan 16]. Available from: https://osf.io/preprints/psyarxiv/rsy4b_v1/

5. Brett CMC, Johns LC, Peters EP, McGuire PK. The role of metacognitive beliefs in determining the impact of anomalous experiences: a comparison of help-seeking and non-help-seeking groups of people experiencing psychotic-like anomalies. Psychol Med. 2009 June;39(6):939–50.

6. Callaghan BL, Tottenham N. The stress acceleration hypothesis: Effects of early-life adversity on emotion circuits and behavior. Curr Opin Behav Sci. 2016;7:76–81.

7. Cardeña E, Schaffler Y. “He Who Has the Spirits Must Work a Lot”: A Psycho‐Anthropological Account of Spirit Possession in the Dominican Republic. Ethos. 2018 Dec;46(4):457–76.

8. Collao LD, Peñalba A. Cognición, experiencia musical y conciencias alteradas: la influencia de las ciencias cognitivas en el estudio sobre la música y el trance. El Oído Pensante [Internet]. 2019 Feb 1 [cited 2025 June 16];7(1). Available from: http://revistascientificas.filo.uba.ar/index.php/oidopensante/article/view/7542

9. Corlett PR, Horga G, Fletcher PC, Alderson-Day B, Schmack K, Powers AR. Hallucinations and Strong Priors. Trends Cogn Sci. 2019 Feb;23(2):114–27.

10. De Boer EM. Out of Body, Loss of Self: Spiritual or Scary? Religions. 2020 Oct 28;11(11):558.

11. Dilevski N, Paterson HM, Walker SA, van Golde C. Adult memory for specific instances of a repeated event: a preliminary review. Psychiatry Psychol Law. 28(5):711–32.

12. Dunn EC, Nishimi K, Gomez SH, Powers A, Bradley B. Developmental timing of trauma exposure and emotion dysregulation in adulthood: Are there sensitive periods when trauma is most harmful? J Affect Disord. 2018 Feb;227:869–77.

13. Eisenberg ML, Zacks JM, Flores S. Dynamic prediction during perception of everyday events. Cogn Res Princ Implic. 2018 Dec 29;3(1):53.

14. Esperandio MRG, Marques LF. The Psychology of Religion in Brazil. Int J Psychol Relig. 2015 Oct 2;25(4):255–71.

15. Fischer R, Bortolini G, Bortolini T, Maraldi E de O, Hartle L, Varejao MVDL, et al. Revisiting the Unusual: Investigating the Prevalence and Validity of Nonordinary Experiences as a Window into Consciousness [Internet]. In Review; 2025 [cited 2025 Dec 17]. Available from: https://www.researchsquare.com/article/rs-6907742/v1

16. Franco Corso SJ, O’Malley KY, Subaiya S, Mayall D, Dakwar E. The role of non-ordinary states of consciousness occasioned by mind-body practices in mental health illness. J Affect Disord. 2023 Aug 15;335:166–76.

17. Gaynor K, Ward T, Garety P, Peters E. The role of safety-seeking behaviours in maintaining threat appraisals in psychosis. Behav Res Ther. 2013 Feb;51(2):75–81.

18. Griffin JD, Fletcher PC. Predictive Processing, Source Monitoring, and Psychosis. Annu Rev Clin Psychol. 2017;13(1):265–89.

19. Havik G, Elands BHM, van Koppen CSA (Kris). An encounter with one’s deeper self and energy: A phenomenological study among spiritually engaged individuals in the Netherlands. Ecopsychology. 2015;7(2):75–83.

20. Hoemann K, Xu F, Barrett LF. Emotion words, emotion concepts, and emotional development in children: A constructionist hypothesis. Dev Psychol. 2019 Sept;55(9):1830–49.

21. Ip EH, Magee MF, Youssef GA, Chen SH. Gleaning Information for Cognitive Operations from Don’t Know Responses in Cognitive and Noncognitive Assessments. Multivar Behav Res. 2019;54(2):159–72.

22. Irwin HJ, Schofield MB, Baker IS. Dissociative tendencies, sensory-processing sensitivity and aberrant salience as predictors of anomalous experiences and paranormal attributions. J Soc Psych Res. 2014;78(917[4]):193–206.

23. Kuhn W. Understanding religious syncretism in Brazil: Cases in dual allegiance with implications for adventist mission. Rev Hermenêutica [Internet]. 2007 Jan 15 [cited 2025 June 16];7. Available from: https://adventista.emnuvens.com.br/hermeneutica/article/view/205

24. Laurens KR, Hobbs MJ, Sunderland M, Green MJ, Mould GL. Psychotic-like experiences in a community sample of 8000 children aged 9 to 11 years: an item response theory analysis. Psychol Med. 2012 July;42(7):1495–506.

25. Lopez MA. Significados de experiências de devoção: a crença em anjos. Memo Mem E História Em Psicol. 2010 Oct 26;19:26–39.

26. Maraldi E de O, Machado FR, Zangari W. Importance of a Psychosocial Approach for a Comprehensive Understanding of Mediumship. J Sci Explor 2010 Vol 24 Issue 2 P181. 2010;

27. Mason O, Claridge G. The Oxford-Liverpool Inventory of Feelings and Experiences (O-LIFE): Further description and extended norms. Schizophr Res. 2006 Feb;82(2–3):203–11.

28. Monteiro de Barros MC, Leão FC, Vallada Filho H, Lucchetti G, Moreira-Almeida A, Prieto Peres MF. Prevalence of spiritual and religious experiences in the general population: A Brazilian nationwide study. Transcult Psychiatry. 2022 Apr 6;13634615221088701.

29. Moreira-Almeida A, Cardeña E. Differential diagnosis between non-pathological psychotic and spiritual experiences and ment

Attachment Submitted filename: appraisals_review_letter_plosone [16-01-2026]_18h.docx

# Peer review: Decision Letter 1

**DOI:** 10.1371/journal.pone.0353126.r003

**Authors:** David Smailes, David Smailes

18 Jun 2026

Making sense of the extraordinary – An investigation of the content and prevalence of appraisals of nonordinary experiences

PONE-D-25-47007R1

Dear Dr. Fischer,

We’re pleased to inform you that your manuscript has been judged scientifically suitable for publication and will be formally accepted for publication once it meets all outstanding technical requirements. It's a really strong paper, which I think will be very valuable for researchers working across a nmber of different fields.

I am very sorry for the delay in obtaining reviews of your re-submission. I extended the deadline for one reviewer twice, as they had provided such a careful review of your first submission, and this was the cause of the delay.

Within one week, you’ll receive an e-mail detailing the required amendments. When these have been addressed, you’ll receive a formal acceptance letter and your manuscript will be scheduled for publication.

An invoice will be generated when your article is formally accepted. Please note, if your institution has a publishing partnership with PLOS and your article meets the relevant criteria, all or part of your publication costs will be covered. Please make sure your user information is up-to-date by logging into Editorial Manager at Editorial Manager® and clicking the ‘Update My Information' link at the top of the page. For questions related to billing, please contact billing support.

If your institution or institutions have a press office, please notify them about your upcoming paper to help maximize its impact. If they’ll be preparing press materials, please inform our press team as soon as possible -- no later than 48 hours after receiving the formal acceptance. Your manuscript will remain under strict press embargo until 2 pm Eastern Time on the date of publication. For more information, please contact onepress@plos.org.

Kind regards,

David Smailes, Ph.D.

Academic Editor

PLOS One

Additional Editor Comments:

Thank you for the detailed and clear responses to the reviewers' comments. All comments were responded to effectively.

Reviewers' comments:

Reviewer's Responses to Questions

**Comments to the Author**

1. If the authors have adequately addressed your comments raised in a previous round of review and you feel that this manuscript is now acceptable for publication, you may indicate that here to bypass the “Comments to the Author” section, enter your conflict of interest statement in the “Confidential to Editor” section, and submit your "Accept" recommendation.

Reviewer #1: All comments have been addressed

**********

2. Is the manuscript technically sound, and do the data support the conclusions?

The manuscript must describe a technically sound piece of scientific research with data that supports the conclusions. Experiments must have been conducted rigorously, with appropriate controls, replication, and sample sizes. The conclusions must be drawn appropriately based on the data presented.

Reviewer #1: Yes

**********

3. Has the statistical analysis been performed appropriately and rigorously?

Reviewer #1: Yes

**********

4. Have the authors made all data underlying the findings in their manuscript fully available?

The PLOS Data policy requires authors to make all data underlying the findings described in their manuscript fully available without restriction, with rare exception (please refer to the Data Availability Statement in the manuscript PDF file). The data should be provided as part of the manuscript or its supporting information, or deposited to a public repository. For example, in addition to summary statistics, the data points behind means, medians and variance measures should be available. If there are restrictions on publicly sharing data—e.g. participant privacy or use of data from a third party—those must be specified.

Reviewer #1: Yes

**********

5. Is the manuscript presented in an intelligible fashion and written in standard English?

PLOS ONE does not copyedit accepted manuscripts, so the language in submitted articles must be clear, correct, and unambiguous. Any typographical or grammatical errors should be corrected at revision, so please note any specific errors here.

Reviewer #1: Yes

**********

6. Review Comments to the Author

Please use the space provided to explain your answers to the questions above. You may also include additional comments for the author, including concerns about dual publication, research ethics, or publication ethics. (Please upload your review as an attachment if it exceeds 20,000 characters)

Reviewer #1: (No Response)

**********

7. PLOS authors have the option to publish the peer review history of their article (what does this mean?). If published, this will include your full peer review and any attached files.

If you choose “no”, your identity will remain anonymous but your review may still be made public.

**Do you want your identity to be public for this peer review?**  For information about this choice, including consent withdrawal, please see our Privacy Policy.

Reviewer #1: **Yes:**  Keith Gaynor

**********

# Editor report: Acceptance letter

**DOI:** 10.1371/journal.pone.0353126.r004

**Authors:** David Smailes, David Smailes

PONE-D-25-47007R1

PLOS One

Dear Dr. Fischer,

I'm pleased to inform you that your manuscript has been deemed suitable for publication in PLOS One. Congratulations! Your manuscript is now being handed over to our production team.

At this stage, our production department will prepare your paper for publication. This includes ensuring the following:

* All references, tables, and figures are properly cited

* All relevant supporting information is included in the manuscript submission,

* There are no issues that prevent the paper from being properly typeset

You will receive further instructions from the production team, including instructions on how to review your proof when it is ready. Please keep in mind that we are working through a large volume of accepted articles, so please give us a few days to review your paper and let you know the next and final steps.

Lastly, if your institution or institutions have a press office, please let them know about your upcoming paper now to help maximize its impact. If they'll be preparing press materials, please inform our press team within the next 48 hours. Your manuscript will remain under strict press embargo until 2 pm Eastern Time on the date of publication. For more information, please contact onepress@plos.org.

You will receive an invoice from PLOS for your publication fee after your manuscript has reached the completed accept phase. If you receive an email requesting payment before acceptance or for any other service, this may be a phishing scheme. Learn how to identify phishing emails and protect your accounts at https://explore.plos.org/phishing.

If we can help with anything else, please email us at customercare@plos.org.

Thank you for submitting your work to PLOS ONE and supporting open access.

Kind regards,

PLOS ONE Editorial Office Staff

on behalf of

Dr. David Smailes

Academic Editor

PLOS One
